# piRNA-1742 promotes renal cell carcinoma malignancy by regulating USP8 stability through binding to hnRNPU and thereby inhibiting MUC12 ubiquitination

**DOI:** 10.1038/s12276-023-01010-3

**Published:** 2023-06-19

**Authors:** Wentao Zhang, Zongtai Zheng, Keyi Wang, Weipu Mao, Xue Li, Guangchun Wang, Yuanyuan Zhang, Jianhua Huang, Ning Zhang, Pengfei Wu, Ji Liu, Haimin Zhang, Jianping Che, Bo Peng, Junhua Zheng, Wei Li, Xudong Yao

**Affiliations:** 1 Department of Urology, Shanghai Tenth People’s Hospital, Tongji University, Shanghai, P. R. China; 2 Urologic Cancer Institute, School of Medicine, Tongji University, Shanghai, P. R. China; 3 Department of Urology, Guangdong Second Provincial General Hospital, Guangzhou, P. R. China; 4 Department of Urology, Affiliated Zhongda Hospital of Southeast University, Nanjing, P. R. China; 5 Department of Pathology, Beijing Chao-Yang Hospital, Capital Medical University, Beijing, P. R. China; 6 Institute for Regenerative Medicine, Wake Forest University, Winston-Salem, NC USA; 7 Department of Medical Oncology, Dana-Farber Cancer Institute, Harvard Medical School, Boston, MA USA; 8 State Key Laboratory of Oncogenes and Related Genes, Shanghai Jiao Tong University, Shanghai, P. R. China; 9 Department of Urology, Renji Hospital, School of Medicine, Shanghai Jiao Tong University, Shanghai, P. R. China

**Keywords:** Targeted therapies, Cancer prevention

## Abstract

Accumulating studies have confirmed that PIWI-interacting RNAs (piRNAs) are considered epigenetic effectors in cancer. We performed piRNA microarray expression analysis on renal cell carcinoma (RCC) tumor tissues and paired normal tissues and performed a series of in vivo and in vitro experiments to explore piRNAs associated with RCC progression and investigate their functional mechanisms. We found that piR-1742 was highly expressed in RCC tumors and that patients with high piR-1742 expression had a poor prognosis. Inhibition of piR-1742 significantly reduced tumor growth in RCC xenograft and organoid models. Mechanistically, piRNA-1742 regulates the stability of USP8 mRNA by binding directly to hnRNPU, which acts as a deubiquitinating enzyme that inhibits the ubiquitination of MUC12 and promotes the development of malignant RCC. Subsequently, nanotherapeutic systems loaded with piRNA-1742 inhibitors were found to effectively inhibit the metastasis and growth of RCC in vivo. Therefore, this study highlights the functional importance of piRNA-related ubiquitination in RCC and demonstrates the development of a related nanotherapeutic system, possibly contributing to the development of therapeutic approaches for RCC.

## Kidney cancer: Small regulatory RNA promotes progression

A small RNA molecule regulates key cellular processes and promotes the malignancy of renal cell cancer, the most common kidney cancer in adults, in ways that could be targeted by anti-cancer treatments. The incidence of renal cell cancer is steadily increasing, and patients with high levels of piRNA-1742 have poorer outcomes. Wentao Zhang and colleagues at Tongji University, Shanghai, and other research centers in China, found that the cancer-promoting abilities of piRNA-1742 involve its binding to a protein called hnRNPU which controls several aspects of biomolecular processing. The net effect of piRNA-1742 activity is to enhance the ability of renal cancer cells to spread and invade other tissues. Trials in mice suggest that piRNA-1742 might be a useful biomarker for predicting cancer progression and that inhibiting its activity could be a useful therapeutic approach.

## Introduction

In recent years, a progressive increase has been observed in the incidence of renal cell carcinoma (RCC) in the United States, Europe, and Asia, with current annual incidence rates of approximately 3%. In addition, approximately 92,000 deaths owing to RCC are reported worldwide annually^1,2^. The incidence of renal cancer has shown two following trends: one is that the incidence is increasing year by year, and the other is a trend toward diagnosis at younger ages^3^. Metastatic lesions are observed in approximately 30% of patients at initial diagnosis, and >30% of patients eventually develop metastatic RCC (mRCC) postoperatively^4^. In patients with metastatic RCC, existing treatments often fail to successfully suppress tumor progression or establish remission^5,6^. Currently, available treatment approaches have response rates of 12% and 30%, with the overall median survival time of mRCC patients being <14.6 months^7,8^. Understanding the molecular pathways involved in the onset and progression of RCC is crucial for the development of more effective treatment options.

P-element-induced wimpy testis (PIWI)-interacting RNAs (piRNAs) are subsets of newly identified small noncoding RNAs in germ and somatic cells that contain 24–31 nucleotides (nt) with an adenosine bias at the 10th position or 5′-terminal uridine and lack clear secondary structure motifs^9,10^. The piRNA pathway is composed of piRNAs that interact with PIWI proteins, during which the pre-piRNAs are transcribed from their clusters, cleaved by PIWI proteins and eventually amplified in the cytoplasm via a cycle that is reliant on sequence complementarity^11^. Initially, it was discovered that the piRNA-PIWI protein pathway was implicated in the protection of the germline genome from insertional mutations caused by transposons^12^. However, recent research has shown that piRNAs could also play a role at the somatic level, modulating gene expression via ubiquitination or DNA methylation^13,14^. piRNAs not only perform important functions in germline development but also perform an instrumental function in tumorigenesis^15,16^. The roles of piRNAs in controlling epigenetic function and in human cancers are currently unclear but present a new direction for basic and translational research^17^.

In this research, we discovered piRNA 1742 (piR-1742) to be a carcinogenic piRNA that performs a critical function in the onset and progression of RCC. Biochemical assays demonstrated that piR-1742 precisely binds to hnRNPU, facilitating the formation of the piRNA/hnRNPU/USP8 complex and the deubiquitination of MUC12, which consequently mediates the ability of RCC cells to invade and metastasize. We also constructed a nanodrug delivery system based on piR-1742 and MUC12 and performed preliminary validation in mice. These results imply that the carcinogenic piR-1742 gene could be a potential treatment target for RCC and that it might also be useful as a biological marker for predicting clinical outcomes.

## Methods

### Clinical specimens

RCC samples were obtained from individuals who had undergone nephrectomy or partial nephrectomy at Beijing Chao-Yang Hospital of Capital Medical University and Shanghai Tenth People’s Hospital of Tongji University. Supplementary Tables 1, 2 summarize the clinical features of the patients involved in the research. The Shanghai Tenth People’s Hospital of Tongji University’s Ethics Committee examined and approved all experiments involving human subjects before they were conducted (Approval number: 2021KN221).

### Cell lines and cell culture

The Chinese Academy of Sciences (Shanghai, China) supplied the RCC cell lines 786-O (RRID: CVCL_1051),769-P(RRID: CVCL_1050), Caki-1 (RRID: CVCL_0234), Caki-2 (RRID: CVCL_0235), A498 (RRID: CVCL_1056), ACHN (RRID: CVCL_1067), and OS-RC2 (RRID: CVCL_1626) and the normal renal tubular epithelial cell line HK-2 (RRID: CVCL_0302). All cell lines were grown in the recommended medium containing 10% fetal bovine serum (FBS; Thermo Fisher Scientific,10100147,USA) and 1% penicillin/streptomycin (HyClone, Logan, UT, USA) and were incubated in a chamber containing 5% CO_2_ at 37 °C.

### RCC Organoids

Tumor tissues were obtained by radical nephrectomy or partial nephrectomy. They were cut into 1–2-mm-thick sections before digestion with collagenase type IV (1 mg/mL, C9891, Sigma-Aldrich) and Y-27632 (HY-10071, MCE, 10 μM) for 1 h. The suspension was filtered through a 70-μm mesh. The pellet was resuspended in 200 μL of Basement Membrane Extract (BME, 3533-001-02, R&D) and seeded into prewarmed 12-well plates. After the BME solidified, human renal cancer organoid medium was added for culture.

### Nomogram construction

The independent survival indicators were examined utilizing both multivariate and univariate Cox regression analyses. Significant variables in the multivariate Cox regression analysis were utilized to develop a nomogram. Calibration plots were generated utilizing the ‘Rms’ R package v5.1 to evaluate the nomogram’s accuracy. The nomogram was subsequently subjected to decision curve analysis (DCA) to ascertain its clinical value. Next, the nomogram was checked for reliability in predicting one-, three-, and five-year survival using receiver operating characteristic curves (ROC curves).

### Uptake of pPM-NPs

The uptake of pPM-NPs was examined via bio-TEM^18^. Briefly, cells were seeded in a six-well plate and incubated for 24 h to 60% confluence. Thereafter, the cells were treated with pPM-NPs (2 μM) suspended in fresh culture medium for 6 h and collected after washing and detachment. Subsequently, the cells were resuspended and incubated in glutaraldehyde fixative (2.5%) at 4 °C overnight. Following preparation, the cells were washed and dehydrated before being subjected to polymerization. Spurr’s low-viscosity solution was used for polymerization at 60°C to prepare the samples. Finally, the samples were sliced and stained with lead citrate and examined via bio-TEM.

### Statistical analysis

SPSS 19.0 software (RRID: SCR_002865, SPSS, Inc., Chicago, USA) was utilized to process all statistical data. Pearson correlation analysis was employed to determine the degree of correlation. The paired two-tailed *t* test was adopted to evaluate the differences between the groups. The links between piR-1742 expression and other characteristics were examined utilizing one-way analysis of variance (ANOVA) or the nonparametric Kruskal‒Wallis test. Kaplan‒Meier analysis was applied to generate survival curves, and the differences in survival were investigated utilizing the log-rank test. To identify independent components, a Cox proportional hazards model was implemented including the factors that were identified via univariate analysis. The experimental data are presented as the means ± SDs, and statistically significant differences were determined to be those with *p* values of less than 0.05: *p < 0.05, **p < 0.01.

## Results

### piR-1742 is overexpressed and correlates with a poor prognosis in RCC

**Fig. 1:**
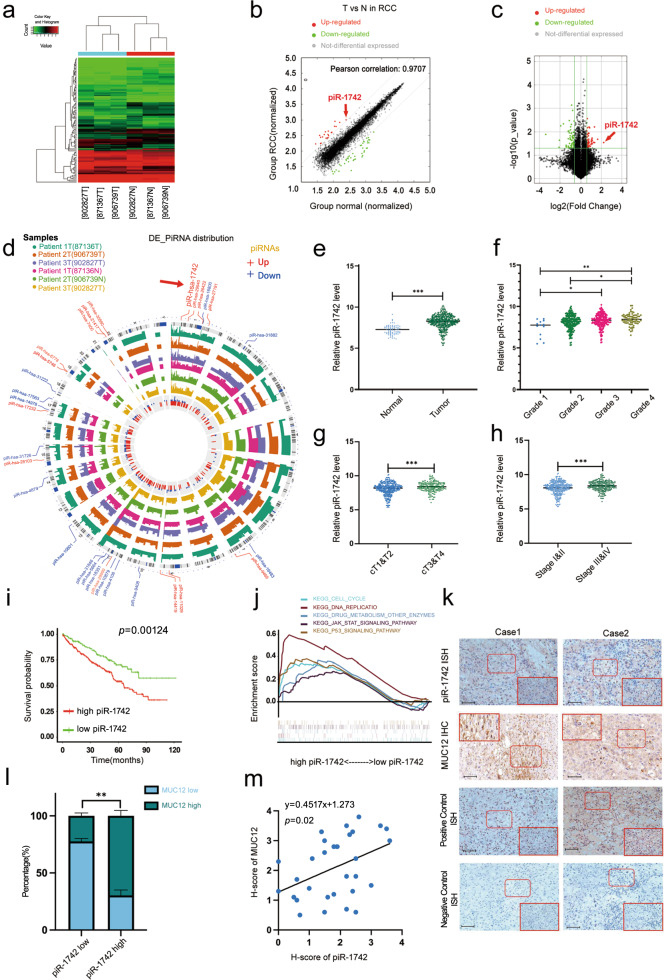
piRNA expression profiles in RCC and characterization of piR-1742. **a** Heatmap showing differentially expressed piRNAs. **b** Symmetric scatter plot showing differentially expressed piRNAs between RCC and normal samples. **c** Volcano plot showing differential expression of piRNAs between RCC and normal samples. **d** The expression levels and chromosomal locations of the top 15 upregulated piRNAs and downregulated piRNAs. **e** piR1742 expression levels in RCC tumors and corresponding normal samples in TCGA. The expression of piR1742 in tumors of different grades and stages: tumor grade (**f**), tumor stage (**g**) and clinical T stage (**h**). **i** Kaplan‒Meier analysis of RCC patients with low versus high expression levels of piR-1742. **j** GSEA of piR-1742. **k-l** Representative images of piR-1742 ISH and IHC of MUC12 and percentages of piR-1742 and MUC12 expression in tissues in RCC patients, Scale bar: 50 μm. **m** Correlation of piR-1742 and MUC12 expression based on the H-score. *p < 0.05, **p < 0.01, ***p < 0.01.

To identify differentially expressed piRNAs in RCC, three pairs of human RCC samples and matched paracancerous normal samples were analyzed utilizing a piRNA microarray (Supplementary Table 3). The heatmap of the piRNA microarray analysis results is shown in Fig. 1a. In total, 53 upregulated and 67 downregulated piRNAs were identified in RCC tissues (Fig. 1b, c). Based on the fold change (FC) values, the top 15 upregulated piRNAs and top 15 downregulated piRNAs were selected, and their expression levels and chromosome positions are shown in Fig. 1d. Because piR-1742 (NCBI Accession: DQ571419) had the highest FC value (FC = 4.696, *p* = 0.03), it was selected for further analyses.

In TCGA, high piR-1742 expression was associated with advanced clinicopathological features, including high tumor grade, clinical stage and T stage (Fig. 1e–h). High piR-1742 expression was also linked to unfavorable survival outcomes in RCC patients with distinct clinical and pathological features, including tumor grade, clinical stage and T stage (Fig. 1i, Supplementary Fig. 1a–f). Additionally, GSEA was employed to examine the impact of piR-1742 on a variety of gene sets with different biological functions. The results of GSEA revealed substantial enrichment of several malignant hallmarks of tumors, including the cell cycle, DNA replication, drug metabolism enzymes, the JAK-STAT pathway and the P53 signaling pathway, in the high piR-1742 expression group (Fig. 1j). We further found that the expression of MUC12 was significantly positively correlated with that of piR-1742 by ISH and IHC assays (Fig. 1k–m).

To further confirm whether piR-1742 acts independently as a prognostic indicator in RCC patients, we performed ROC curve analysis and found that the expression of piR-1742 was differed markedly between RCC and normal tissues, with an AUC of 0.842. The ROC curve also showed that the nomogram had high accuracy for survival prediction (Supplementary Fig. 1g–h). The Kaplan‒Meier curves showed that patients with elevated piR-1742 + MUC12 expression exhibited unfavorable survival outcomes (Supplementary Fig. 1i–l).

**Fig. 2:**
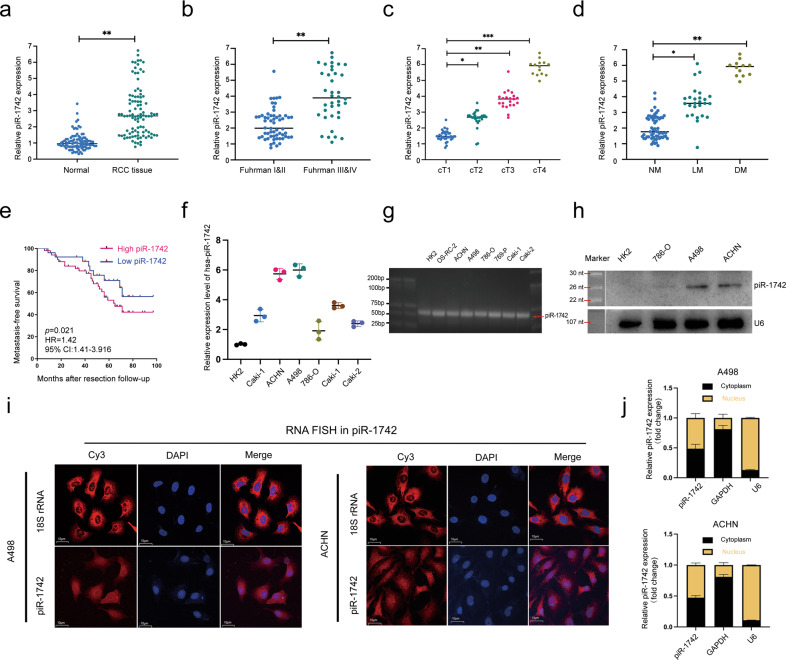
The pattern and significance of piR-1742 expression based on samples in our center. **a** Expression of piR-1742 in RCC and paired normal samples. Expression of piR-1742 in patients stratified by different clinicopathological features: Fuhrman grade (**b**), clinical T stage (**c**) and metastasis status (**d**). **e** Kaplan‒Meier analysis of RCC patients with low versus high expression levels of piR-1742. **f** Expression of piR-1742 in different RCC cell lines compared with that in the normal renal tubular epithelial cell line HK2 by qPCR. **g** Agarose gel electrophoresis was used to validate the presence of piR-1742 in RCC cell lines. **h** Northern blot of piR-1742 in RCC cells and HK2 cells. **i**, **j** FISH assays and nuclear–cytoplasmic RNA fractionation were used to clarify the cellular localization of piR-1742 in A498 and ACHN cells.

We further validated the association of piR-1742 with clinicopathological features and prognosis by analysis of paired tissue samples from 96 RCC patients in our hospital (Supplementary Tables 1, 2). The findings showed considerable enhancement of piR-1742 expression in RCC samples compared with normal samples (Fig. 2a). Patients with advanced clinicopathological features, including Fuhrman grade III/IV, high T stage and metastasis, had significantly higher expression levels of piR-1742 (Fig. 2b–d). In addition, patients in the high piR-1742 expression group exhibited worse OS than those in the low piR-1742 expression group (Fig. 2e).

Furthermore, piR-1742 expression was found to be higher in RCC cells than in normal human renal cell lines (Fig. 2f). The results of agarose gel electrophoresis and northern blotting validated the presence of piR-1742 in the RCC cell lines (Fig. 2g, h). Additionally, nuclear–cytoplasmic RNA fractionation and fluorescence in situ hybridization (FISH) for piR-1742 showed that piR-1742 was distributed in both the nucleus and cytoplasm (Fig. 2i, j). Based on these findings, A498 and ACHN cells, with high piR-1742 expression, and 786-O cells, with low piR-1742 expression, were selected for subsequent experiments.

### piR-1742 induces carcinogenic impacts on renal carcinoma cells in vivo and in vitro

**Fig. 3:**
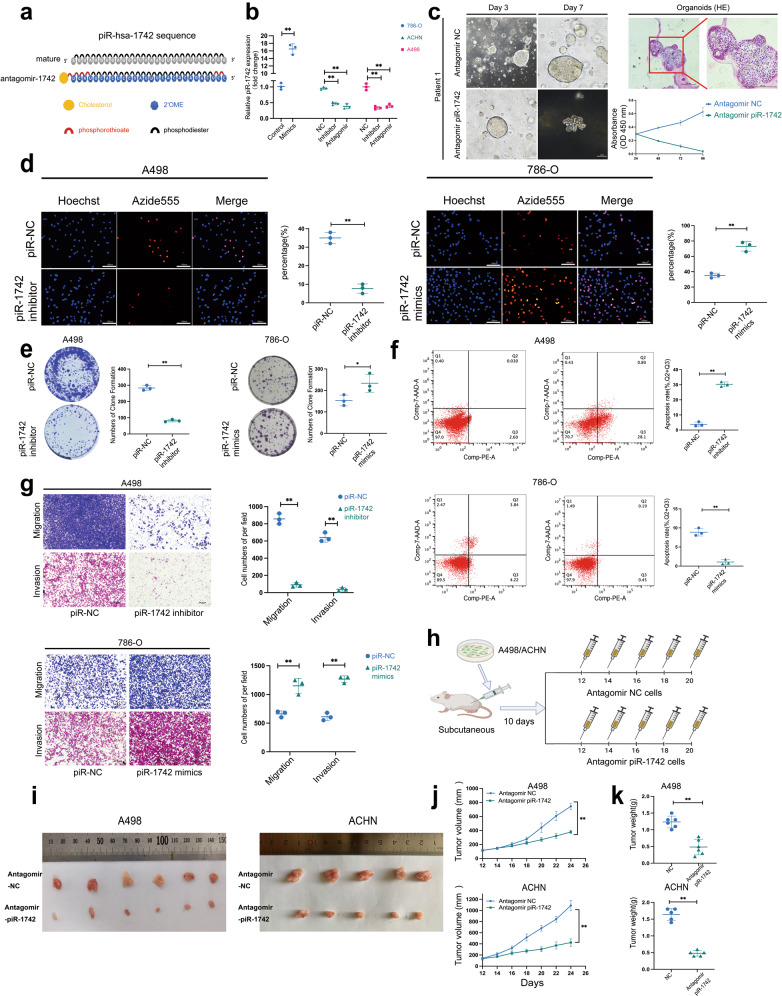
piR-1742 has oncogenic impacts on renal carcinoma cells in vivo and in vitro. **a** Specific modifications and sequence information for the piR-1742 antagomir. **b** Knockdown and overexpression efficiencies of the piR-1742 inhibitor, antagomir and mimics in RCC cells. **c** Renal cancer organoids were constructed and validated by HE staining. The CCK8 assay was used to validate the effect of the piR-1742 antagomir on organoid viability. Scale bars = 100 and 50 μm. **d** Representative images of the EdU incorporation assay utilized to determine the viability of RCC cells treated with the piR-1742 inhibitor/mimics; Scale bars = 10 μm. **e** Representative images of the colony formation assay utilized to measure the proliferative ability of RCC cells treated with piR-1742 inhibitor/mimics. **f** A flow cytometry assay was employed to identify the effects of piR-1742 on the apoptosis of RCC cells. **g** Transwell assays were applied to examine the migration and invasion abilities of RCC cells after piR-1742 suppression or overexpression; scale bars = 100 and 50 μm. **h** Graphical illustration of the nude mouse subcutaneous tumor model. Nude mice were injected with NC antagomir/piR-1742 antagomir every 2 days, and analyses of tumor specimens (**i**), tumor growth (**j**), and tumor weight (**k**) in the mice bearing subcutaneous xenografts were performed. Data are presented as the mean ± SD of three separate tests. *p < 0.05, **p < 0.01.

To elucidate the biological roles of piR-1742 in RCC cells, an inhibitor and mimics of piR-1742 were constructed. Phosphorothioate-modified antagomirs and cholesterol-conjugated, 2’-OMe-modified siRNAs have extremely long lifespans and stability, and gene silencing can be achieved via efficient uptake of antagomirs into cells (Fig. 3a). Therefore, we constructed a piR-1742 antagomir. The qPCR results showed that treatment with both the inhibitor and antagomir of piR-1742 significantly reduced the expression of piR-1742, whereas treatment with the piR-1742 mimics increased the expression of piR-1742 (Fig. 3b).

RCC organoids were employed to examine the impacts of piR-1742 on the viability of tumor cells. The successful construction of renal cancer organoids was validated via HE staining, and the IC50 values of the piR-1742 antagomir in different patient organoids were calculated (Supplementary Fig. 2a–d). CCK8 assays revealed that the viability of organoids was significantly decreased after treatment with the piR-1742 antagomir (Fig. 3c, Supplementary Fig. 2e).

Furthermore, an in vitro EdU incorporation assay revealed that knockdown of piR-1742 reduced the proliferation capacity of A498 cells and that elevated piR-1742 expression levels enhanced the proliferative ability of 786-O cells (Fig. 3d). The same results were obtained in the colony formation assay (Fig. 3e). These results were further validated in ACHN cells (Supplementary Fig. 3a, b). Flow cytometry analysis showed that suppressing piR-1742 enhanced apoptosis, whereas overexpression of piR-1742 inhibited apoptosis (Fig. 3f, Supplementary Fig. 3c). In addition, the transwell assay results further indicated that suppressing piR-1742 expression facilitated the tumor cells’ migratory and invasive capacities, both of which were reduced by piR-1742 overexpression (Fig. 3g). These results were also validated in the ACHN cell line (Supplementary Fig. 3d).

For in vivo experiments, a subcutaneous tumor model was established with renal cancer cells, and beginning 10 days after cell implantation, the mice were injected with the piR-1742 antagomir every 2 days (Fig. 3h). The results showed that piR-1742 could significantly delay tumor proliferation (Fig. 3i–k). HE staining and IHC staining (Ki67, PCNA, and Vimentin) further verified the above results (Supplementary Fig. 3e–h).

### piR-1742 regulates the stability of USP8 mRNA

**Fig. 4:**
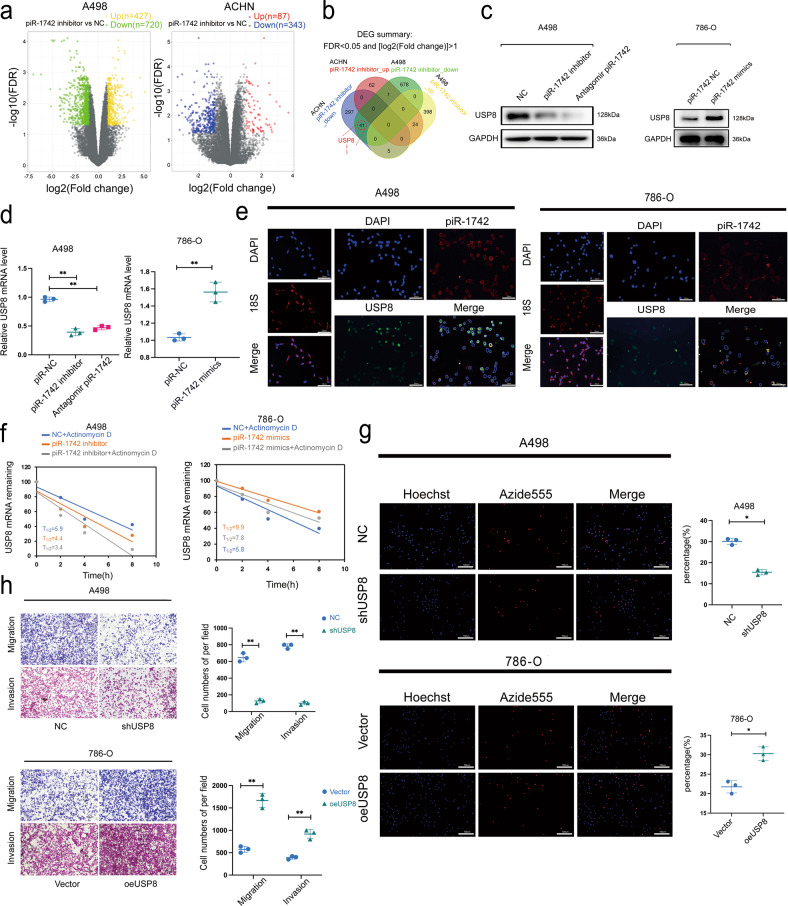
piR-1742 regulates the stability of USP8 mRNA. **a** Volcano plots showing the DEGs between the two cell lines. **b** Venn diagram showing the DEGs between the two cell lines. **c** Western blotting was utilized to determine USP8 protein expression in A498 and 786-O cells following piR-1742 knockdown or overexpression. **d** qPCR was used to evaluate USP8 mRNA expression after piR-1742 overexpression or knockdown in A498 and 786-O cells. **e** FISH was used to determine the colocalization of piR-1742 and USP8 in A498 and 786-O cells. **f** qPCR was used to measure the half-life (t1/2) of USP8 mRNA in A498 and 786-O cells. **g** Representative images of the EdU incorporation assay used to measure the proliferation of A498/786-O cells after USP8 knockdown or overexpression; scale bar = 10 μm. **h** Transwell assays were used to analyze the migration and invasion abilities of A498/786-O cells after USP8 knockdown or overexpression; scale bars = 100 and 50 μm. Data are presented as the mean ± SD of three separate experimental tests *p < 0.05, **p < 0.01.

To clarify the role of piR-1742 in the malignant progression of renal cancer, we performed RNA sequencing analysis on A498 and ACHN cell lines after treatment with the piR-1742 inhibitor (Fig. 4a), and we found that the mRNA level of USP8 was significantly decreased (Fig. 4b, Supplementary Fig. 4a–d). Western blot and qPCR analyses indicated that the mRNA and protein levels of USP8 were significantly decreased after knockdown of piR-1742 (Fig. 4c, d, Supplementary Fig. 4e, f). We also found by FISH that piR-1742 and USP8 were colocalized in A498 and 786-O cells (Fig. 4e). Moreover, we found that knockdown of piR-1742 reduced the stability of USP8 mRNA and promoted its degradation (Fig. 4f, Supplementary Fig. 4g). Furthermore, we overexpressed piR-1742 in the 786-O cell line and obtained the same results.

In addition, USP8 knockdown A498 (shUSP8-A498) and USP8-overexpressing 786-O (oeUSP8-786-O) cell lines were established, and the efficiency of knockdown and overexpression was verified using western blotting and qPCR (Supplementary Fig. 5a, b). The results of EdU incorporation and CCK8 assays showed that USP8 affected the ability of RCC cells to proliferate (Fig. 4g, Supplementary Fig. 5c). Transwell assays and flow cytometry analysis showed that USP8 regulated the apoptotic, migratory and invasive capacities of RCC cells (Fig. 4h, Supplementary Fig. 5d). In the rescue experiment, we introduced piR-1742 mimics into A498 cells with USP8 knockdown, and we found by qPCR and western blotting that the expression of USP8 was rescued. We also overexpressed USP8 in 786-O cells for rescue experiments, and similar results were obtained (Supplementary Fig. 5e, f). These findings demonstrate that piR-1742 may promote RCC progression through USP8.

### piR-1742 stabilizes USP8 mRNA by directly binding to hnRNPU

**Fig. 5:**
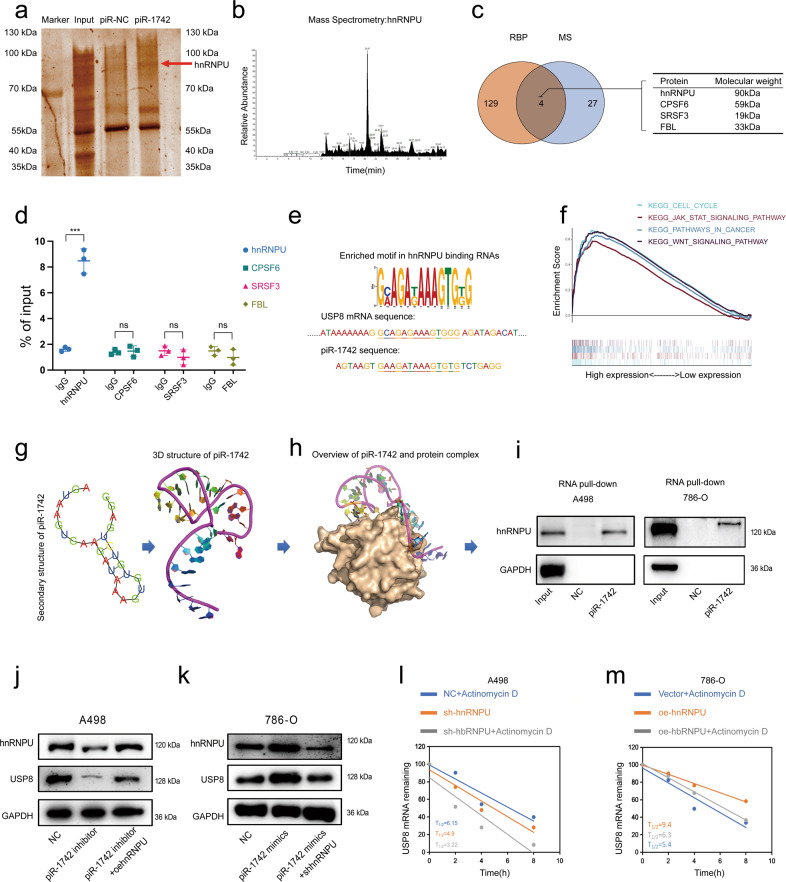
piR-1742 regulates USP8 mRNA stability by directly binding to hnRNPU. **a** RNA pull-down assay with a piR-1742 probe and NC probe. **b-c** Mass spectrometry analysis and Venn diagram showing piR-1742-binding proteins identified by the RNA pull-down assay. **d** qPCR analysis of hnRNPU, CPSF6, SRSF3 and FBL expression in A498 cells after RIP assays. **e** GSEA after hnRNPU modulation. **f** Secondary structure of piR-1742, its predicted tertiary structure, and its predicted binding to hnRNPU. **g** PyMOL software was used to predict the possible amino acids of hnRNPU binding to piR-1742. **h** Western blotting was performed after RNA pull-down in 786-O and A498 cells. **i** Analysis of sequence motifs in USP8 and piR-1742 significantly regulated by hnRNPU by use of a ZOOPS model in MEME software. The GMAGAKAAAGTGKG motif was present in the analyzed RNA sequences. **j**, **k** Western blotting and rescue experiments were used to verify the expression of USP8 and hnRNPU in A498 and 786-O cells. **l**, **m** qPCR was used to measure the half-life (t1/2) of USP8 mRNA in hnRNPU rescue experiments.

To clarify the reason that piR-1742 can stabilize USP8 mRNA in RCC, potential interacting proteins of piR-1742 were screened through an RNA pull-down assay and mass spectrometry (Fig. 5a, b). Experiments with piR-1742 and its antisense transcripts revealed 31 differentially expressed proteins between the two groups (Supplementary Fig. 6a and Supplementary Tables 6, 7), and 4 of them were RNA-binding proteins (RBPs), as defined by starBase (https://starbase.sysu.edu.cn/) (Fig. 5c). Further RIP experiments confirmed that hnRNPU is a possible binding protein of piR-1742, while CPSF6, SRSF3 and FBL may exhibit nonspecific binding (Fig. 5d). The Multiple EM for Motif Elicitation tool (MEME, http://meme-suite.org/tools/meme) was used to identify sequence motifs affected by RNAs significantly regulated by hnRNPU. We found that in USP8 mRNA and piRNAs, the motif GMAGAKAAAGTGKG was significantly enriched (Fig. 5e). GSEA showed a marked link between hnRNPU expression and the pathways involved in malignant progression of RCC, such as the apoptosis and WNT pathways (Fig. 5f). The expression of hnRNPU was significantly decreased after the treatment of mouse subcutaneous xenografts with antagomir-1742, as shown by IHC (Supplementary Fig. 6b).

Moreover, we predicted the three-dimensional (3D) structure of piR-1742 through analysis of its secondary structure (Fig. 5g) and determined the 3D interaction structure of piR-1742 and hnRNPU (Fig. 5h) using PyMOL software. Western blot analysis after pull-down verified that piR-1742 could bind to hnRNPU (Fig. 5i).

We constructed RCC cell lines with knockdown or overexpression of hnRNPU and verified the knockdown and overexpression efficiency by qPCR (Supplementary Fig. 6c). Further rescue experiments showed that overexpression of piR-1742 in the A498 cell line with hnRNPU knockdown could restore the expression of USP8, and the corresponding conclusion was obtained in 786-O cells overexpressing hnRNPU (Fig. 5j, k, Supplementary Fig. 6d). hnRNPU is a classical RBP that can regulate mRNA stability. The RIP assay results also confirmed that hnRNPU can directly bind to USP8 (Supplementary Fig. 6e). We also verified whether piR-1742 regulates the stability of USP8 through hnRNPU through a rescue experiment. We found that treatment with shRNPU + the piR-1742 mimics increased the stability of USP8 mRNA, and the corresponding conclusion was obtained in 786-O cells overexpressing hnRNPU (Fig. 5l, m). Additionally, we found by western blotting that knockdown of hnRNPU in A498 cells could reduce the expression of USP8, VEGF, etc. (Supplementary Fig. 6f). Knockdown of hnRNPU also inhibited the migration and invasion of A498 cells, as shown by transwell assays (Supplementary Fig. 6g, h). Conversely, overexpression of hnRNPU promoted USP8 expression in 786-O cells and enhanced their invasion. The above experiments show that piR-1742 can bind to hnRNPU, an RNA-binding protein, thereby stabilizing the mRNA of USP8.

### USP8 upregulates MUC12 expression through deubiquitination

**Fig. 6:**
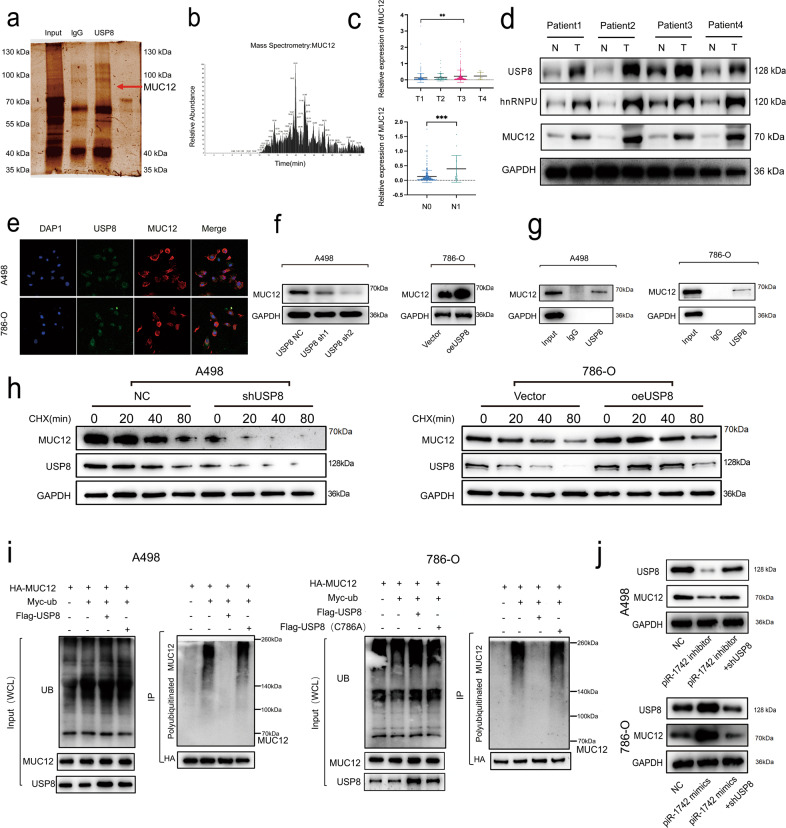
USP8 binds to MUC12 and regulates its ubiquitination. **a**, **b** The results of Co-IP and mass spectrometry confirmed that USP8 can directly bind to MUC12. **c** Expression of MUC12 in patients with different clinicopathological features by N stage and T stage. **d** The expression of MUC12, USP8, and hnRNPU in RCC samples by using western blotting. **e** Representative immunofluorescence images showing the colocalization of MUC12 and USP8; scale bar = 10 μm. **f** Western blotting was utilized to verify MUC12 expression following knockdown or overexpression of USP8. **g** A co-IP assay was used to verify the binding of USP8 to MUC12. **h** Western blotting was utilized to identify the expression of USP8 and MUC12 at different time points following CHX treatment. **i** Construction of a USP8-inactive mutant plasmid and determination of the MUC12 ubiquitination level via Co-IP after treatment with MG132. **j** Western blot analysis showed that the piR-1742-regulated expression of MUC12 could be rescued by USP8.

As one of the deubiquitinating enzymes, USP8 can directly interact with specific proteins and regulate their ubiquitination. We performed co-IP/MS experiments, which showed that USP8 can directly bind to MUC12 (Fig. 6a, b). MUC12 belongs to the mucin (MUC) family, and we have intensively investigated the role of the Mucin family in tumor progression. TCGA database analysis suggested that high expression of MUC12 was closely related to high tumor grade and metastasis (Fig. 6c). We also found by western blotting that MUC12 was highly expressed in renal cancer tissues (Fig. 6d). Immunofluorescence staining and confocal microscopy revealed that USP8 and MUC12 were colocalized (Fig. 6e). The expression of MUC12 was markedly downregulated when USP8 expression was suppressed, as shown by western blotting (Fig. 6f). The results of western blotting after co-IP confirmed that USP8 could bind to MUC12 (Fig. 6g).

Furthermore, we used cycloheximide (CHX) to examine whether USP8 can regulate the stability of MUC12. After treating A498/786-O cells with 50 μg/L CHX, the MUC12 protein level was evaluated at various time points. After knockdown of USP8, degradation of the MUC12 protein was significantly accelerated, indicating that USP8 can affect the stability of the MUC12 protein (Fig. 6h). MG132 was used to treat A498/786-O cells that had been transfected with plasmids encoding HA-Myc and myc-ubiquitin plasmids encoding WT flag-USP8 or its mutant Flag-USP8-C786A, which is catalytically inactive. As shown in Fig. 6i, WT USP8 but not USP8-C786A significantly reduced the ubiquitination of MUC12. We also demonstrated that the autophagy lysosomal pathway did not significantly affect MUC12 expression by adding the autophagy inhibitor 3-MA (Supplementary Fig. 6i).

Moreover, the rescue experiments also suggested that in the A498 cell line with knockdown of piR-1742, overexpression of USP8 rescued the expression of MUC12. However, knockdown of USP8 in the piR-1742-overexpressing 786-O cell line reduced MUC12 expression (Fig. 6j). In addition, we measured MUC12-specific ubiquitination levels after knocking down or overexpressing piR-1742. We found that MUC12 ubiquitination was significantly increased after knocking down piR-1742. Additionally, the ubiquitination of MUC12 decreased after overexpressing piR-1742 (Supplementary Fig. 6j).

### MUC12 performs a carcinogenic function in RCC

In the next analysis, we examined whether MUC12 plays a fundamental role in the progression of RCC. GSEA based on the KEGG database showed that MUC12 expression was closely associated with pathways related to tumor development and progression, such as angiogenesis. (Supplementary Fig. 7a). MUC12 knockdown or overexpressing viruses were added to RCC organoids. Knockdown of MUC12 suppressed organoid proliferation and cell viability. However, overexpression of MUC12 promoted organoid proliferation and cell viability (Supplementary Fig. 7b).

Regarding in vitro experiments, the EdU incorporation assay showed that silencing MUC12 decreased the proliferative ability of A498 cells, whereas overexpression of MUC12 enhanced the proliferative capacity of 786-O cells (Supplementary Fig. 7c). Flow cytometry analysis revealed that silencing MUC12 increased the apoptotic capacity of RCC cells (Supplementary Fig. 7d). Angiogenesis assays revealed that the knockdown of MUC12 attenuated the tube-forming capacity of HUVECs (Supplementary Fig. 7e). Transwell assays demonstrated that silencing MUC12 prevented RCC cells from migrating and invading their surrounding environments. These results were validated in 786-O cells overexpressing MUC12 (Supplementary Fig. 7f). Rescue experiments confirmed that MUC12 affected migration, invasion and angiogenesis (Supplementary Fig. 7g).

Regarding in vivo experiments, a subcutaneous tumor model was established in mice, as shown in Supplementary Fig. 7h. The mice were divided into the control, MUC12 overexpression and MUC12 + piR-1742 antagomir overexpression groups. After tumors were allowed to for 15 days, the piR-1742 antagomir was injected into the mice every 4 days. The experimental results showed that overexpression of MUC12 significantly promoted tumor growth. However, injection of the piR-1742 antagomir into subcutaneous tumors overexpressing MUC12 inhibited tumor growth (Supplementary Fig. 7i–k).

### Liposomal PDA NPs targeting transmembrane MUC12 and encapsulating a piR-1742 inhibitor suppress RCC progression

**Fig. 7:**
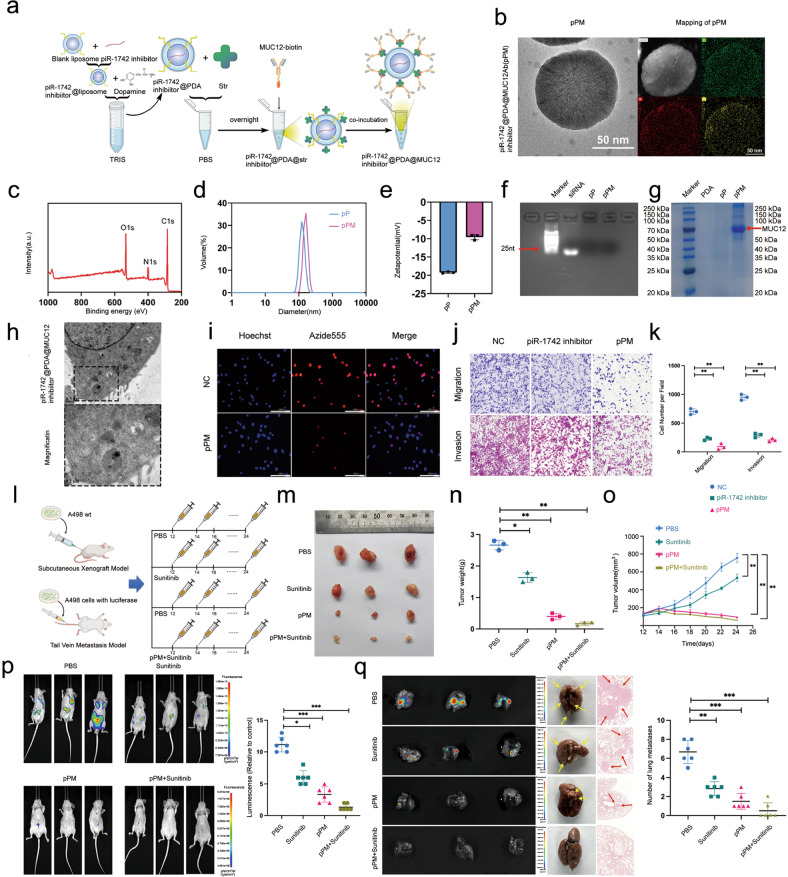
Liposomal PDA NPs targeting transmembrane MUC12 and encapsulating a piR-1742 inhibitor suppress RCC progression. **a** Schematic illustration of the process used for pPM-NP synthesis. **b** Transmission electron microscopy (TEM) image of pPM-NPs with the EDS map shown on the right. **c** XPS spectra showing C, N, and O in pPM-NPs. **d** Size distribution of pPM and pPM-NPs. **e** Zeta potential of pPM and pPM-NPs in water. **f** Results of gel electrophoresis for evaluation of RNA encapsulation in the three subgroups. **g** Results of Coomassie blue staining for evaluation of MUC12 incorporation on the surface of pPM-NPs. **h** Uptake of pPM-NPs, with a magnified image on the right. **i** Representative images of the EdU incorporation assay utilized to ascertain the proliferative capacity of A498 cells treated with pPM; Scale bars = 10 μm. **j**, **k** Transwell assays were utilized to analyze the migratory and invasive capacities of A498 cells treated with pPM; scale bars = 100 and 50 μm. **l** Graphical illustration of the nude mouse subcutaneous xenograft model and tail vein metastasis model, in which PBS/pPM/sunitinib were injected every 2 days. Analyses of tumor specimens (**m**), tumor weight (**n**) or tumor growth (**o**) in the mice bearing subcutaneous xenografts. **p**, **q** Representative images and quantification of bioluminescence in tail vein-injected nude mice and their lung tissue; the mice were implanted with A498 cells transfected with luciferase and treated using PBS/pPM/sunitinib. Data are presented as the mean ± SD of three experimental trials. *p < 0.05, **p < 0.01.

At present, liposomal and polydopamine nanoparticle (PDA-NP) drug delivery systems (NDDSs) are widely used in cancer therapy^19,20^. Based on our previous findings on piR-1742 and MUC12, we synthesized pPM-NPs for clinical therapy of RCC, and the strategy for preparing the pPM-NPs is shown in Fig. 7a. TEM showed that the synthesized pPM-NPs had a nearly spherical shape, and EDS mapping showed the C, N, and O elements, proving the successful synthesis of PDA (Fig. 7b). In addition, UV, Raman and FTIR spectroscopy confirmed the formation of the PDA coating (Supplementary Fig. 8a–c). The XPS spectra showed that the pPM-NPs contained C, N, and O (Fig. 7c, Supplementary Fig. 8d–f). The decreased zeta potential and increased diameter indicated that MUC12 was incorporated into the pPM surface and that the pPM-NPs had good stability, with an average diameter of approximately 140 nm (Fig. 7d, e). The results of agarose gel electrophoresis and colloidal Coomassie staining showed that the piR-1742 inhibitor was successfully encapsulated and that the pPM surface was modified with MUC12 (Fig. 7f, g). Phagocytosis experiments showed that pPM-NPs were successfully internalized into A498 cells for successful delivery (Fig. 7h). We also evaluated the metabolic distribution and safety of pPM-NPs (labeled with cy5.5) in different organs after tail vein injection by in vivo imaging and HE staining, respectively(Supplementary Fig. 8g, h).

Subsequently, the impacts of pPM-NPs on the biological behaviors of RCC cells were examined. On the basis of the findings of the EdU incorporation and CCK8 assays, pPM-NPs reduced the proliferation capacity of A498 cells (Fig. 7i, Supplementary Fig. 9a–c). A498 cells were tested for migration and invasion using a transwell assay, which revealed that pPM-NPs inhibited these behaviors (Fig. 7j, k, Supplementary Fig. 9d). Angiogenesis assays showed that pPM-NPs inhibited the tube formation capacity of cells (Supplementary Fig. 9e).

As shown in Fig. 7l, we also evaluated the effect of pPM-NPs on RCC growth and metastasis in vivo. Sunitinib is a drug that is prescribed as a first-line treatment for metastatic renal cancer. Therefore, we evaluated the synergistic effect of pPM-NPs and sunitinib on RCC proliferation and metastasis. The findings showed that pPM-NPs suppressed the growth of subcutaneous tumors and tumor metastasis, with the most significant effect in the pPM-NP plus sunitinib group (Fig. 7m–q).

**Fig. 8:**
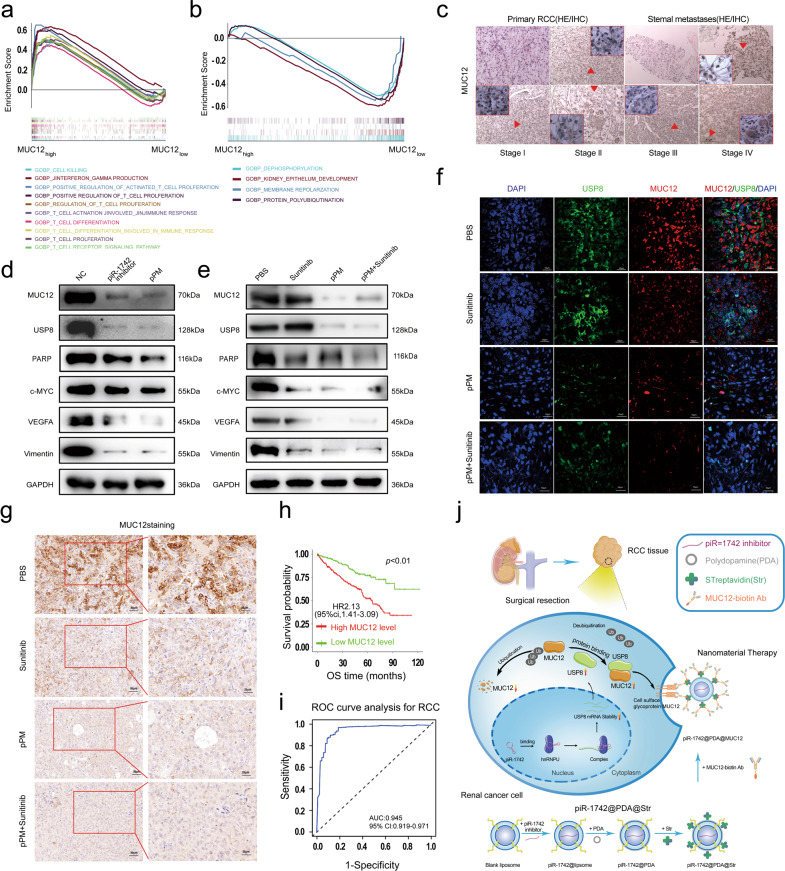
The piR-1742/USP8/MUC12 axis is critical for the malignant progression and metastasis of RCC. **a**, **b** The results of GSEA showed the gene sets highly enriched in the high and low MUC12 expression groups. **c** Representative images of IHC staining of MUC12 in samples of different stages, primary lesions and metastases. **d**, **e** Western blotting was utilized to examine the expression of MUC12, USP8, VEGFA, c-MYC, PARP and Vimentin. **f** Representative images of double immunofluorescence staining of USP8 and MUC12; scale bars = 10 μm. **g** Representative image IHC staining of MUC12 in the nude mouse subcutaneous tumor model; scale bars = 50 and 25 μm. **h** Kaplan‒Meier analysis of RCC patients with high and low expression levels of MUC12. **i** ROC curve of the piR1742-MUC12 signature for the differentiation of RCC tissues from normal tissues. **j** The proposed model of the mechanism by which piR-1742 promotes the malignant progression of RCC by stabilizing USP8 mRNA and inhibiting MUC12 ubiquitination. Data are presented as the mean ± SD of three experimental tests. *p < 0.05, **p < 0.01.

In view of the important roles of piR-1742 and MUC12 in RCC cells, the enriched pathways positively and negatively related to MUC12 were analyzed via GSEA based on GOBPs. MUC12 expression was markedly positively linked to cell death, IFN-γ, and the tumor microenvironment but significantly negatively correlated with the growth and differentiation of normal kidneys (Fig. 8a, b). The expression of MUC12 was further analyzed in RCC samples of different grades, primary RCC lesions and sternal metastases. IHC staining indicated that the expression of MUC12 increased significantly with increasing malignancy of RCC. In addition, the expression of MUC12 in metastatic samples was significantly higher (Fig. 8c). Simultaneously, western blot analysis of subcutaneous tumor tissues further indicated that treatment with pPM-NPs in combination with sunitinib exhibited the most significant inhibitory effect on the malignant progression of RCC cells (Fig. 8d, e). Immunofluorescence and IHC staining of subcutaneous tumor tissues showed that treatment with pPM-NPs in combination with sunitinib significantly inhibited the expression of MUC12 (Fig. 8f, g). In contrast with patients exhibiting lowered MUC12 expression levels, those with elevated expression levels had substantially poorer OS outcomes (Fig. 8h).

We then developed a piR-1742-MUC12 signature based on the RNA expression of piR-1742 and MUC12. The ROC curve showed that the piR-1742-MUC12 signature markedly discriminated RCC from normal tissues, with an AUC of 0.945 (Fig. 8i). piR-1742 and MUC12 are independent risk factors for predicting OS in RCC patients, based on data from our hospital (Supplementary Table 2). Both multivariate and univariate Cox regression analyses further confirmed that the piR-1742_MUC12 signature was an independent prognostic indicator for RCC (Supplementary Fig. 10a). A nomogram based on the piR-1742_MUC12 signature and other clinicopathological features was developed to predict survival outcomes in RCC patients (Supplementary Fig. 10b). The calibration plots showed that the survival outcomes predicted by the nomogram were similar to the actual 1-, 3- and 5-year OS outcomes (Supplementary Fig. 10c–e).

## Discussion

Renal cancer is among the top ten most common malignancies in the United States, with RCC accounting for 90% of cases^21^. Therefore, identifying and understanding the molecular pathways involved in the tumorigenesis and progression of RCC has significant implications for both fundamental science and therapeutic applications. In recent years, the molecular processes through which piRNAs contribute to carcinogenesis have been discovered in a variety of tumor types^22–24^. In this work, we identified a piRNA that could be used as a biological marker for the onset and progression of RCC. Our data add a biological marker, piRNA-1742, to the catalog of prognostic factors for RCC and reveal its roles in RCC metastasis and progression on targeted therapy.

A relatively limited amount of research has been conducted on piRNAs. At present, it is obvious that piRNAs have biological functions in the suppression of transposon mobilization by triggering transcript disintegration and modulating chromatin synthesis^25^. In particular, piRNAs might produce piRNA-PIWI protein complexes with certain components of the PIWI branch of the argonaute protein family^26^. piRNAs and PIWI proteins are critical for preserving the integrity of the genome in germline cells^27^. Therefore, research into piRNAs has concentrated on their function in germline cells^28^. As research in this field continues to advance, accumulating evidence has indicated that piRNAs are aberrantly expressed in a variety of malignancies, implying that they might serve as tumor biological markers or cancer treatment targets^29,30^. In this work, we employed a microarray to identify piRNAs differentially expressed between mRCC tissues and corresponding normal tissues. We were particularly interested in the function and underlying processes of enhanced piR-1742 expression in the progression of RCC. Utilizing a variety of bioinformatics techniques, we were able to identify and confirm putative RCC-specific piRNAs in several RCC cohorts. Mass spectrometry and RNA pull-down assays showed that piR-1742 can directly bind to the hnRNPU protein to form a complex.

To the best of our knowledge, this is the first work to elucidate that abundantly expressed piR-1742 might act as a critical mediator in the activation of USP8/MUC12 signaling, hence enhancing the proliferation of RCC cells while also promoting RCC invasion and metastasis. Experiments in xenograft-bearing mice and organoid models revealed that treatment with a piR-1742 inhibitor (antagomir-1742) effectively suppressed RCC growth and metastasis. Our recent investigations showed that the expression of piR-1742 in RCC samples was strongly linked to clinical outcomes. Altogether, these observations offer additional valuable insights into the critical roles played by some piRNAs in the onset and progression of human cancer, and they highlight the great promise for the use of such piRNAs as therapeutic targets as well as useful diagnostic and prognostic biological markers in cancer.

Recent research has shown that deubiquitination and ubiquitination perform integral functions in metabolic reprogramming in cancer cells^31,32^. As a multistep enzyme-mediated process, ubiquitination is among the most significant types of posttranslational modifications and is implicated in a wide range of biological processes^33,34^. Multiple disorders, including cancer, are caused by the dysregulation of the deubiquitination and ubiquitination processes^31,35^. In this study, we clarified an association between modified piRNA-1742 and abnormal deubiquitination in RCC. The introduction of piRNA-1742 mimics led to markedly increased USP8 mRNA expression, leading to a considerable increase in deubiquitination^36^. Following additional investigation, it was discovered that piRNA-1742 directly bound to hnRNPU and increased USP8 stability. In addition, the FISH results showed that piR-1742 and USP8 were significantly colocalized in the cytoplasm. This recently discovered activity of piR-1742 in RCC cells might serve as an inspiration for future research into the functions of additional piRNAs in other kinds of human cancers. There have been several research reports demonstrating that USP8 is a carcinogen that has antiapoptotic and prometastatic impacts^37^. In particular, we discovered that piR-1742 substantially enhances the expression and/or activity of USP8 downstream pathways, including the prometastatic VEGFA and antiapoptotic c-MYC pathways. These results supplement information already available about the biological activities of piRNAs and their critical involvement in the pathogenesis and maintenance of human cancers.

Furthermore, we examined the significance of alterations in deubiquitination in biological behaviors of RCC and investigated the processes underlying the involvement of USP8 in these behaviors. MUC12 was identified as a key target gene of USP8 by Co-IP/MS, which supported our findings. Overexpression of USP8 increased the deubiquitination level of MUC12, leading to an increase in the MUC12 protein level. Investigations on sunitinib-based targeted therapies have offered convincing evidence for the substantial contributions of MUC12 to renal cancer occurrence and indicate that MUC12 is a critical metastatic promoter of the RCC phenotype. Our hypothesis derived from the findings of the aforementioned investigations is that the piRNA-1742/USP8/MUC12 axis may perform a vital function in the onset and progression of RCC.

Members of the mucin family are glycoproteins and are mainly localized in the cell membrane^38,39^. Available research evidence suggests that transmembrane mucins may be involved in the loss of polarity in epithelial cells^40^. Therefore, as observed in clinical malignancies, upregulation of transmembrane mucins might enhance the malignant EMT phenotype by altering polarity and cell‒cell interactions^41^. MUC12, a transmembrane mucin containing an SEA module, three EGF-like sequences, and C-terminal tandem repeats, has a domain structure similar to that of MUC1^42,43^. However, as one of the mucin family members, MUC12 is rarely investigated, especially in RCC.

MUC12 extracellular domains are frequently shed in overabundance in cystic fibrosis, inflammatory bowel disease, and metastatic carcinomas. Tang et al. noted that MUC12 could increase the protein level of c‐Jun, which transcriptionally regulates TGF‐β1^44^. These results indicated that increased MUC12 expression is a frequent event supporting the growth of malignancy and inflammation. In this research, the MUC12 expression level was shown to be elevated in larger xenograft tumor samples, whereas the expression of MUC12 was inhibited when piR-1742 was knocked down. Notably, USP8 knockdown suppressed MUC12 expression, and the promoting effects of MUC12 on angiogenesis were blocked by silencing of piR-1742. Moreover, elevated expression of MUC12 in RCC was found to be considerably linked to higher TNM stage, larger tumor size, and unfavorable prognosis, confirming the finding that piR-1742 overexpression is correlated with higher TNM stage and larger tumor size.

As a consequence of these findings, it was postulated that piR-1742/USP8/MUC12 played a significant role in the regulation of MUC12-mediated tumor invasiveness via a ‘one-hit/multiple targets’ process. Subsequently, we synthesized NPs incorporating piR-1742/siRNA against the transmembrane metastasis marker MUC12. Functional assays verified that treatment with the NPs significantly reduced the expression of piR-1742 and MUC12, which in turn suppressed the progression and metastasis of RCC. Although further investigations are required to thoroughly examine the possible impacts of piR-1742 suppression, the findings of the current study clearly show that piR-1742 could be a therapeutic target for the successful treatment of RCC.

In summary, utilizing in vitro and in vivo data, this work demonstrates that piR-1742 promotes disease progression and leads to a dismal prognosis in patients with RCC by modulating the deubiquitination of key molecules, consequently activating downstream signaling cascades. In addition, this work shows the functional significance of the deubiquitination process in RCC and offers important insights into the epigenetic processes triggering carcinogenesis by revealing a previously unknown method of gene modulation in RCC. The inhibition of RCC cell growth and metastasis in mice following therapy with a piR-1742 antagonist demonstrates that piR-1742 could be a potential treatment target in RCC. Because of the functional significance of piR-1742 in the onset and progression of RCC, targeting the piR-1742/USP8/MUC12 axis with selective nanomaterial carriers may constitute an interesting therapeutic option for the treatment of RCC (Fig. 8j).

## Supplementary information

Supplemental files

Supplementary Table S3

Supplementary Table S4

Supplementary Table S5

Supplementary Table S6

Supplementary Table S7

Full Length Uncropped Original Western Blots

## Data Availability

The Supplementary Materials and Methods include further in-depth explanations of the methods. The data generated in this study are available within the article and its Supplementary data files (Supplementary Methods, Supplementary Tables 4, 5).
