# Supplementary material for: piRNA-1742 promotes renal cell carcinoma malignancy by regulating USP8 stability through binding to hnRNPU and thereby inhibiting MUC12 ubiquitination
Source: Exp Mol Med. 2023 Jun 19;55(6):1258–71. doi: 10.1038/s12276-023-01010-3 (PMC10318070; doi:10.1038/s12276-023-01010-3)
Supplement: Supplementary file 1 — Supplemental files [file 12276_2023_1010_MOESM1_ESM.pdf]

piRNA-1742 promotes renal cell carcinoma malignancy by regulating USP8 stability through binding to hnRNPU and thereby inhibiting MUC12 ubiquitination

Wentao Zhang[1,2#], Zongtai Zheng[3#], Keyi Wang[1,2#], Weipu Mao[1,2,4#], Xue Li[5], Guangchun Wang[1,2], Yuanyuan Zhang[6], Jianhua Huang[1,2], Ning Zhang[7], Pengfei Wu[1,2,] Ji Liu[1,2], Haimin Zhang[1,2], Jianping Che[1,2], Bo Peng[1,2*], Junhua Zheng[8,9*] , Wei Li[1,2,7*], Xudong Yao[1,2*]

Supplemental Methods

RNA sequencing

For piRNA-sequencing, 3 pairs of renal cancer patient tissues were used to identify differentially expressed piRNAs. The total RNA from each sample was quantified. For microarray analysis, the labeled samples were hybridized onto Arraystar Human piRNA Array in Agilent's SureHyb Hybridization Chambers (Agilent Technologies) [20]. After having washed the slides, the arrays were scanned by the Agilent Scanner G2505C. Agilent Feature Extraction software (version 11.0.1.1) was used to analyze acquired array images. Quantile normalization and subsequent data processing were performed using the GeneSpring GX v11.5.1 software. Significant differentially expressed genes between the two groups were identified through Volcano Plot filtering. Finally, Hierarchical Clustering was performed to show the distinguishable piRNAs expression pattern among samples. A |fold change (FC)| > 1.5 and a p<0.05 were used as cutoff values.

For RNA-seq, USP8 knockdown or overexpress cells and negative controls were collected and total RNA was extracted. RNA-seq was performed on Illumina HiSeq2000, data were aligned to the UCSC human hg19 genome using STAR 2.5. "DESeq2" and "Cluster

Profiler" were used to analyze RNA-seq data. A log|FC| > 1 and false discovery rate (FDR) < 0.05 were used as cutoff values.

Western blot and Co-IP

Renal cell lines or tissues were extracted to total protein, and the protein concentration of lysates was quantified by BCA assay. For western blotting, 40 μg protein samples were separated by electrophoresis on sodium dodecyl sulfate-polyacrylamide gels and transferred to NC membranes (Sigma-Aldrich; Merck KGaA). Membranes were then blocked with 5% non-fat milk for 1 hour at room temperature and incubated with primary antibody overnight at 4°C. The results were visualized and quantified by Tanon 5200 system (Tanon, Shanghai, China). For Co-IP, lysates of 1×10^7 RCC cells were immunoprecipitated with IP buffer containing IP antibody-coupled argarose beads, and protein-protein complexes were used to western blot. The results were visualized and quantified by Tanon 5200 system (Tanon, Shanghai, China). IgG was used as a negative control.

CCK8 assay and Cloney Formation

Cell proliferation was detected by CCK-8 assay (Yeasen, shanghai, china). 2×10^3 cells/well were seeded into 96-well plates.10 μl of CCK-8 reagent was added to each well and incubated at 37°C for 1 hour. Absorbance at 450 nm was measured using a microplate spectrophotometer (BioTek, Winooski, VT). For cloney formation, 1×10^3/well was seeded into 6-well plates and the plates were cultured for 14 days at 37°C with 5% CO_2. Then, the plates were, fixed with 75% ethanol after cold PBS washed 3 times, and 0.1% crystal violet was used for stained cells.

RNA isolation and qRT-PCR

Total RNA was extracted from human RCC tissues or cell lines, according to the manufacturer's instructionso. Then, the RNA was then reverse transcribed into the first

strand cDNA using the stem-loop method by a specific stem-loop reverse transcription kit (Vazyme Biotech Co., Ltd, China). SYBR qPCR kit (Vazyme, China) and ABI Prism 7500 sequence detection system (Applied Biosystems, CA) were used for qRT-PCR. piR-1742 primer designed and synthesized by RiboBio (PQPSCM001-2, RiboBio Biotechnology, Guangzhou, China). The qPCR parameters are as follows: 40 cycles of 30 seconds at 95°C, then 10 seconds at 95°C, and 30 seconds at 60°C. U6/GAPDH was used as an endogenous control. The relative fold change is analyzed by the $2^{-\Delta\Delta C(t)}$ method.

Fractionation of nuclear and cytoplasmic RNA

To detect the subcellular localization of piRNAs, a PARIS kit (Thermo Fisher, MA, USA, Cat#AM1921) was used for the extraction of nuclear and cytoplasmic RNAs following the manufacturer's protocol. The qPCR was used to detect RNA abundance in different cell fractions. U6 and GAPDH were used as internal controls for the nucleus and cytoplasm, respectively.

Immunofluorescence

RCC cells were seeded onto cell slides. Cells were fixed in 4% formaldehyde for 15 min and permeabilized with Triton X-100 (0.1%) for 20 min. After blocking with goat serum for 30 minutes, the primary antibody was added and incubated overnight at 4°C. The next day, cells were incubated with a secondary antibody for 1 hour at room temperature, and DAPI was used for nuclei staining. A confocal microscope (Zeiss, Pleasanton, CA, USA) was used to capture the images.

Northern Blot

Total RNA was extracted, and 15% urea denaturing PAGE gel was used for total RNA electrophoresis. RNA was immobilized with Stratagene UV cross-linker after about 1h at a constant voltage of 60V. According to the instructions of the Northern Blot Assay Kit, the piR-1742 RNA probe modified with locked nucleic acid and digoxigenin-labeled was

used for hybridization reaction with the membrane, and the U6 probe was used as the internal reference. Bands are exposed and analyzed after using HRP Anti-Digoxigenin.

RNA immunoprecipitation

The RIP assays were performed using a RIP kit (BersinBio, Guangzhou, China). According to the manufacturer's instructions. 1×10^7 RCC cells were immunoprecipitated with RIP buffer containing hnRNPU antibody-coupled magnetic beads, and RNA was extracted from RNA-protein complexes. Then, the expression of piR-1742 was verified by qPCR. IgG was used as a negative control.

RNA pull-down

The piR-1742-binding proteins were examined using an RNA pull-down kit (BersinBio, Guangzhou, China). Biotin-modified piR-1742 and NC probes were incubated with protein extract overnight, followed by precipitation with streptavidin magnetic beads. The retrieved protein was eluted from the RNA-protein complex. Afterward, western blot or mass spectrometry was performed (Oebiotech, shanghai, China).

EdU assay

3×10^4 cells were seeded on cell slides. The next day, the EdU reagent (Yeasen Biotechnology, shanghai, China) was added to the cells and incubated for 1 hour. Cells were fixed in 4% paraformaldehyde for 15 minutes and then permeabilized by adding Triton X-100 (0.3%) for 15 minutes. Add 500 µl of EdU reaction mix (containing Azide 555) to cells and incubate in the dark for 30 min. Hoechst was used for nuclear staining. Images were captured under a fluorescence microscope.

IHC

Human RCC tissue or mouse tumor tissue was fixed in 4% paraformaldehyde, followed by paraffin embedding and sectioning. For IHC, tissue sections were deparaffinized, dehydrated, antigen retrieved, and blocked. After blocking, the tissue was incubated with

antibodies overnight at 4°C. The next day, tissues were incubated with biotinylated goat anti-rabbit IgG for 20 minutes at room temperature, followed by streptavidin-horseradish peroxidase for 30 minutes. Finally, staining was performed using diaminobenzidine-H_2O_2 and hematoxylin.

Dual-luciferase reporter assays

The dual-luciferase reporter assays were performed to detect the epigenetic regulation of PiR-1742 and USP8. Bioinformatics analysis was performed using the miRanda algorithm. The wild-type (WT) and mutant (Mut) pGL3 plasmids were constructed and transfected to 293T. After 48 hours, cells were lysed to detect firefly and Renilla fluorescence by a dual-luciferase reporter assay system, and statistical analysis was performed.

Fluorescence in situ hybridization (FISH) and RNA In Situ Hybridization (RNA ISH)

RCC cells were seeded on cell slides, after which cells were fixed in 4% paraformaldehyde and 0.5% Triton X-100. Subsequently, cy3-labeled piR-1742 and NC probes (Zima, Shanghai, China) were added and hybridized overnight at 37°C. DAPI was used for nuclei staining and images were captured by confocal microscopy (Carl Zeiss AG, Germany) for further analysis. The double (5' and 3')-digoxigenin (DIG)-labeled probe targeting piR-1742 was designed and synthesized by RNAscope®. Expression of piR-1742 was detected using the RNAscope 2.0 detection kit (Red, Cat No. 324500) according to the manufacturer's instructions. The piR-1742 expression was calculated as follows: H-score=Σpi（i+1）, pi represents the percentage of the number of positive cells; i represents the intensity of staining.

Nude mouse xenograft assay

1×10^7 RCC cells were injected subcutaneously into the right armpit of a 5-week-old female BALB/c nude mouse. 5-week-old female BALB/c nude mice were subcutaneously

injected with 1×10^7 RCC cells. When tumors were ~100 mm^3 (10 days), the mice were randomly divided into two groups. One was intraperitoneally injected with antagomir piR-1742 (16 mg/kg), and the other groups were injected with PBS. After 4 weeks, the mouse was sacrificed, and the tumors were removed for IHC, IF, or western blot. The mouse weight and tumor diameter/volume were monitored every 3 days according to the formula (tumor volume = $\pi/6\times length\times width^2$).

Tail vein metastasis in nude mouse

1×10^6 RCC cells in 100 μL of PBS were injected into the tail vein of the mouse to observe metastasis in the nude mouse. After 4 weeks, tumor metastasis in nude mice was detected by *in vivo* imaging system (IVIS) every 3 days. Finally, the nude mouse was sacrificed, and the lung tissues were removed to detect tumor metastasis by IVIS. Lung tissue was used for HE and IHC experiments.

TCGA database and Gene Set Enrichment Analysis (GSEA)

GSEA was used to obtain different biological pathways between groups (http://www.broadinstitute.org/gsea/index.jsp). A nominal $p<0.05$ and a false discovery rate <0.25 were considered significant. The mRNA (RNA-sequence) Fragments Per Kilobase of transcript per Million Fragments standardized expression data set along with clinical information were obtained from The Cancer Genome Atlas (TCGA)-RCC cohort (https://portal.gdc.cancer.gov).

Synthesis of piR-1742-inhibitor@PDA@MUC12 nanoparticles

The nano-delivery systems are designed for the targeted therapy of RCC. We developed piR-1742-inhibitor@PDA@MUC12 nanoparticles (pPM-NPs) through a simple and green method. Firstly, 40 nM piR-1742-inhibitor was dissolved in the commercial liposomes (100 μL; Yeasen, China) through a 20s vortex. And the obtained solution was subsequently added to the tris-HCl (pH 8.8; 10 mM) with a final volume of 5 ml.

Meanwhile, 5 mg dopamine hydrochloride was incorporated in the above solution to form polydopamine (PDA) modified liposomes under stirring for 3 h. The PDA-modified liposomes were concentrated by 8,000 rpm centrifugation (15 min) and washed with RNase-free water. Next, the resulting mixture was dissolved in 1 ml streptavidin solution (2 mg/mL; dissolved in PBS with the pH 8~9) and shaken for 12 h in the dark under 4℃ for the preparation of piR-1742-inhibitor@PDA@str(pP). Finally, the prepared pP was added to 1 ml biotinylated MUC12 antibody solution (50 μg/mL; Bioss, China) and shaken for 1 h in the dark under 4℃ to generate the pPM-NPs. The obtained pPM-NPs suspension was washed with distilled water and stored in a PBS buffer under 4℃.

Characterization of nanoparticles

Transmission electron microscope (TEM) imaging was established to characterize SPM-NPs. The samples were immersed in water and dropped on the carbon-cover copper TEM grids. After preparation, the sample was photographed by TEM (JEOL, Tokyo, Japan). Ultraviolet-visible (UV-vis) absorption spectra of pPM-NPs were examined by UV-vis spectrophotometer. Fourier Transform infrared spectroscopy (FTIR) and X-ray photoelectron spectroscopy (XPS) were conducted to evaluate the synthesis of PDA by FTIR-8300 series spectrometer (Shimadzu, Japan) and ESCALAB 250 Xi Mg (Thermo Scientific, Japan) X-ray resource. Meanwhile, the Raman spectroscopy of pPM was examined by using a WITEC Spectra Pro 2300I spectrometer. Additionally, the scanning transmission electron microscopy (STEM) of pPM-NPs was also measured, which was accompanied by energy dispersive X-ray spectrometry (EDS) element mapping examination. The diameters of pP and pPM-NPs were measured using a particle size potentiometer (Nano ZS90, Worcestershire, UK); and the Zeta potential was also simultaneously examined.

Encapsulation capability of siRNA

The gel electrophoresis was conducted to examine the encapsulation capability of siRNA possessed by pPM-NPs. There were three subgroups including siRNA as control, pP, and pPM. 0.5 g agarose was added into 40 ml 1×TAE buffer to form the gels through the boil and cool process. And the RNA or NPs were mixed with 2 μl 6×DNA loading buffer and 1 μl SYBR Green I nucleic acid gel stain solution to gain the 12 μl final mixture. Then the mixture was added to the dented pores with 80 V electrophoresis voltage for 30 min. After the electrophoresis, the gel was imaged using the gel imaging system and photographed by the Tanon Gel image system (Shanghai, China).

MUC12 incorporation measurement

Western blot analysis was conducted to examine the incorporation of MUC12 antibody with the contribution of coomassie blue staining. The experimental subgroups were divided into PDA, pP, and pPM. 16 μl samples were mixed with 4 μl 5× protein loading buffer and heated at 100℃ for 30 mins. And the mixtures were separately added in the dented pores of the 10% sodium dodecyl sulfate-polyacrylamide gels (SDS-PAGE) for electrophoresis with 120 V. After 1.5 h electrophoresis, the gel was dyed by coomassie blue staining buffer for 3 h and washed by distilled water subsequently. The final gel was photographed the next day.

Supplemental Figures

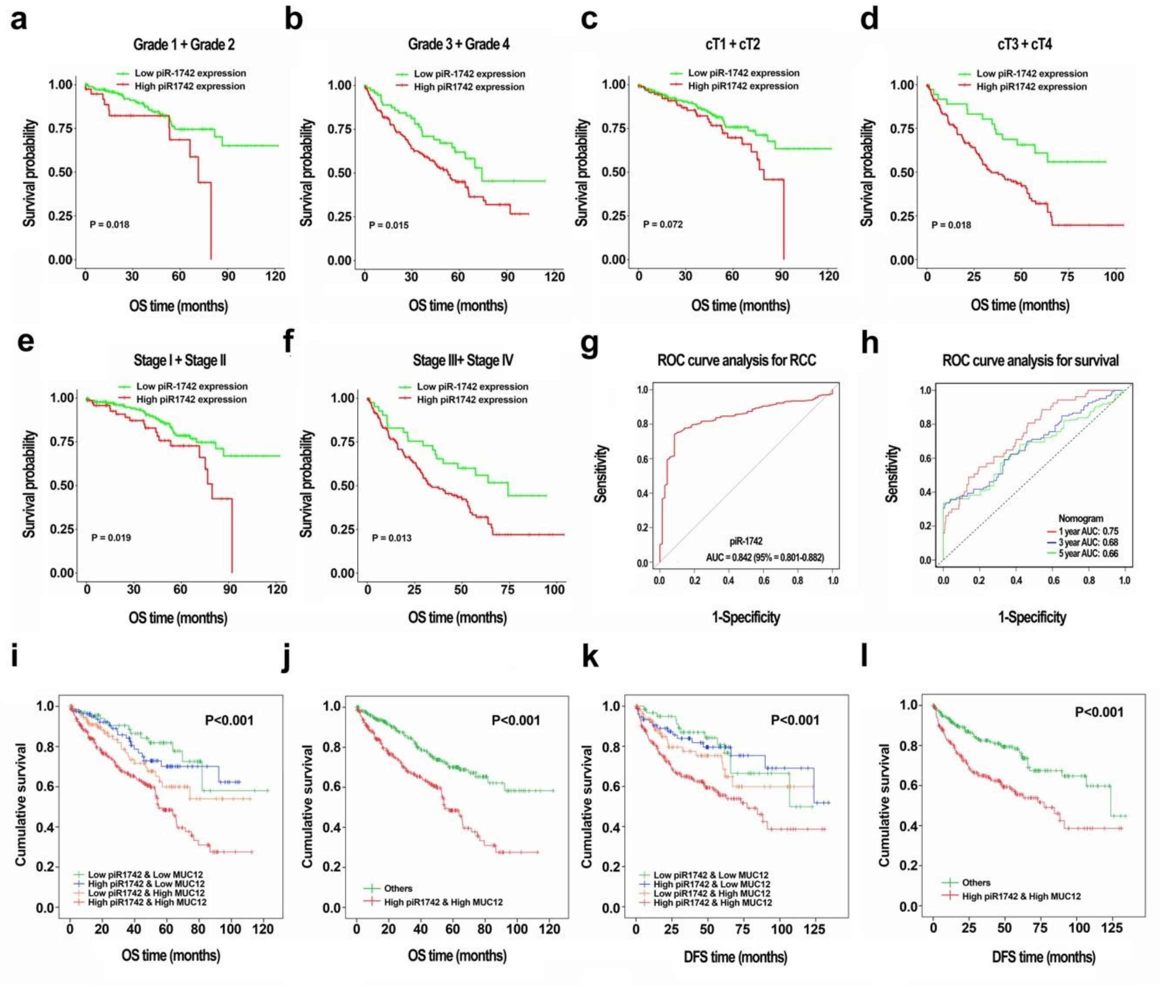

Supplementary Figure 1 The performance of piR-1742 and nomogram in the TCGA cohort. Kaplan-Meier analysis of the piR1742 in samples with different tumour grades and stages: tumour grade (**a, b**), clinical T stage (**c, d**), and tumour stage (**e, f**). **g** The ROC curve of piR-1742 for the differentiation of RCC tissues from normal tissues. **h** ROC curves demonstrating the efficiency of the nomogram predicting 1-, 3-, and 5-year survival rates. (**i, j**) and DFS analysis (**k, l**) of patients with RCC based on the expression patterns of piR-1742 and MUC12. *p < 0.05, **p < 0.01.

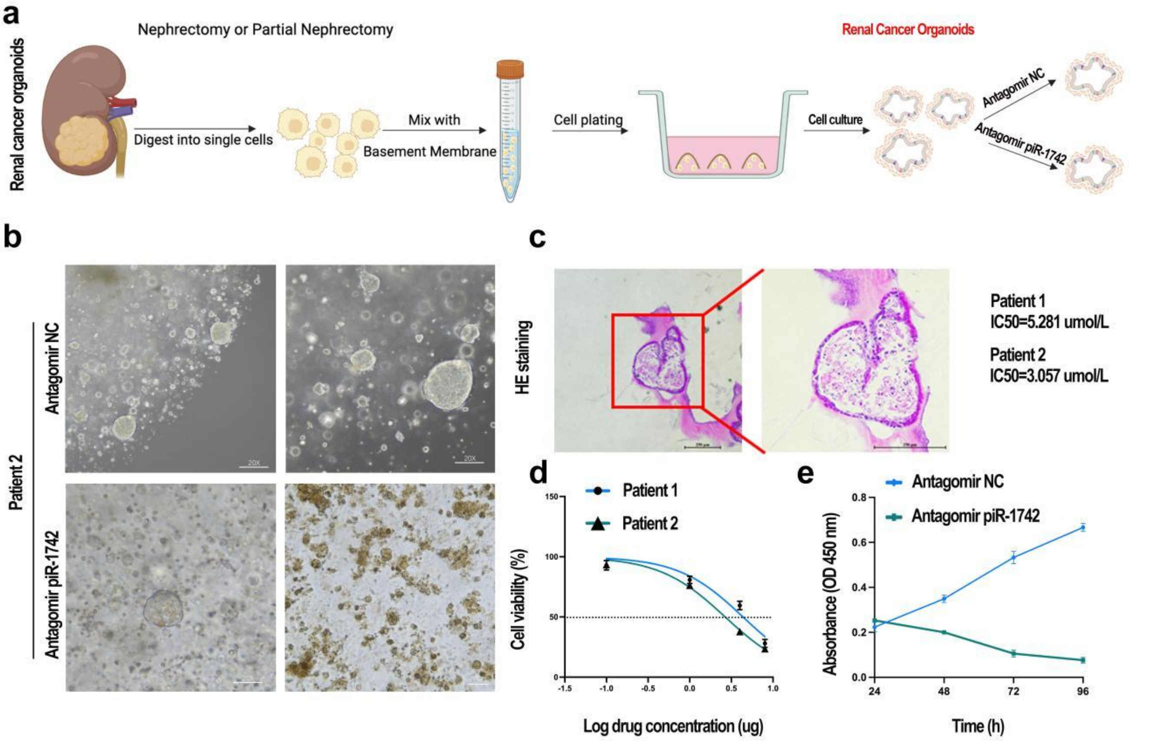

Supplementary Figure 2 piR-1742 antagomir inhibits renal cancer organoid proliferation **a** Graphic illustration of RCC organoids. **b** Representative images of piR-1742 antagomir affecting organoid; scale bars = 100/50 μm. **c** HE staining of the constructed RCC organoids; scale bars = 100/50 μm. **d** IC50 values of piR-1742 antagomir in tissue cells from two patients with RCC. **e** CCK8 assay was used to detect the cell viability of organoids after being treated with piR-1742 antagomir; scale bars = 100/50 μm. Data indicate mean ± SD of three experiments. *$p < 0.05$, **$p < 0.01$.

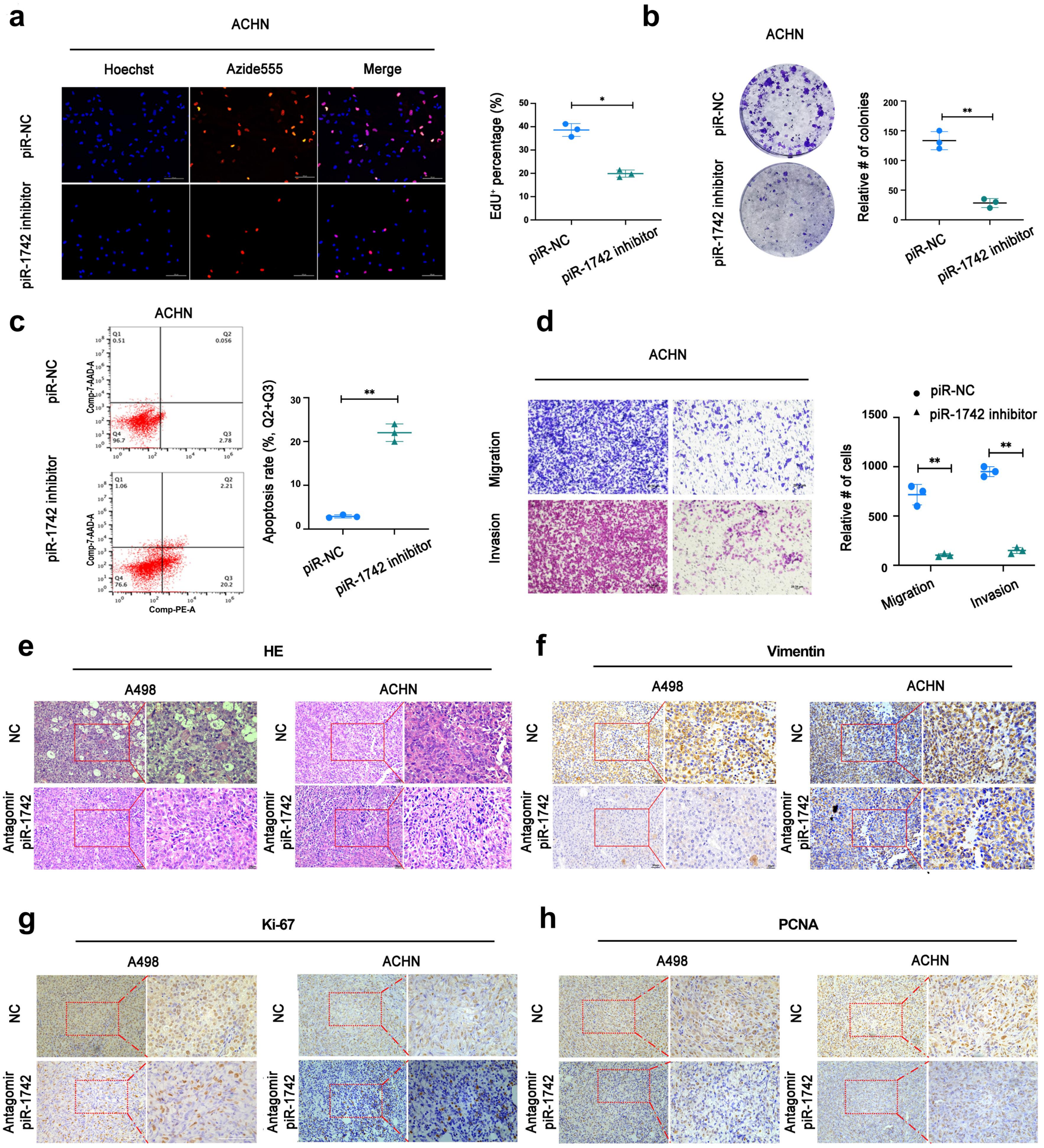

Supplementary Figure 3 piR-1742 promotes the malignant progression of RCC cells. **a** Representative images of EdU assay used to measure the proliferation of ACHN cells treated with piR-1742 inhibitor; Scale bars = 10 μm. **b** Representative images of clone formation assay used to measure the proliferation of ACHN cells treated with piR-1742 inhibitor. **c** Flow cytometry analysis was used to detect the effects of piR-1742 on the apoptosis of ACHN cells. **d** Transwell assay was used to analyse the migration and

invasion abilities of ACHN cells after piR-1742 knockdown; scale bar = 100/50 μm. **e** Representative images of HE staining of subcutaneous tumour tissues in nude mice; scale bars = 100/50 μm. **f-h** Representative images of IHC staining for Vimentin, Ki-67, and ACHN in subcutaneous tumour tissues of a nude mouse. Data indicate mean ± SD of three experiments. *$p < 0.05$, **$p < 0.01$.

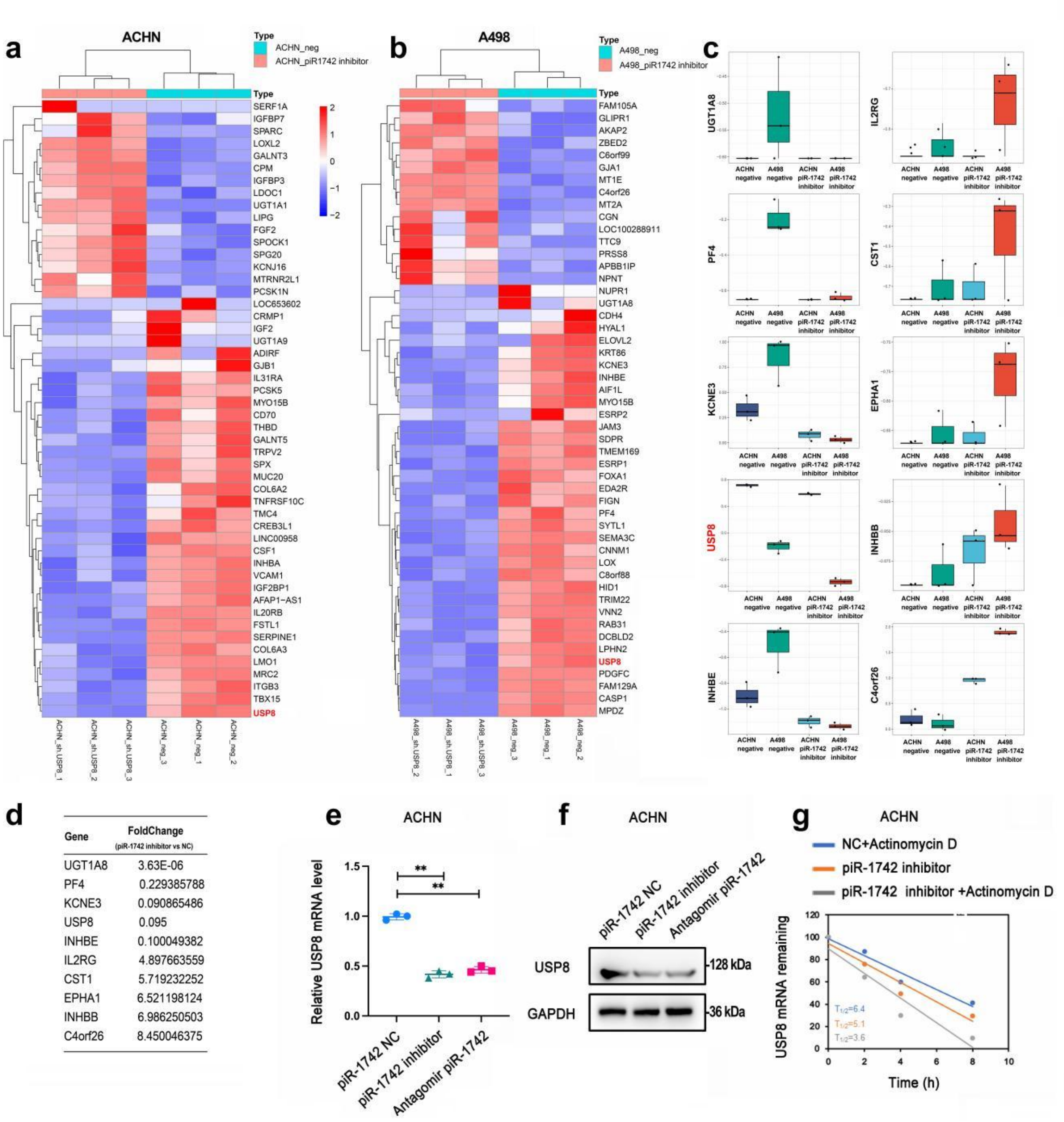

Supplementary Figure 4 The results of RNA-sequence in ACHN and A498 cell lines. Heatmaps demonstrating the top 50 DEGs in ACHN (**a**) and A498 (**b**) cell lines. **c** The

expression patterns and fold changes of the top 5 up-regulated DEGs and top 5 down-regulated DEGs between piR-1742 inhibitor and negative control. **d** Top 5 up-regulated DEGs and top 5 down-regulated DEGs. **e** qPCR was used to detect USP8 mRNA after piR-1742 knockdown in ACHN cells. **f** Western blot was used to detect USP8 protein expression after piR-1742 knockdown in ACHN cells. **g** qPCR was used to measure the half-life (t1/2) of USP8 mRNA in ACHN cells.*$p < 0.05$, **$p < 0.01$.

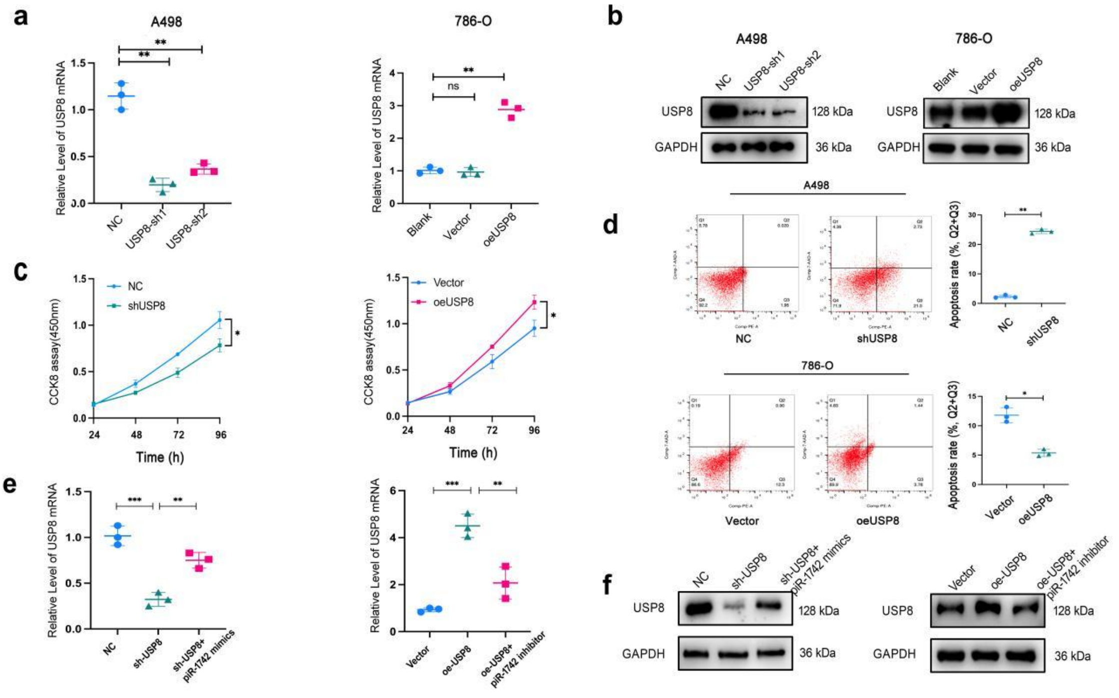

Supplementary Figure 5 The role of USP8 in renal cancer progression. a-b qPCR and western blot were used to validate the efficiency of USP8 knockdown and overexpression. **c** CCK 8 assay was used to detect changes in the proliferation of A498/786-O cells after USP8 knockdown or overexpression. **d** Flow cytometry analysis was used to detect the effect of USP8 on the apoptosis of A498/786-O cells. **e-f** After adding piR-1742 inhibitor or mimics to cell lines that knockdown or overexpress USP8, the expression of USP8 was detected by qPCR and western blot. Data indicate mean ± SD of three experiments. *$p < 0.05$, **$p < 0.01$.

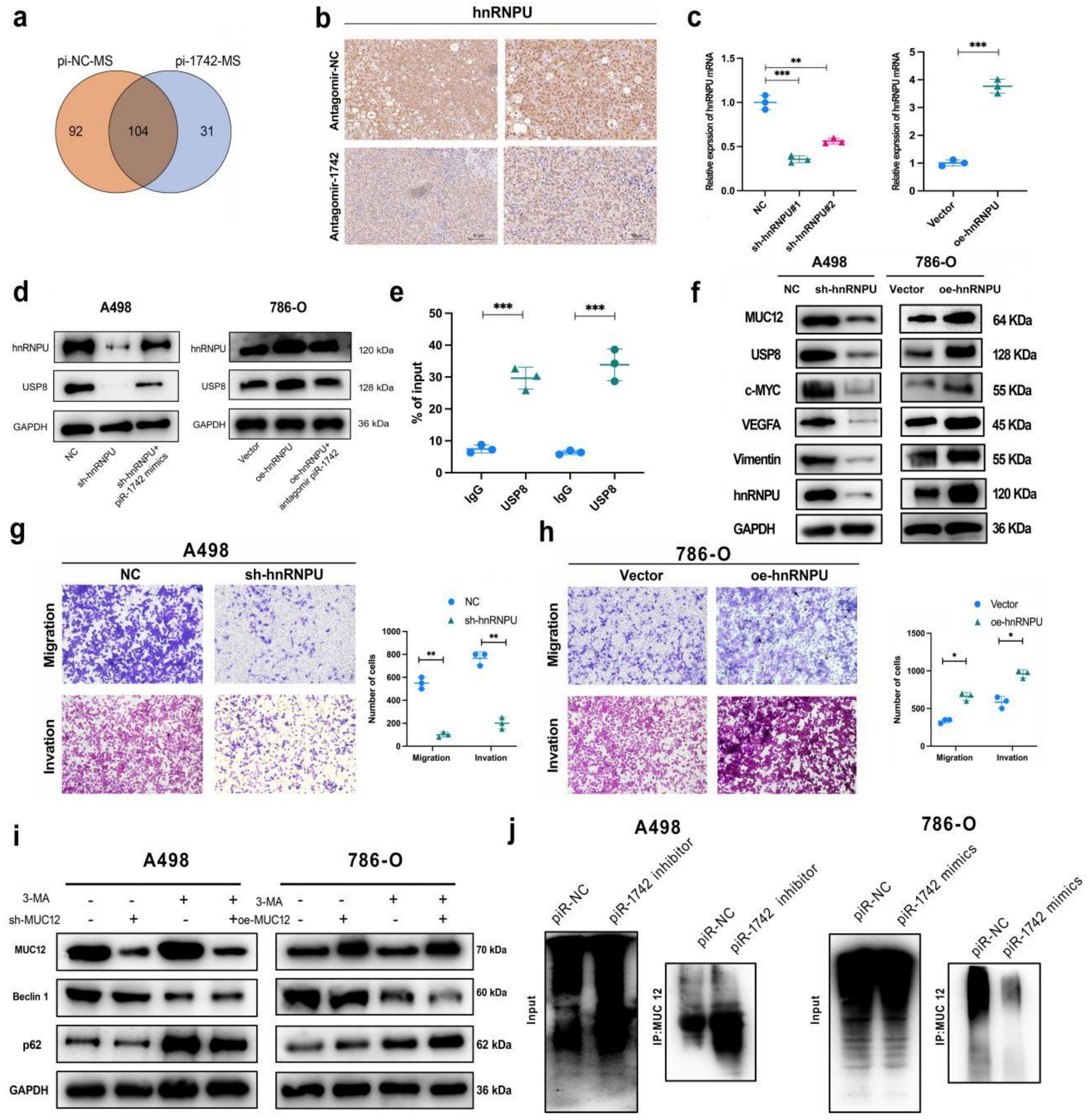

Supplementary Figure 6 piR-1742 directly interacts with hnRNPU. a 31 differential binding proteins were screened by Venn diagram after RNA pull-down and Mass spectrometry analysis. **b** IHC staining of hnRNPU in subcutaneous tumours of a nude mouse treated with piR-1742 antagomir. **c** qPCR was used to verify the efficiency of knocking down or overexpressing hnRNPU.**d** Rescue experiment to verify the expression of USP8 and hnRNPU in A498 and 786-O by western blot. **e** RIP experiments confirm that hnRNPU can directly bind to USP8 mRNA. **f** Western blot was used to determine the expression of MUC12, USP8, VEGFA, c- MYC and EMT-related makers. **g-h** Transwell

assay was used to analyse the migration and invasion abilities of A498/786-O cells after hnRNPU knockdown or overexpression; scale bar = 100/50 μm. **i** Effects of using the autophagy inhibitor 3-MA on MUC12 expression after knockdown or overexpressing MUC12. **j** The expression of MUC12-specific ubiquitination after knocking down or overexpressing piR-1742 by western blot. Data indicate mean ± SD of three experiments. **p* < 0.05, ***p* < 0.01.

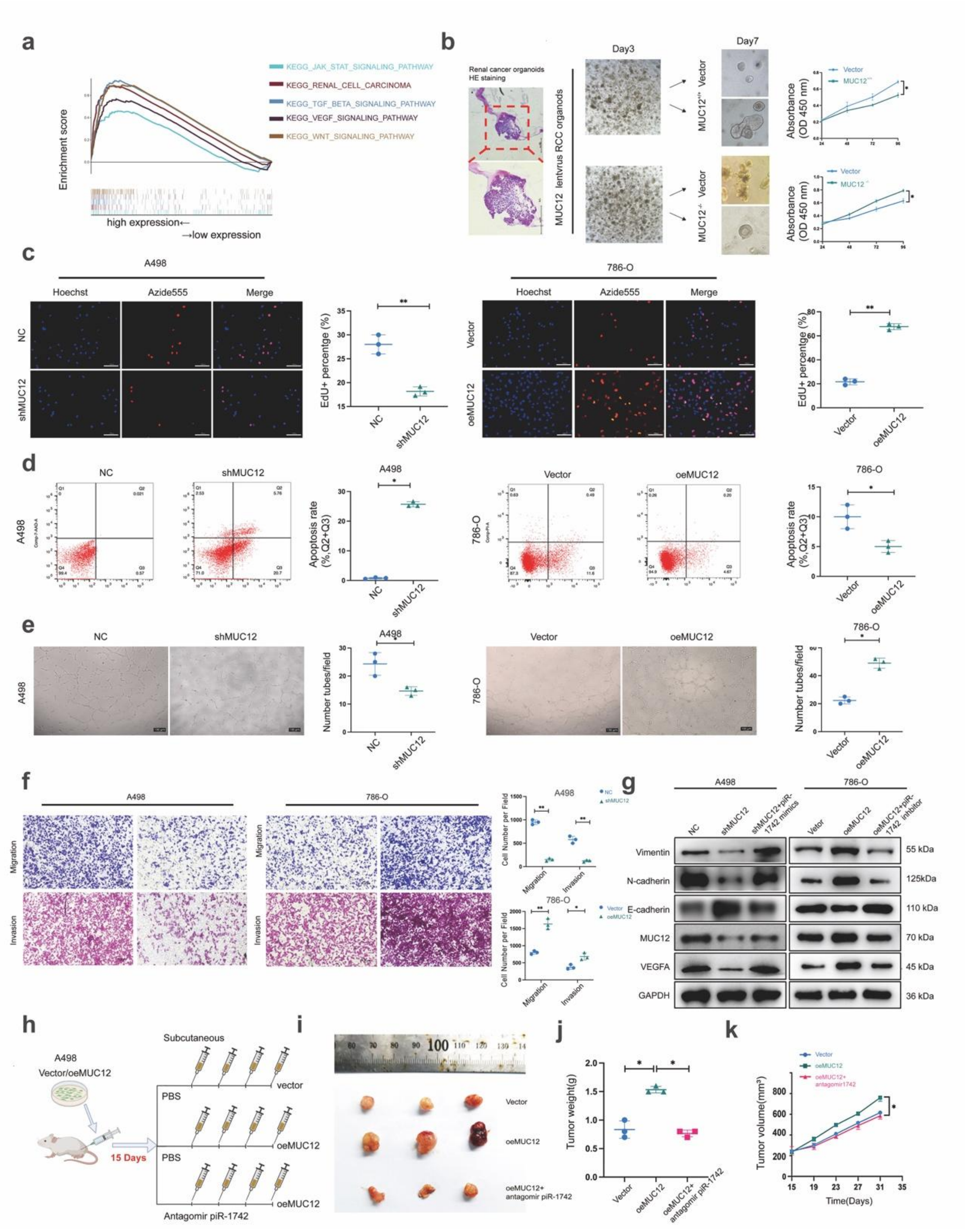

Supplementary Figure 7 MUC12 performs an oncogenic function in RCC. a The results of GSEA were highly enriched in the high MUC12 expression group. **b** Representative images of MUC12 knockdown or overexpressing virus infecting organoids and CCK8 assay to assess their cell viability; scale bar = 100/50 μm. **c** Representative images of EdU assay, which was used to measure the proliferation of MUC12-knockdown or MUC12-overexpressing RCC cells; scale bar = 10 μm. **d** Flow cytometry was utilized to evaluate the impact of MUC12 on RCC cell apoptosis. **e** The impact of RCC cells knocking down or overexpressing MUC12 on the tube-forming ability of HUVEC cells.**f** Transwell assay was utilized to examine the migratory and invasive capacities of RCC cells after MUC12 overexpression or silencing; scale bar = 100/50 μm. **g** Western blot was implemented to evaluate the expressions of MUC12, VEGFA, and EMT-associated makers. **h** Graphic illustration of a nude mouse subcutaneous tumor model injected with PBS/ piR-1742 antagomir and the analyses of tumor specimens (**i**), weight (**j**), or tumor growth (**k**) in the subcutaneous xenotransplanted tumor models. Data are presented as mean ± SD of three separate experimental tests. $*p < 0.05$, $**p < 0.01$.

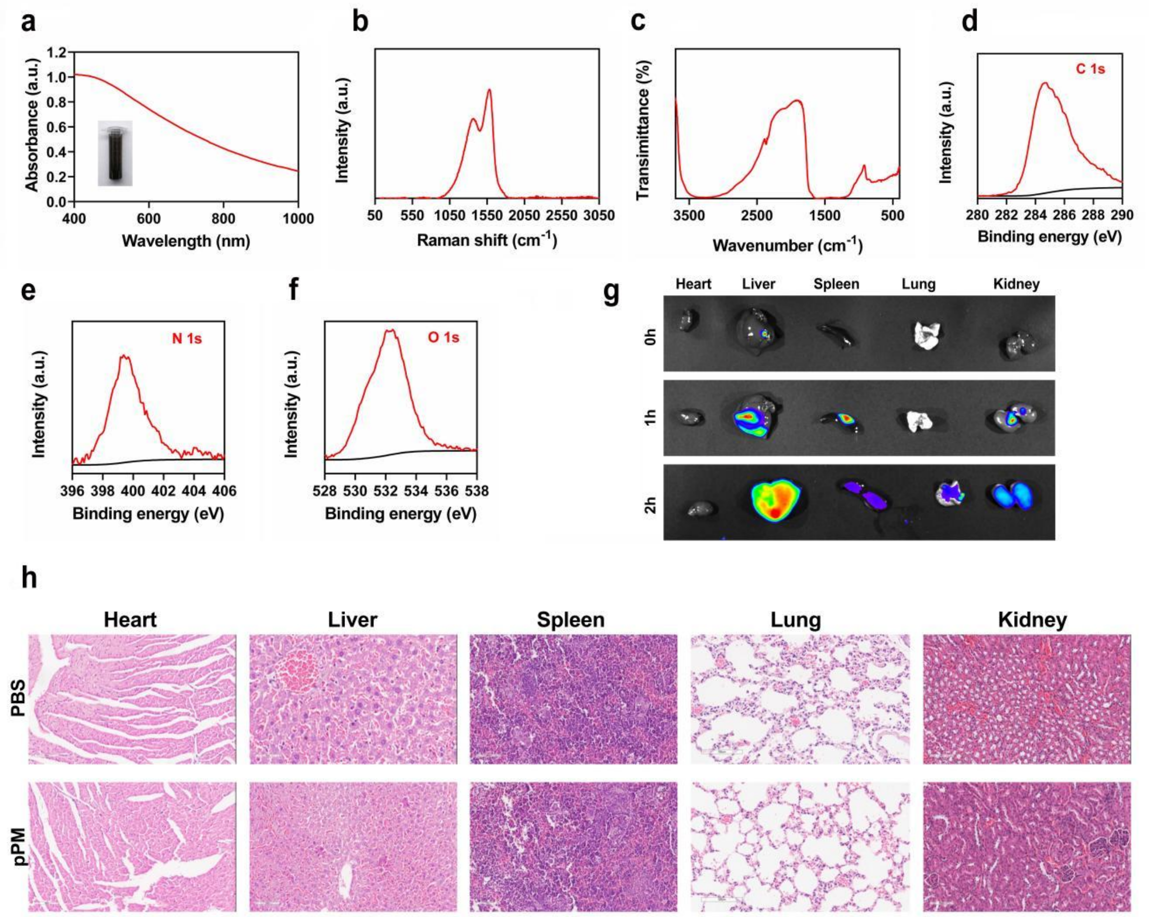

Supplementary Figure 8 Characterization of pPM-NPs. a Absorption spectrum of pPM-NPs dispersed in water. **b** Raman spectroscopy of pPM-NPs. **c** FTIR spectrum of pPM-NPs. (**d**) C 1s peak, (**e**) N 1s peak, and (**f**) O 1s peak in the XPS spectrum of pPM-NPs.**g** Distribution of pPM-NPs in different organs of mice by tail vein injection. **h** Representative images of HE staining of pPM-NPs and control in different organs of mice.

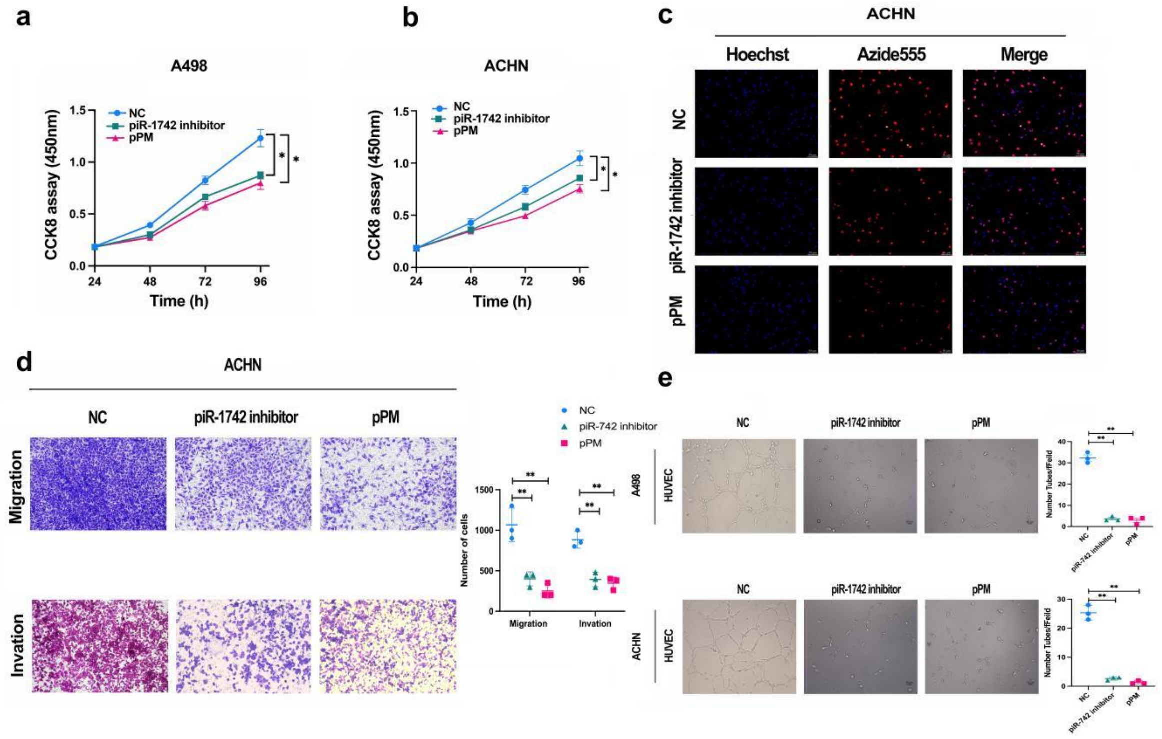

Supplementary Figure 9 Effects of pPM-NPs on renal cancer cell lines a-b CCK8 assay was used to detect the effect of piR-1742 inhibitor and pPM on cell proliferation. **c** Representative images of EdU assay which were used to measure cell proliferation after piR-1742 knockdown or after treatment with pPM; scale bar = 10 μm. **d** Transwell assay was used to analyse the migration and invasion abilities of ACHN cells after piR-1742 knockdown or after treatment with pPM; scale bar = 100/50 μm. **e** The effect of MUC12 knockdown or overexpression on the tube-forming ability of HUVEC cells. Scale bars = 100/50 μm. Data indicate mean ± SD of three experiments. *$p < 0.05$, **$p < 0.01$.

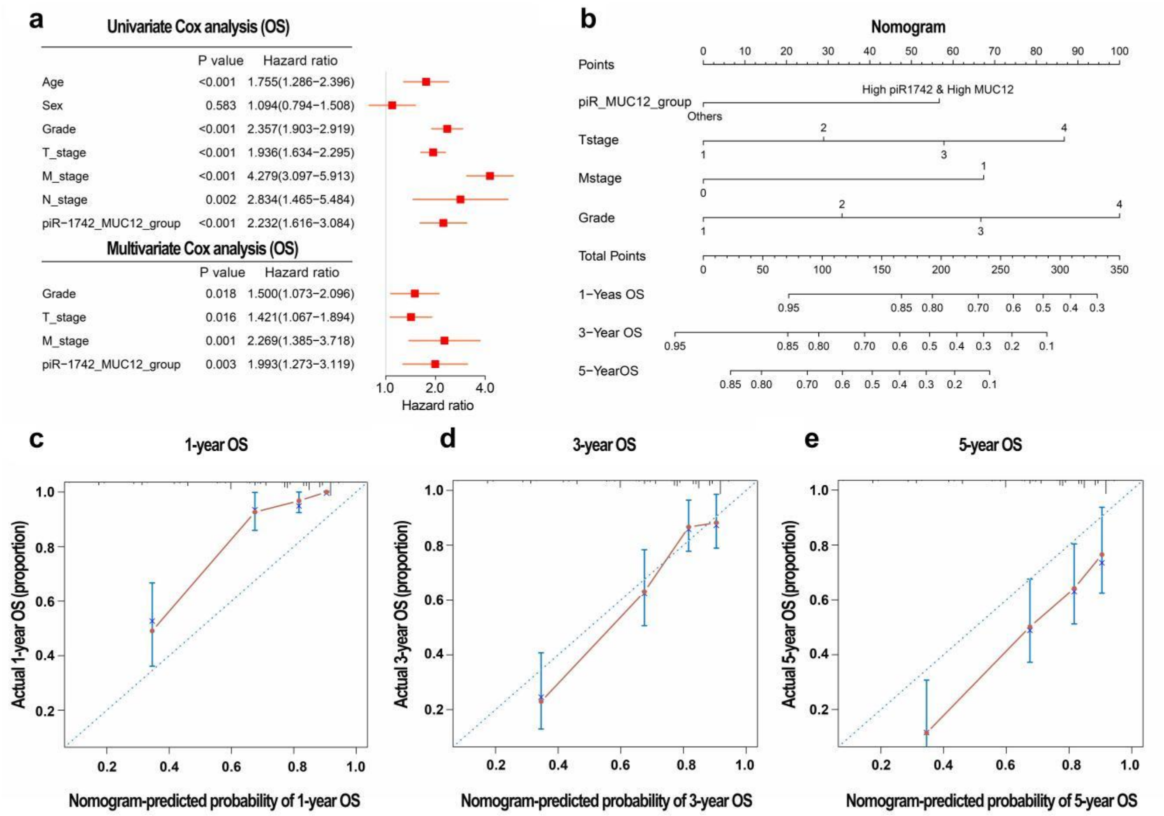

Supplementary Figure 10 Performance of the piR-1742-MUC12 signature. a Univariate and multivariate Cox regression of the piR-1742-MUC12 signature and other clinicopathological features. **b** A nomogram based on the piR-1742-MUC12 signature for predicting the probability of 1-, 3-, and 5-year OS. **c-e** Calibration plots of the nomogram for predicting the probability of 1-, 3-, and 5-year OS. Kaplan-Meier OS.

Supplemental Table 1 Characteristics of patients with ccRCC and associations with clinicopathologic variables

Variable	Total (%)	Variable	Total (%)
**Gender**		**Fuhrman grade**	
Male	76 (79.2)	1-2	53 (55.2)
Female	20 (20.8)	3	32 (33.3)
**Age at surgery (years)**		4	11 (11.5)
≤45	10 (10.4)	**AJCC clinical stage**	
＞45，≤55	57 (59.4)	I	35 (36.5)
＞55	29 (30.2)	II	25 (26.0)
**BMI**		III	22 (22.9)
＜25	53 (55.2)	IV	14 (14.6)
≥25	43 (44.8)	**Metastatic status**	
**Maximum tumor size (cm)**		NM	58 (60.4)
≤4	21 (21.9)	LM	26 (27.1)
＞4，≤7	37 (38.5)	DM	12 (12.5)
＞7，≤10	29 (30.2)	**T stage**	
＞10	9 (9.4)	T1	35 (35.4)
**Renal capsule invasion**		T2	27 (28.1)
No	58 (60.4)	T3	21 (21.9)
Yes	38 (39.6)	T4	1314.6)

Supplemental Table 2 Prognostic factors for OS in RCC patients

Variables (No.)	Univariate		Multivariate	
	P	OR	*P*	OR (95% CI)
**Gender**	0.732	0.845		
Male (76)				
Female (20)				
**Age at surgery**	0.800	1.108		
≤55 (67)				
＞55 (29)				
**Maximum tumor size**	0.722	1.146		
≤7 (58)				
＞7 (38)				
**Tumor metastasis**	0.001*	3.794	0.072	2.278(0.929-5.587)
Non-metastasis (58)				
Metastasis (38)				
**Fuhrman grade**	0.001*	4.839	0.033*	2.691 (1.081-6.695)
1-2 (53)				
3-4 (43)				
**pT Stage**	0.001*	4.812	0.403	1.494 (0.583-3.827)
T1-T2 (62)				
T3-T4 (34)				
**piR-1742 expression**	0.007*	15.444	0.028*	9.577 (1.272-71.825)
Low (30)				
High (66)				
**MUC12 expression**	0.005*	3.411	0.033*	2.567 (1.078-6.114)
Low (45)				
High (51)				

*Statistically significant.
