# Supplementary material for: piRNA-1742 promotes renal cell carcinoma malignancy by regulating USP8 stability through binding to hnRNPU and thereby inhibiting MUC12 ubiquitination
Source: Exp Mol Med. 2023 Jun 19;55(6):1258–71. doi: 10.1038/s12276-023-01010-3 (PMC10318070; doi:10.1038/s12276-023-01010-3)

Figure2 North Blot

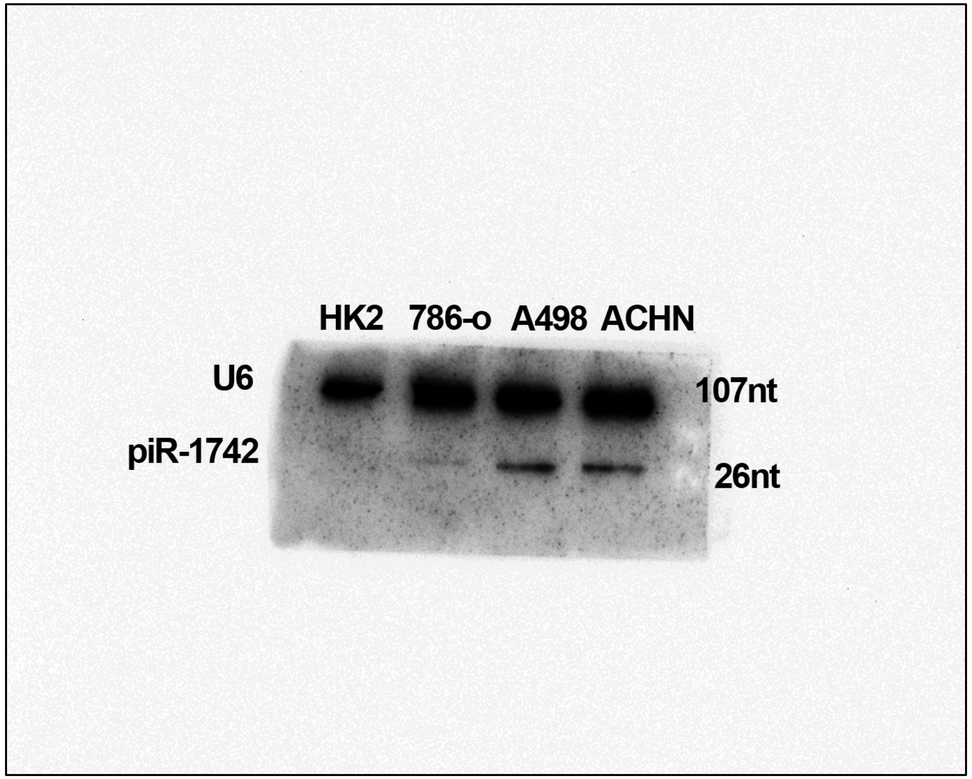

Figure4 C

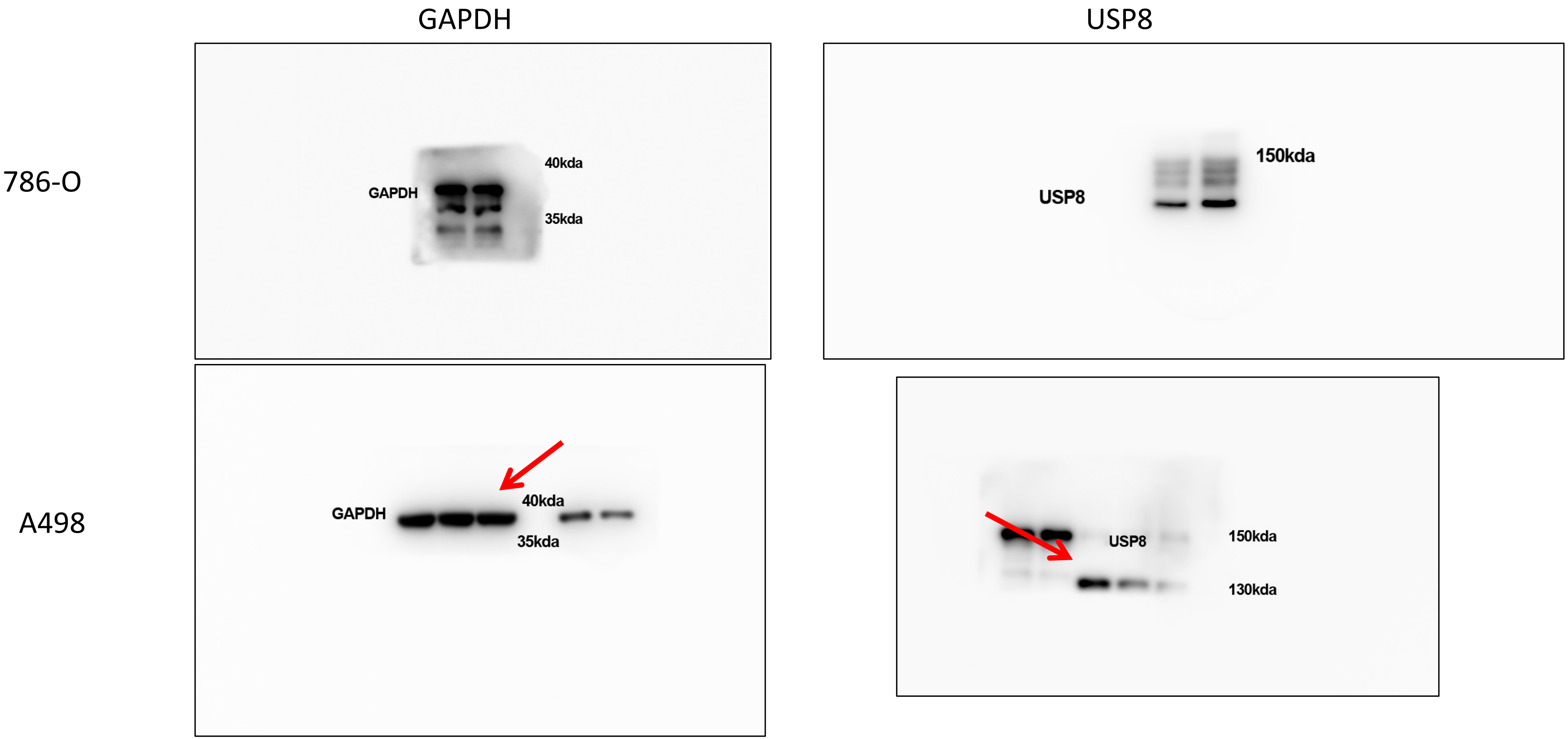
GAPDH
USP8
786-O
A498
GAPDH
40kda
35kda
USP8
150kda
GAPDH
40kda
35kda
USP8
150kda
130kda

figure 5i

hnRNPU GAPDH

786-O

A498

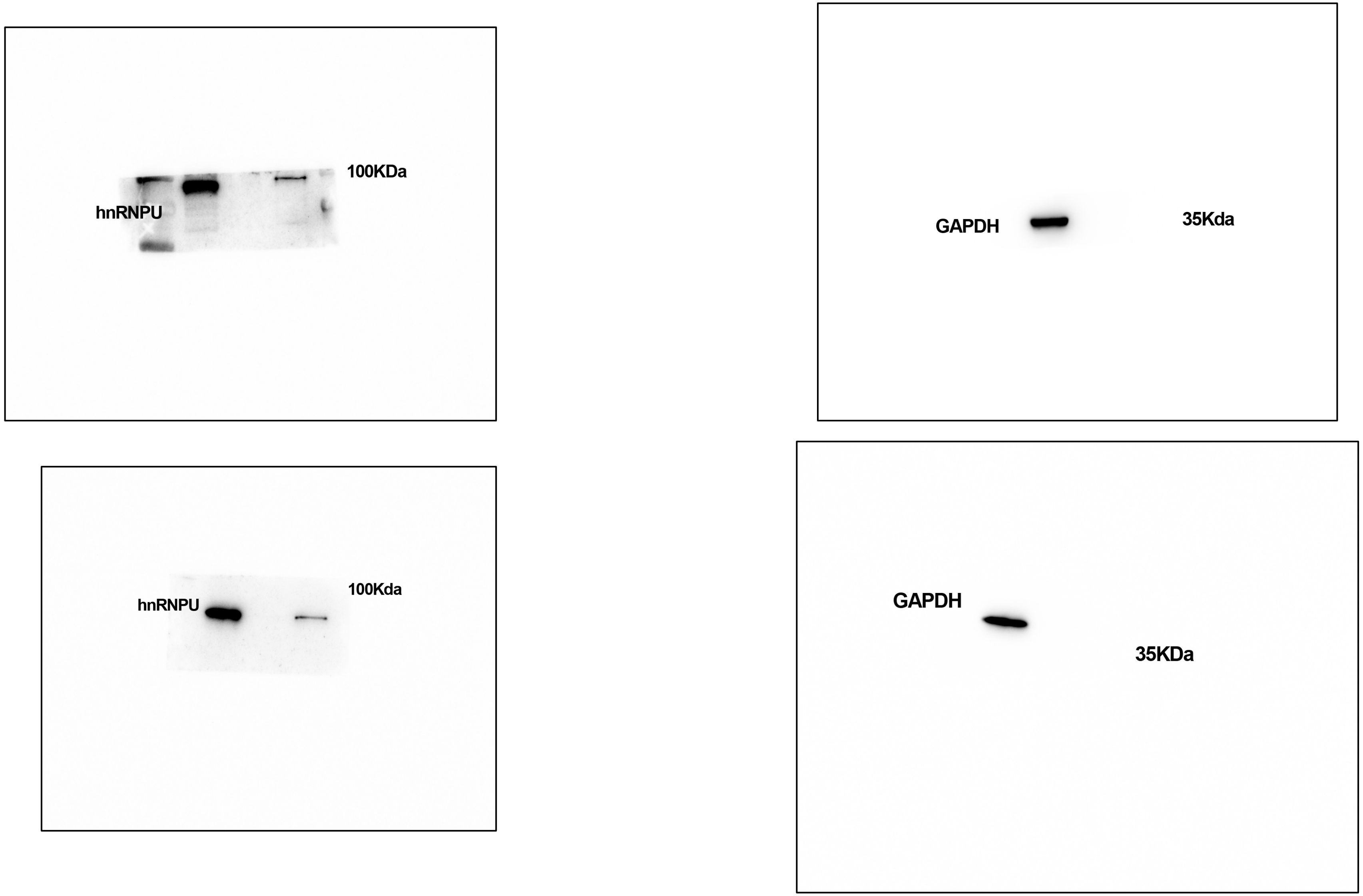

figure 5J and K

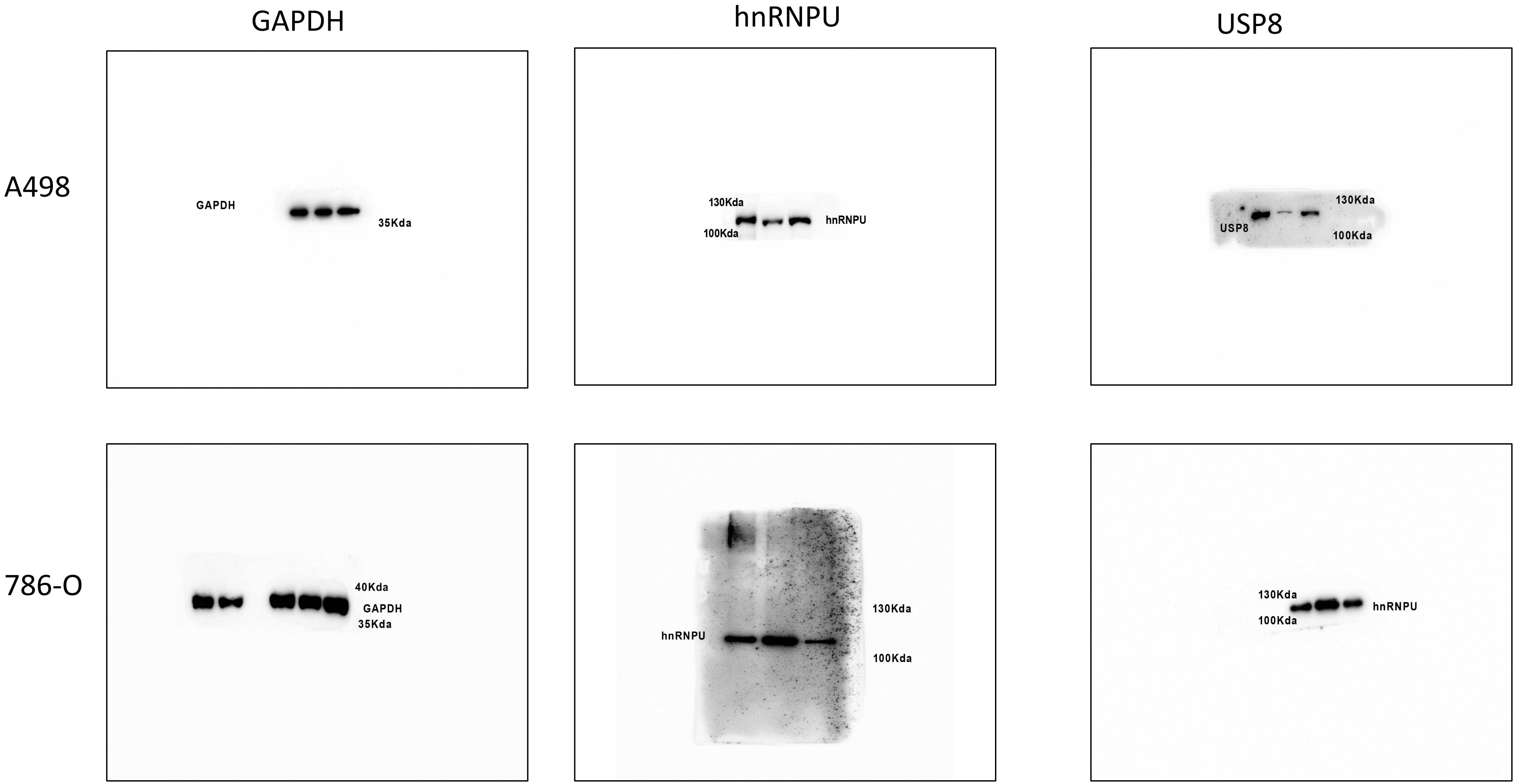

Figure6 d

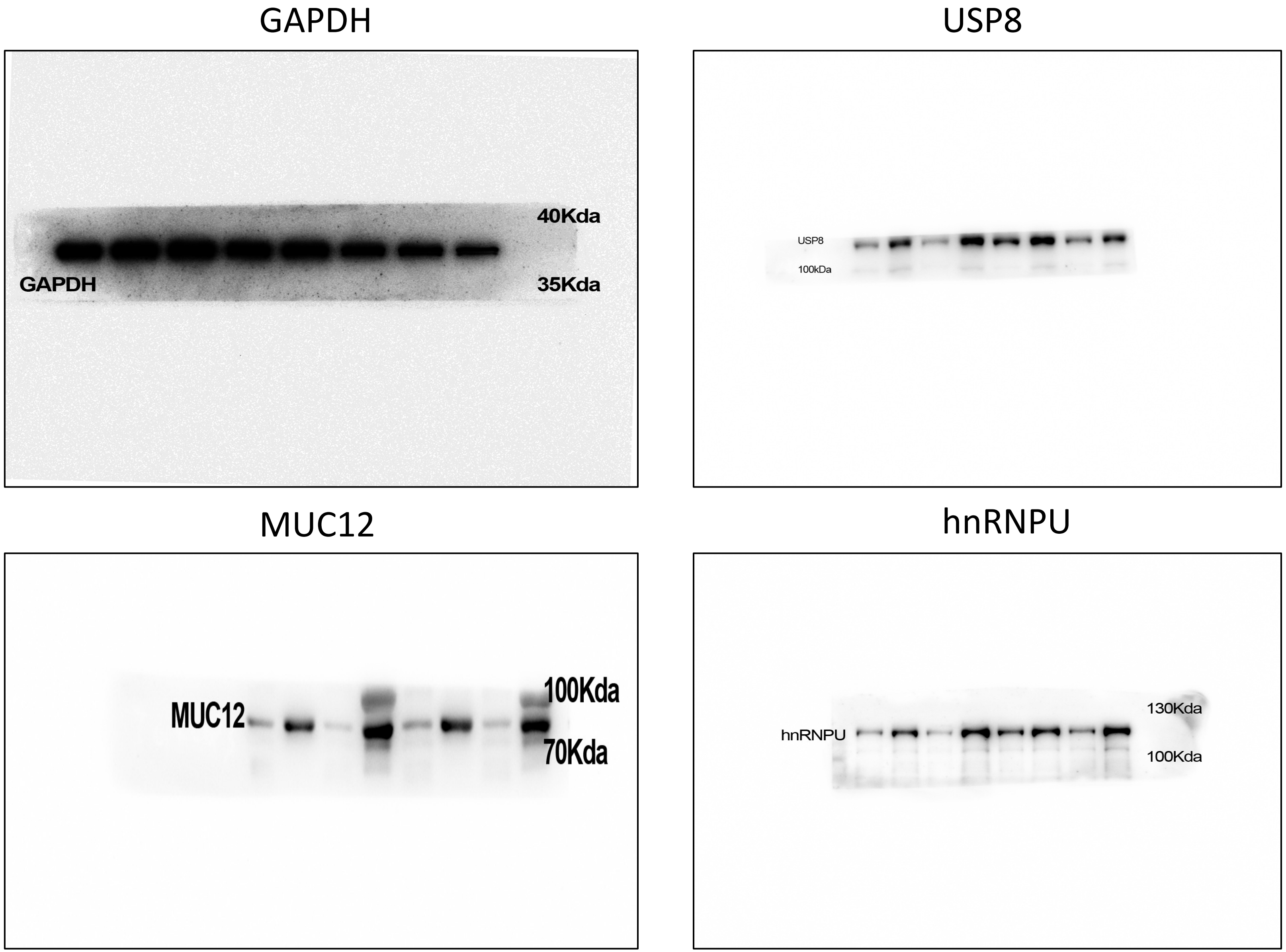
GAPDH
USP8
40Kda
GAPDH
35Kda
USP8
100kDa
MUC12
hnRNPU
100Kda
MUC12
70Kda
130Kda
hnRNPU
100Kda

Figure6 f

GAPDH

MUC12

786-O

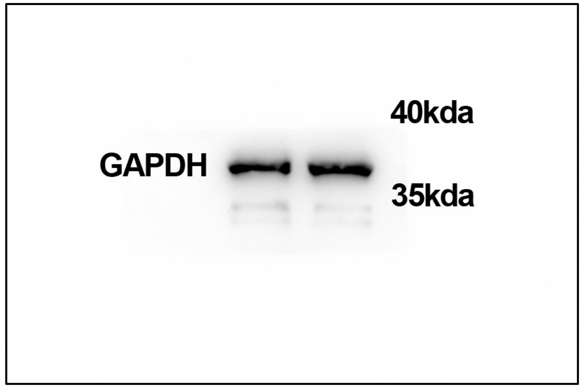

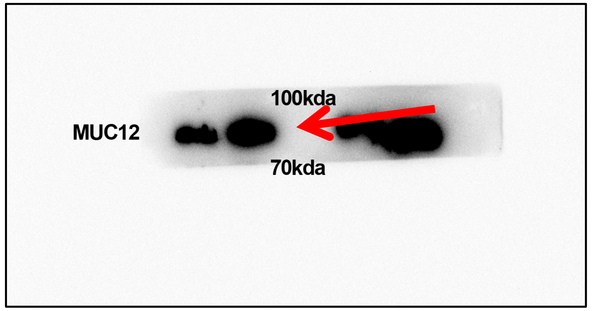

A498

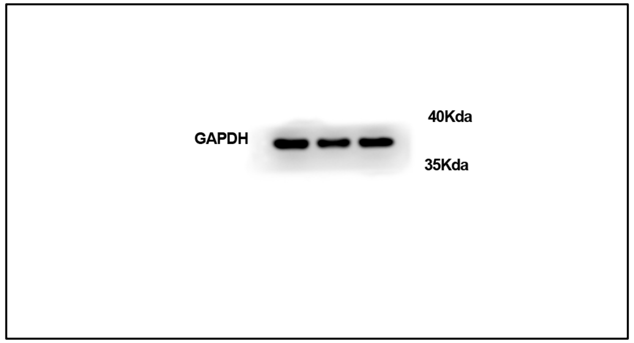

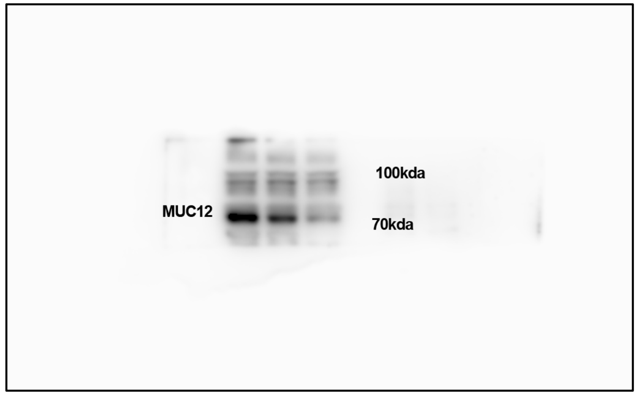

Figure6g

GAPDH MUC12

786-O

A498

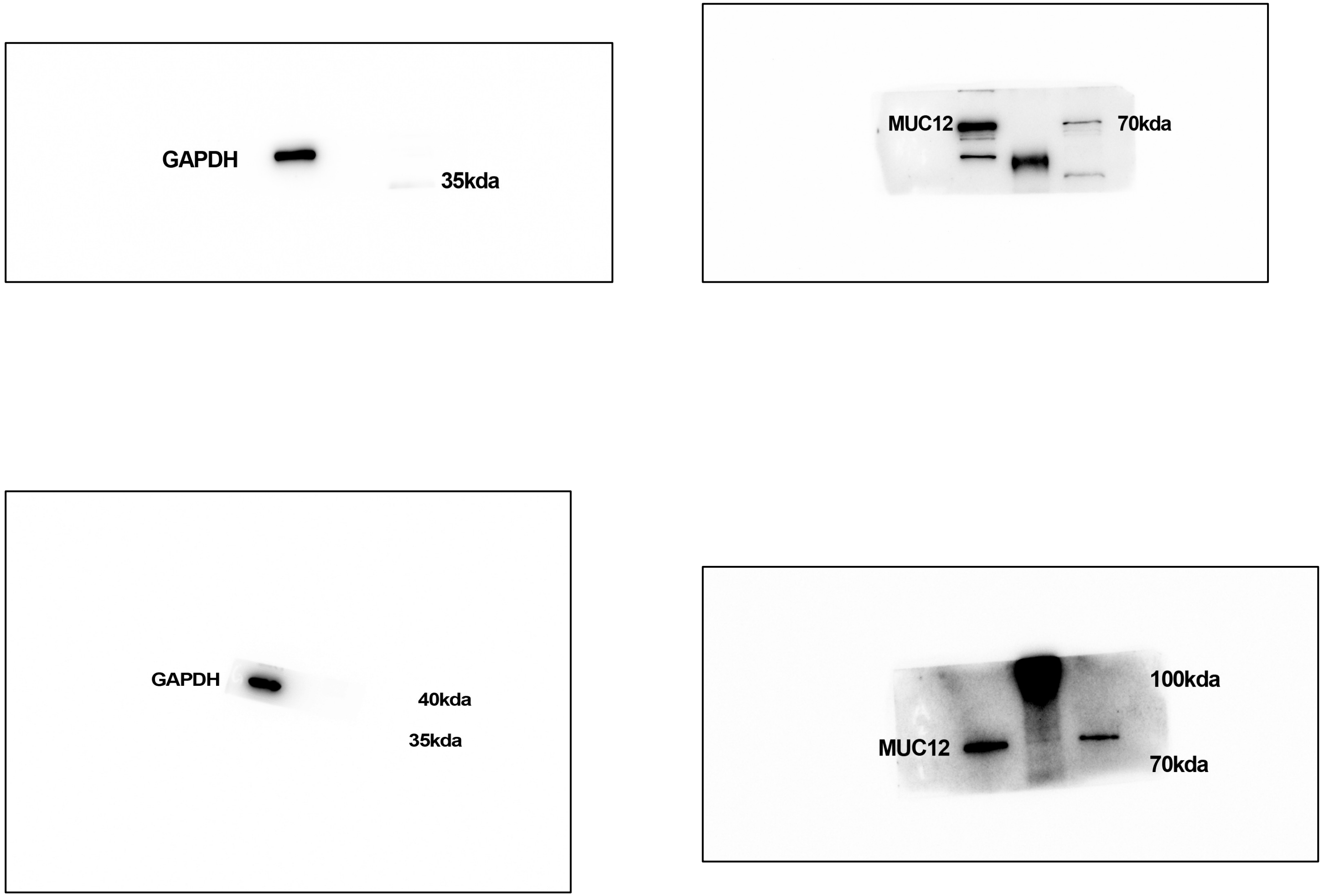

Figure6 h

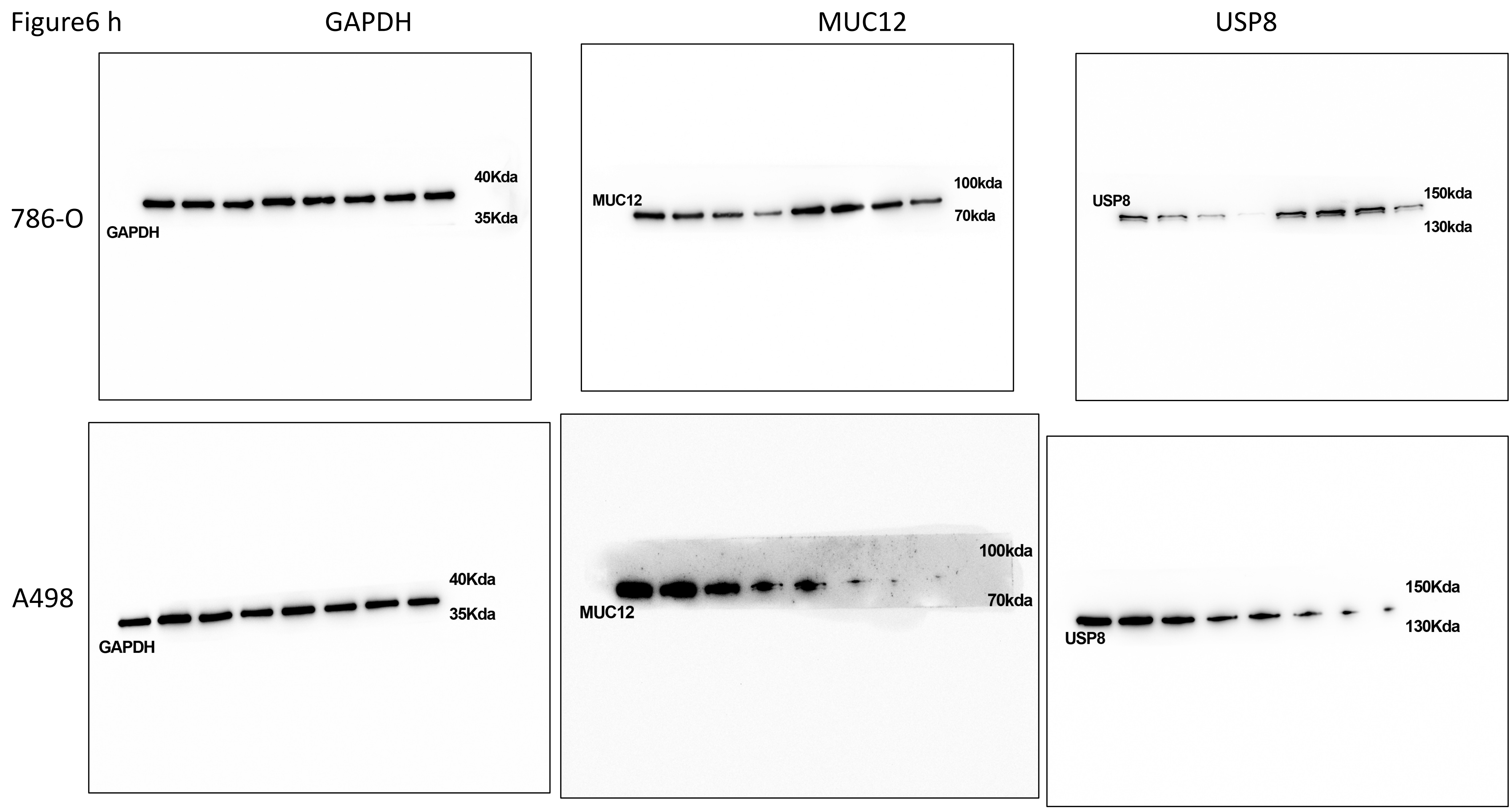
GAPDH
MUC12
USP8
786-O
A498
40Kda
35Kda
GAPDH
100kda
70kda
MUC12
150kda
130kda
USP8
40Kda
35Kda
GAPDH
100kda
70kda
MUC12
150Kda
130Kda
USP8

Figure6i A498

USP8

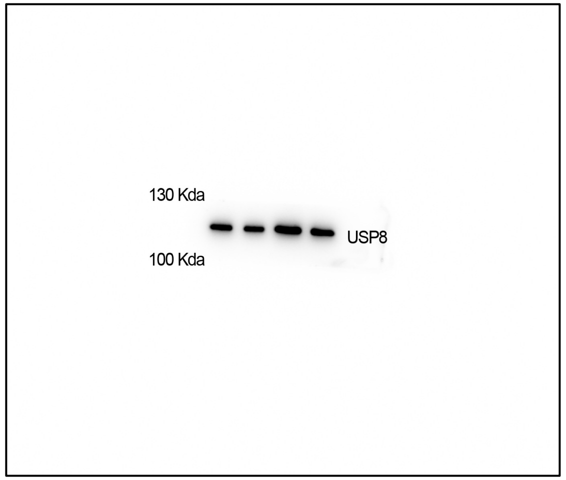

MUC12

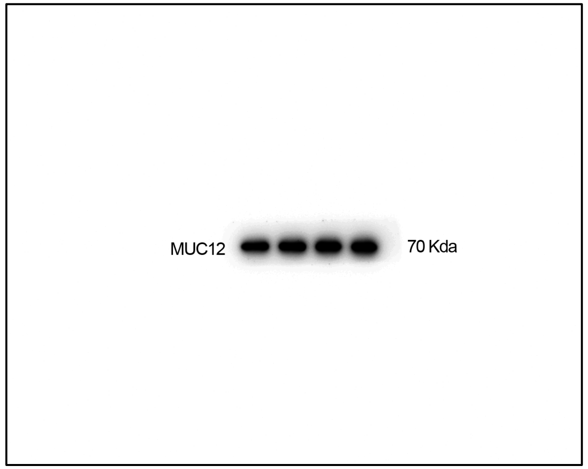

UB

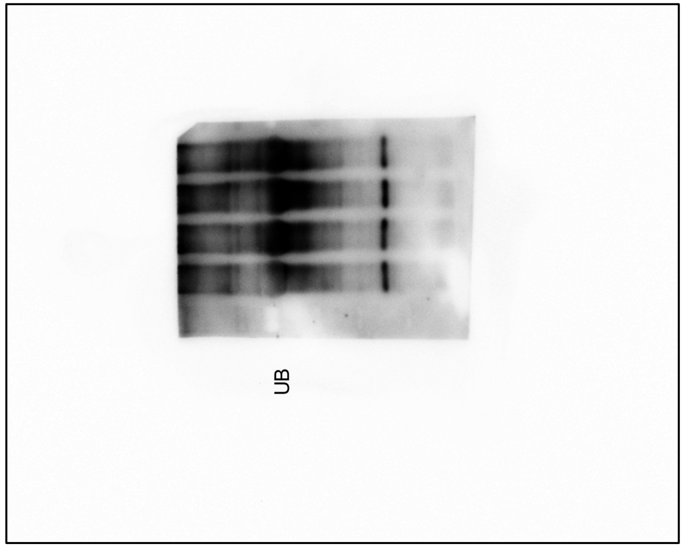

HA

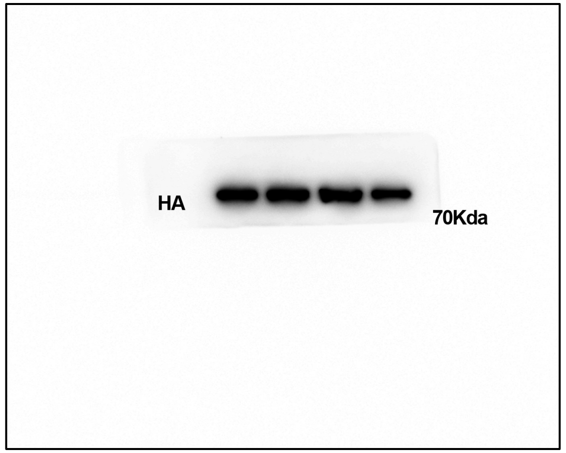

UB

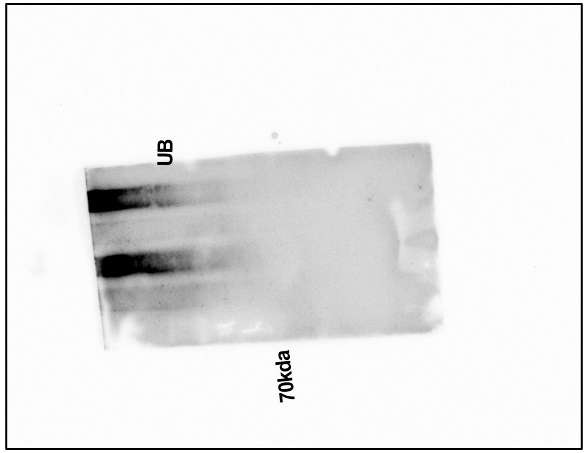

Figure6i 786-O

786-O

HA

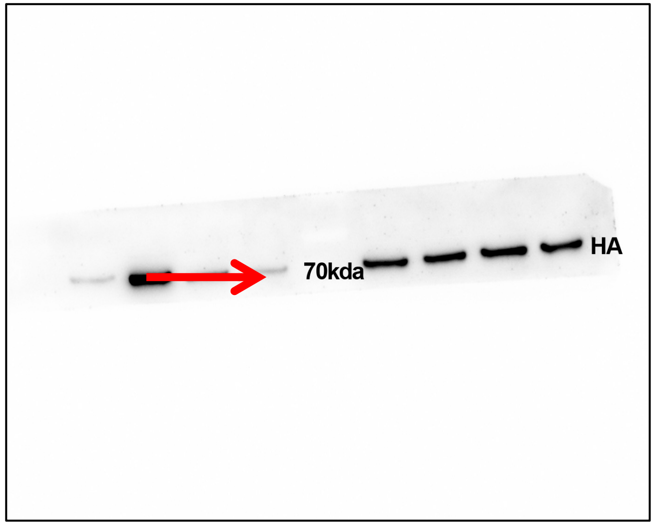

UB

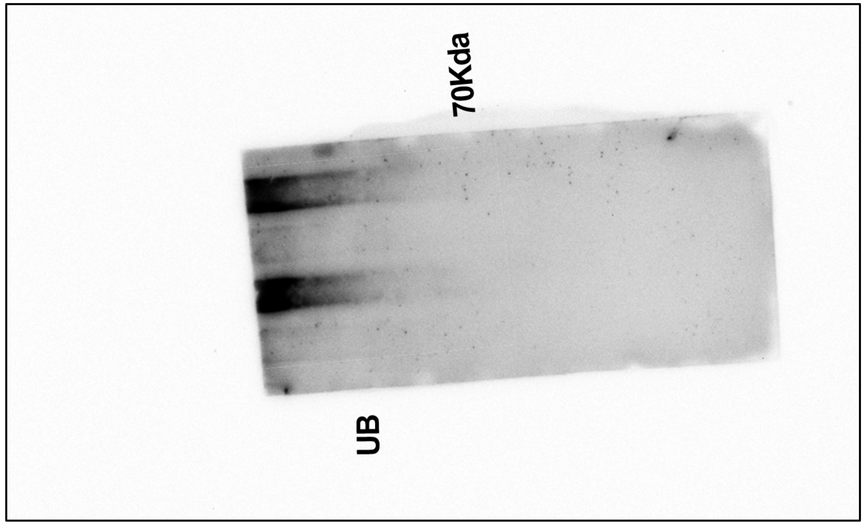

USP8

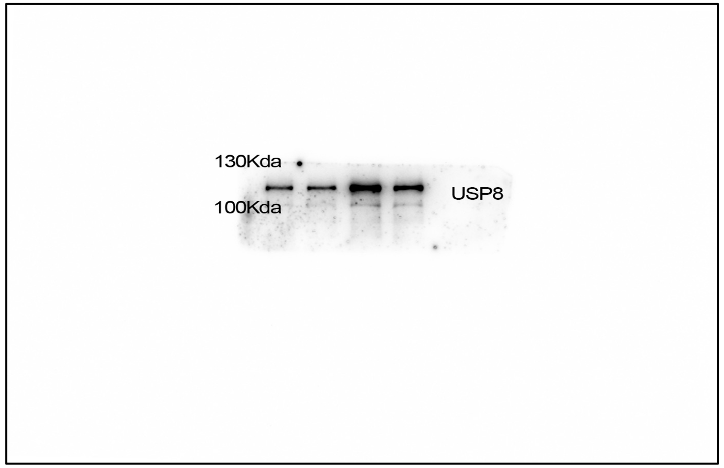

MUC12

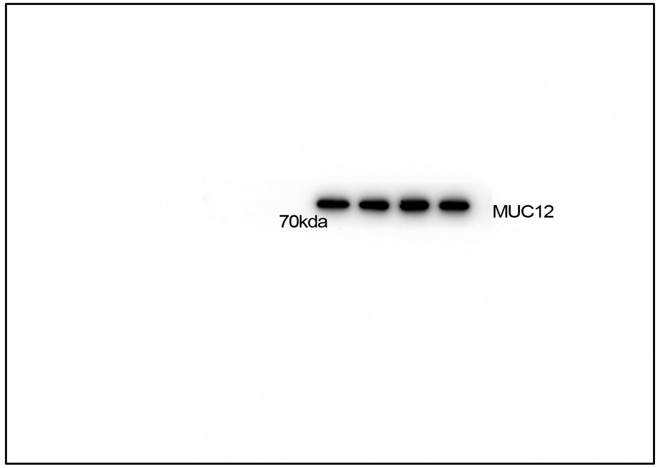

figure 6 j

GAPDH

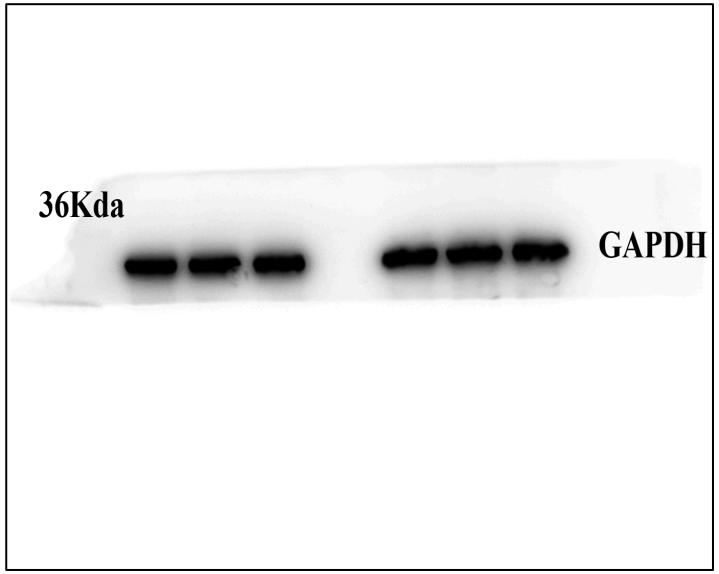

786-O-USP8

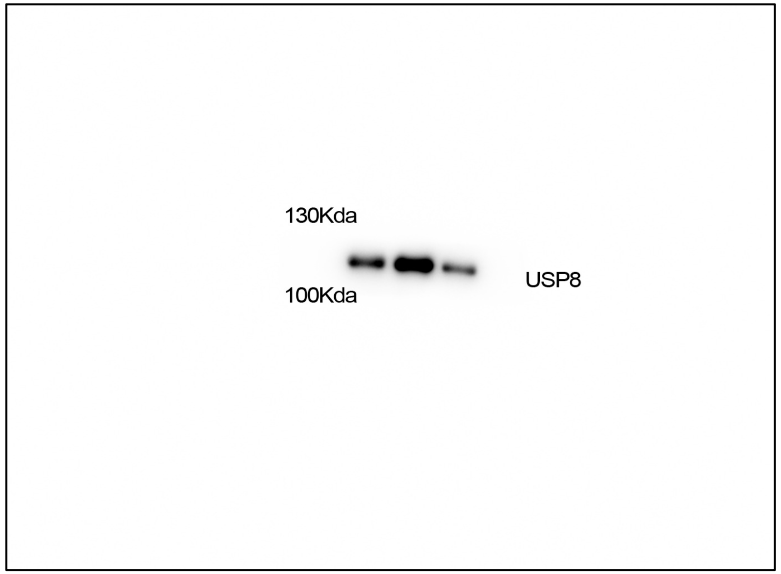

A498-USP8

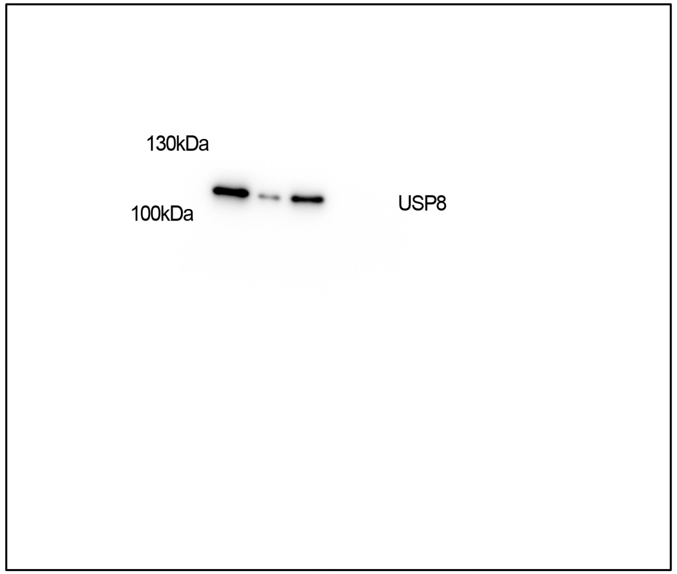

A498-MUC12

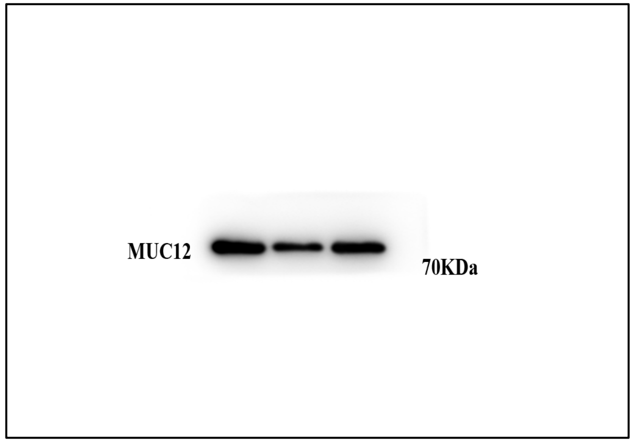

786-O-MUC12

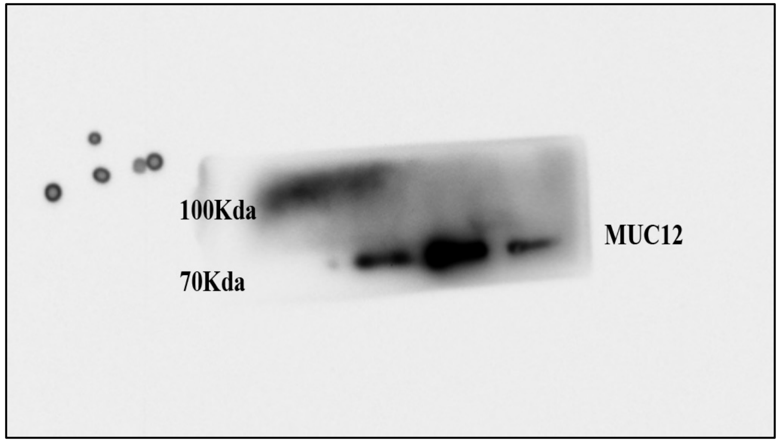

Figure8D

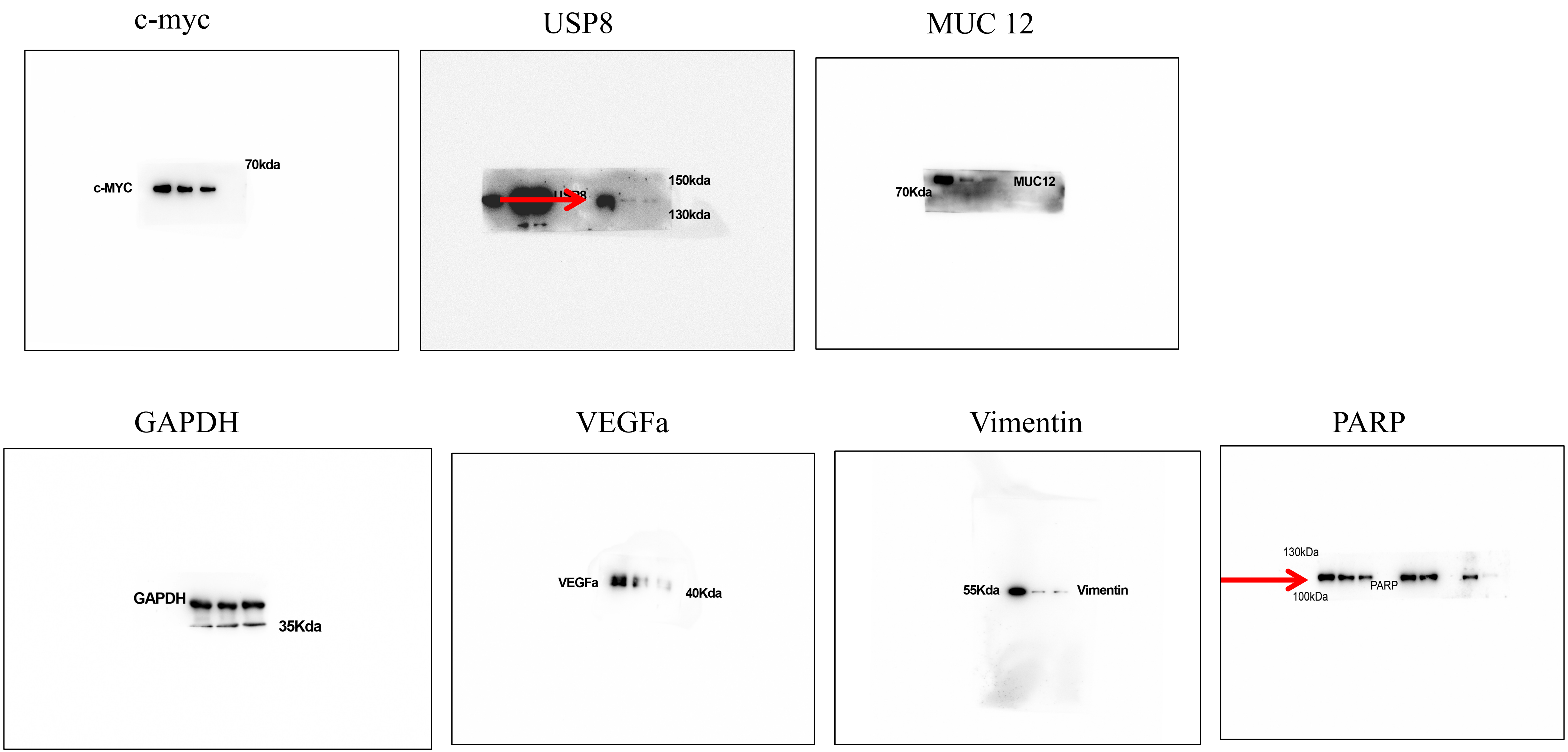
c-myc
USP8
MUC 12
c-MYC
70kda
150kda
130kda
70Kda
MUC12
GAPDH
VEGFa
Vimentin
PARP
GAPDH
35Kda
VEGFa
40Kda
55Kda
Vimentin
130kDa
PARP
100kDa

Figure8E

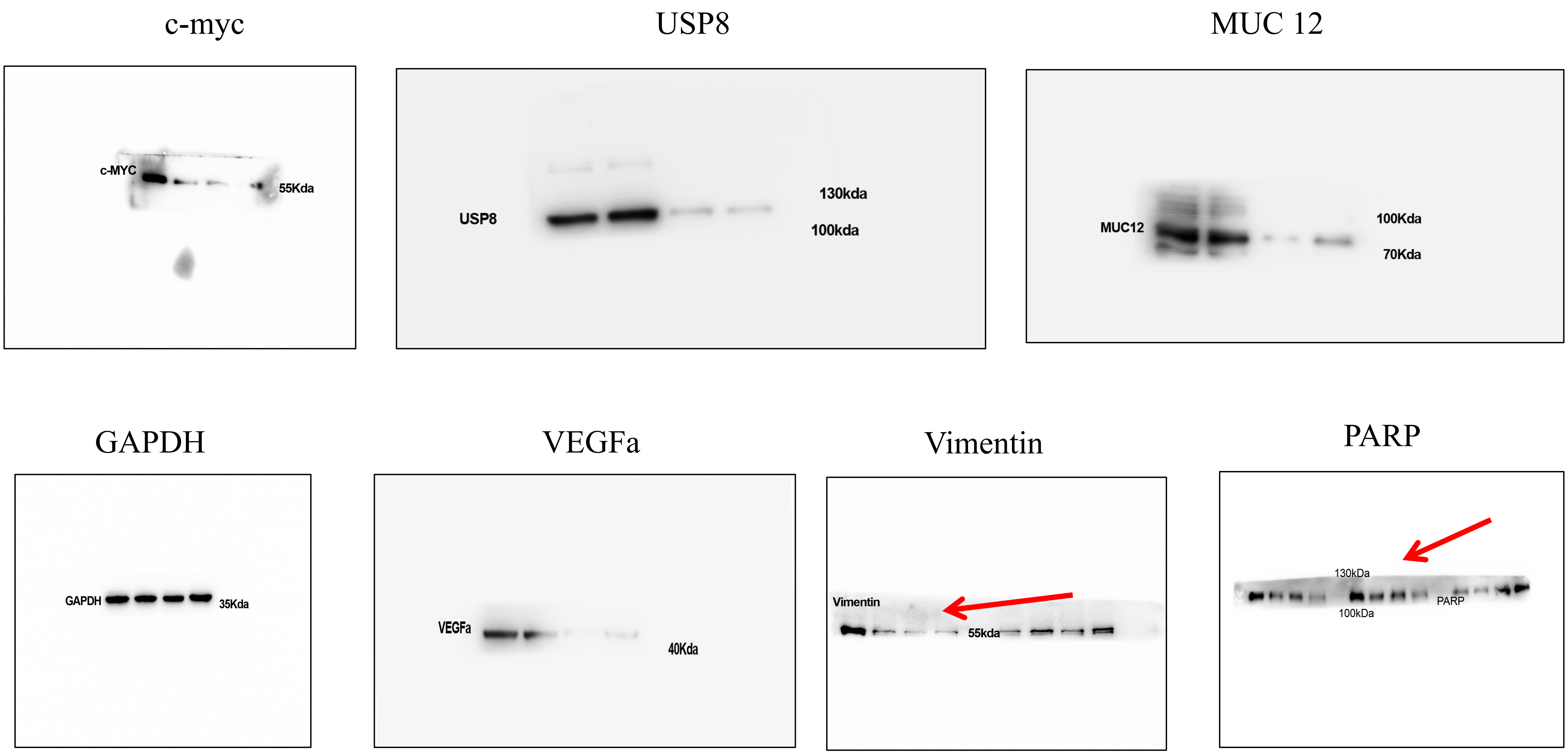
c-myc
c-MYC
55Kda
USP8
USP8
130kda
100kda
MUC 12
MUC12
100Kda
70Kda
GAPDH
GAPDH
35Kda
VEGFa
VEGFa
40Kda
Vimentin
Vimentin
55kda
PARP
130kDa
100kDa
PARP

Figure S4F

ACHN

GAPDH

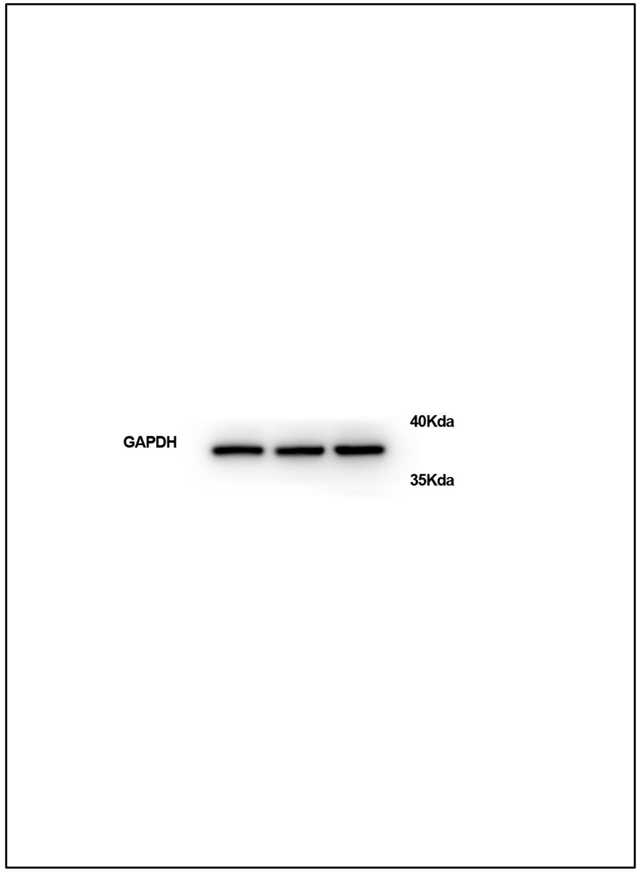

USP8

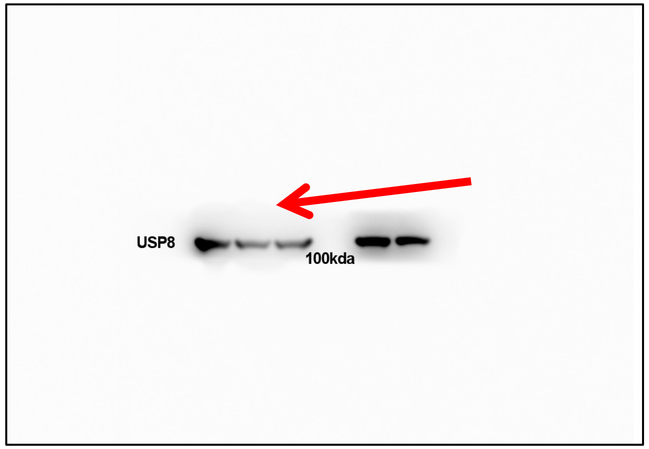

FigureS5B

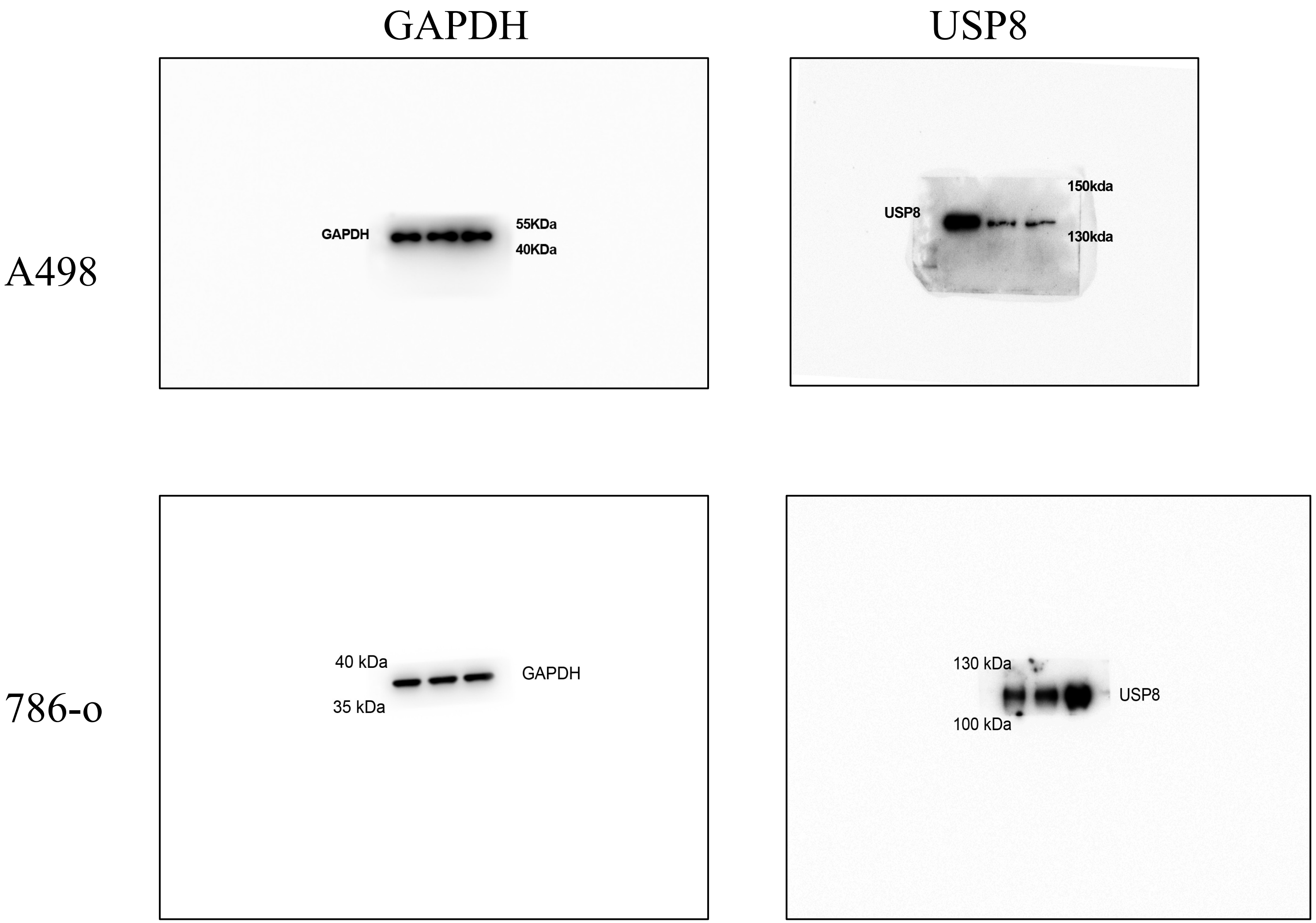

GAPDH
USP8
A498
GAPDH
55KDa
40KDa
USP8
150kda
130kda
786-o
40 kDa
35 kDa
GAPDH
130 kDa
100 kDa
USP8

FigureS5F

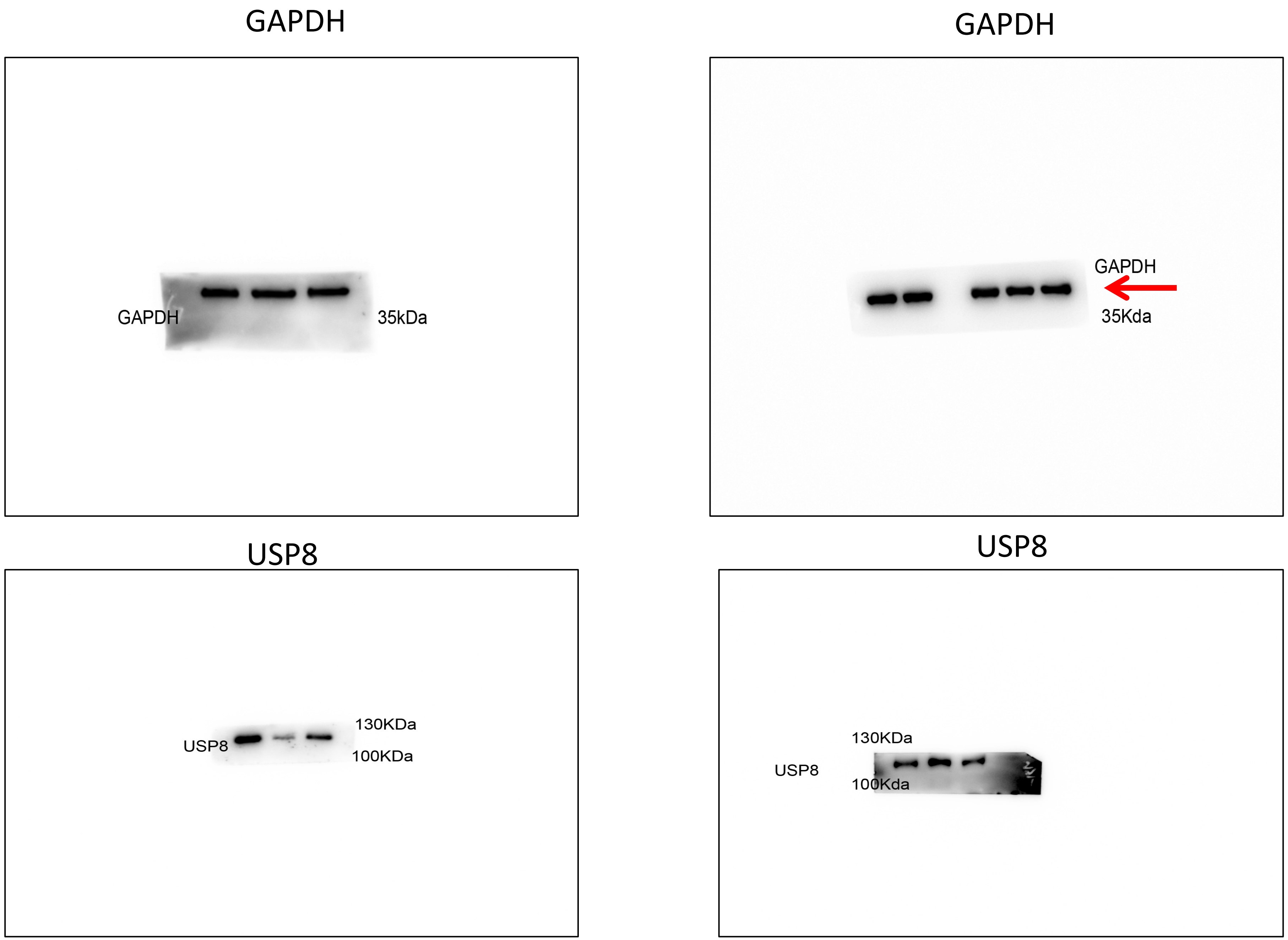
GAPDH
GAPDH
35kDa
GAPDH
GAPDH
35Kda
USP8
USP8
130KDa
100KDa
USP8
130KDa
USP8
100Kda

FigureS6D

GAPDH USP8 hnRNPU

A498

786-O

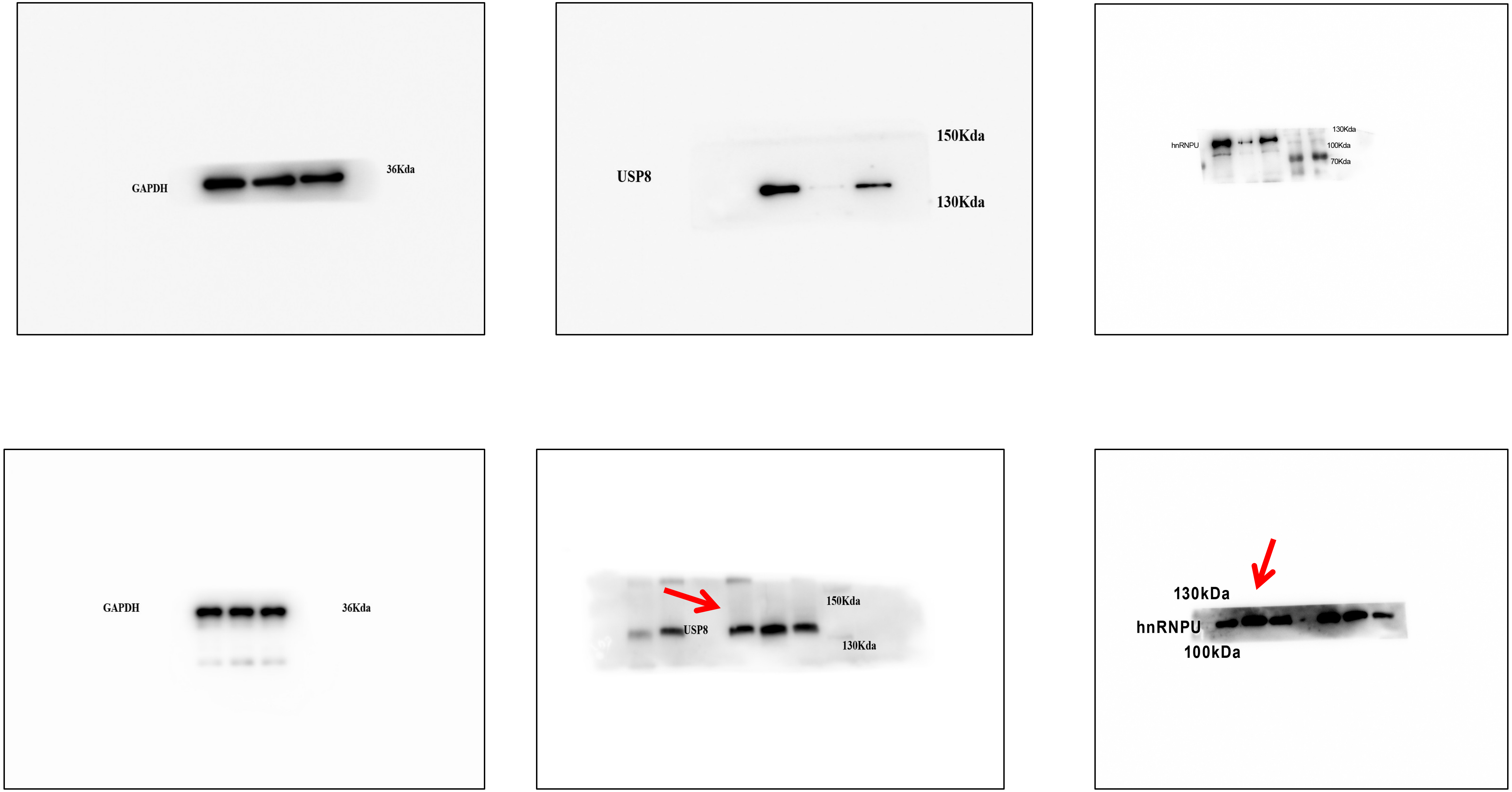

FigureS6F

A498

c-myc

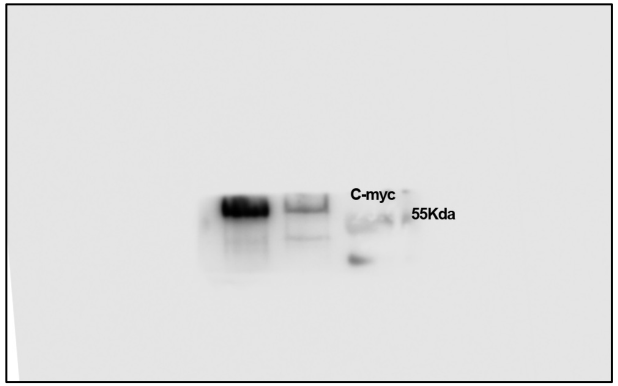

USP 8

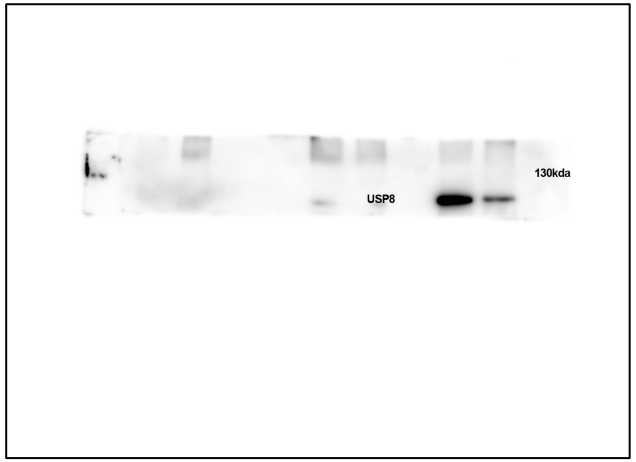

MUC 12

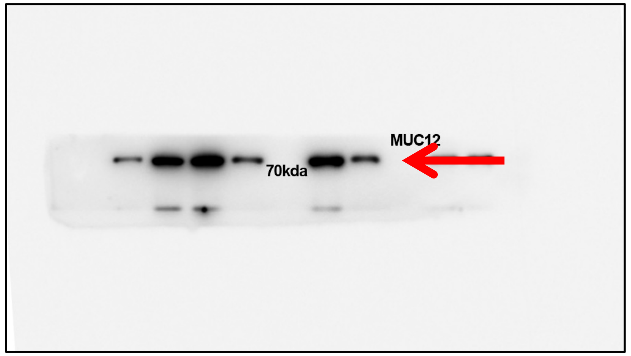

GAPDH

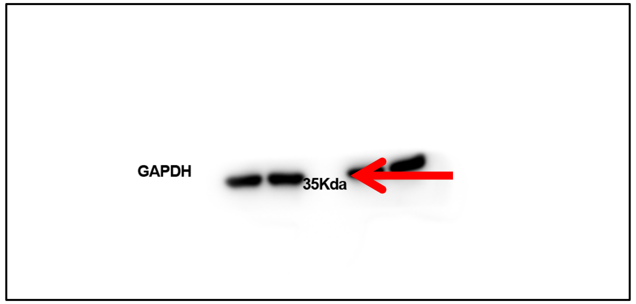

VEGFa

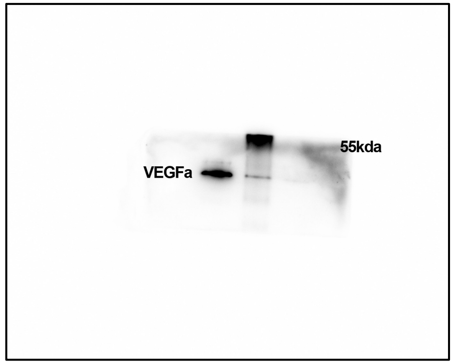

Vimentin

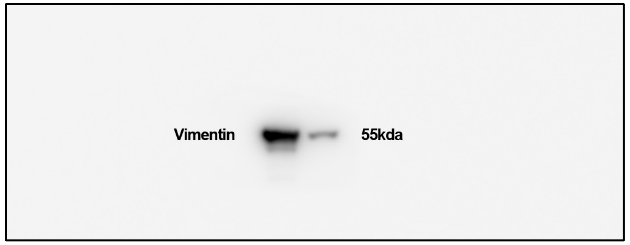

FigureS6F

786-O c-myc

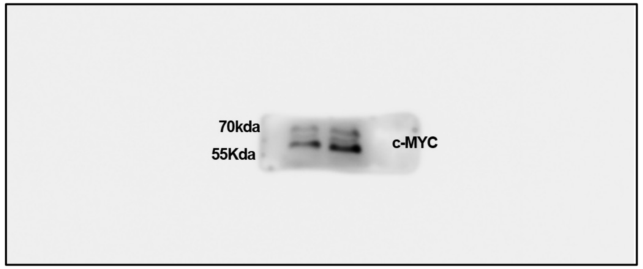

USP 8

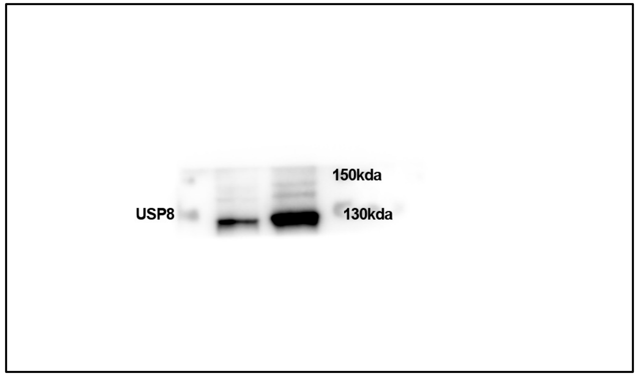

MUC 12

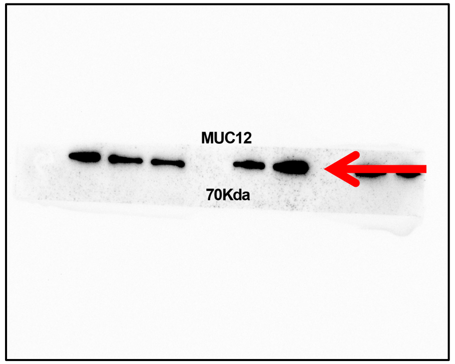

GAPDH

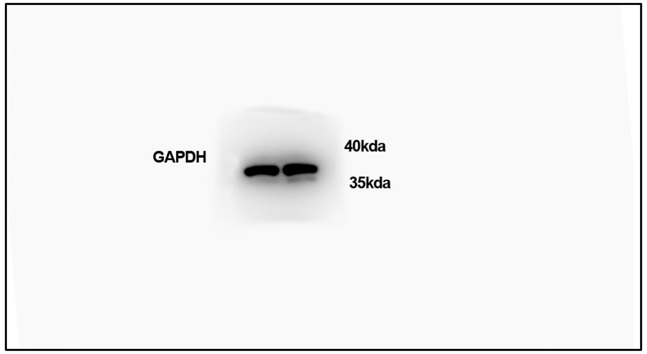

VEGFa

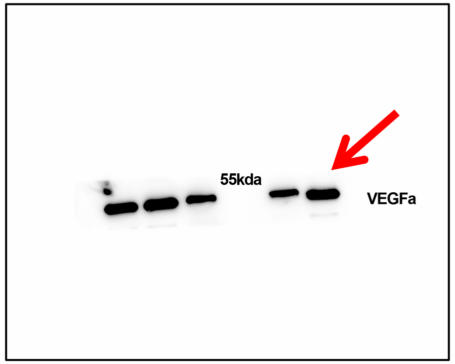

Vimentin

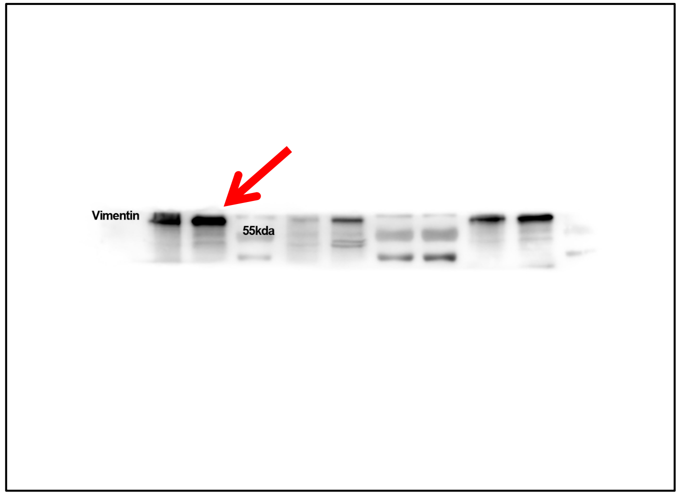

FigureS6G-A498

MUC12

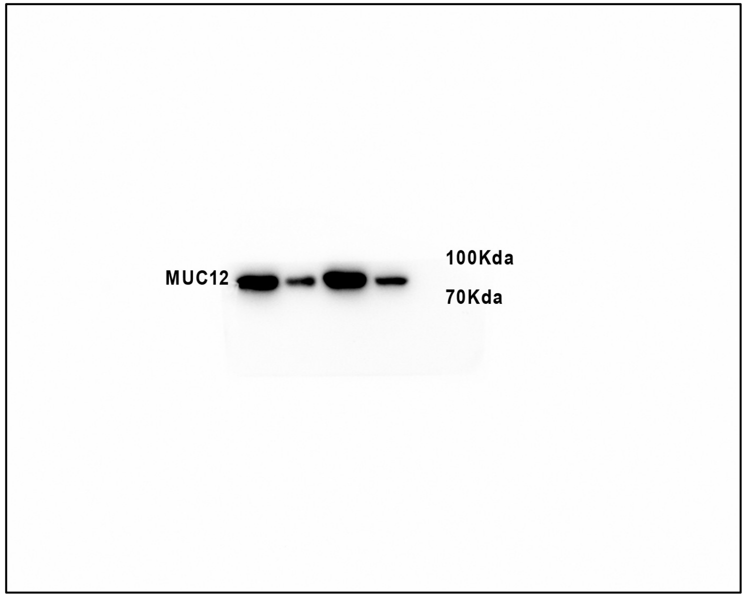

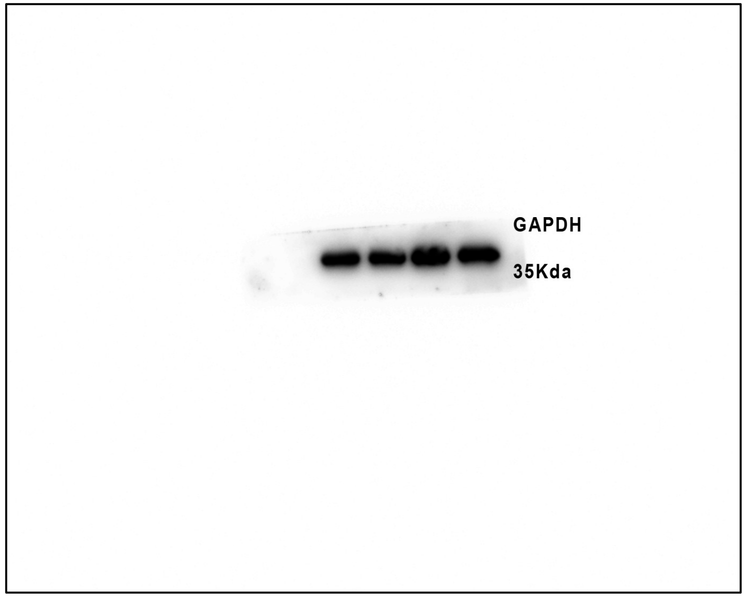

GAPDH

Beclin1

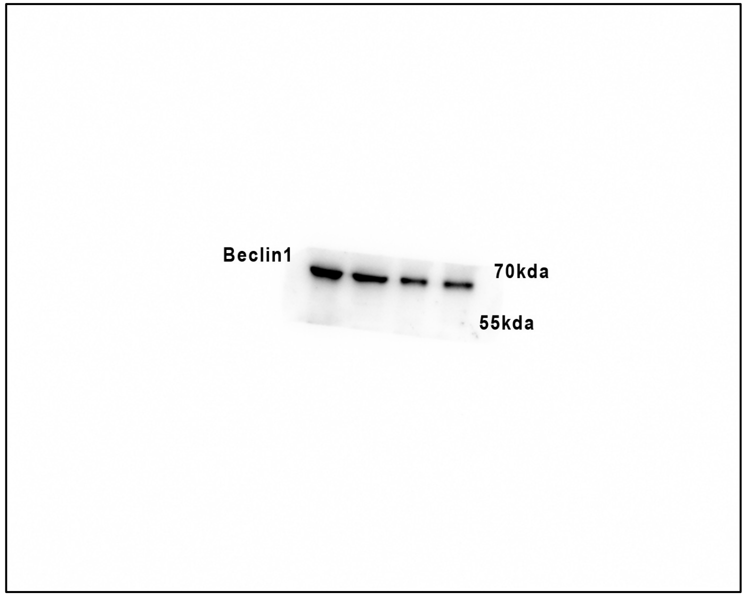

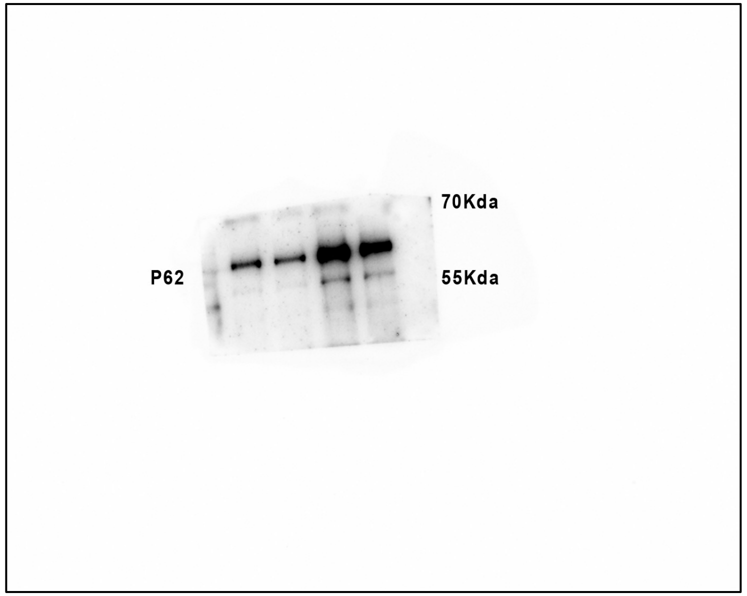

p62

FigureS6G-
786-O

MUC12

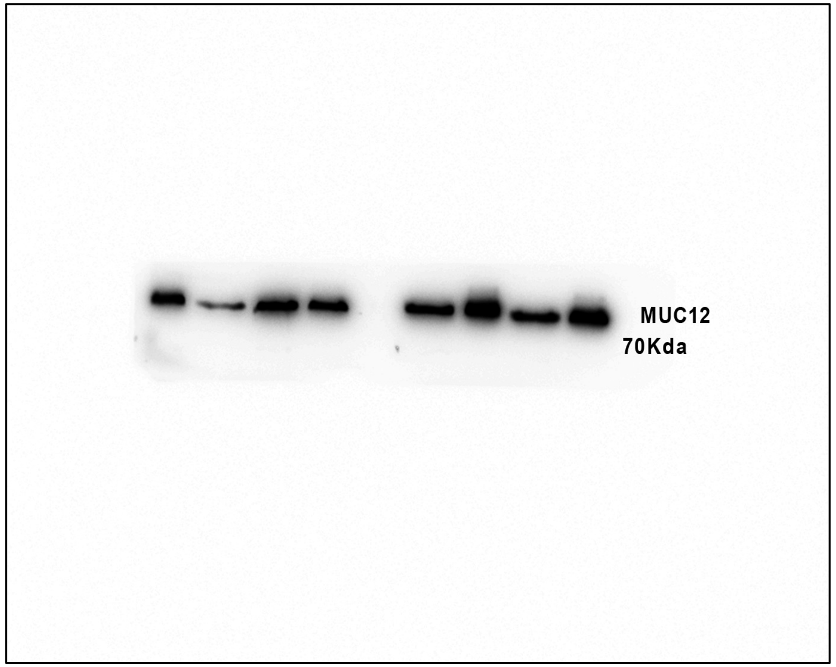

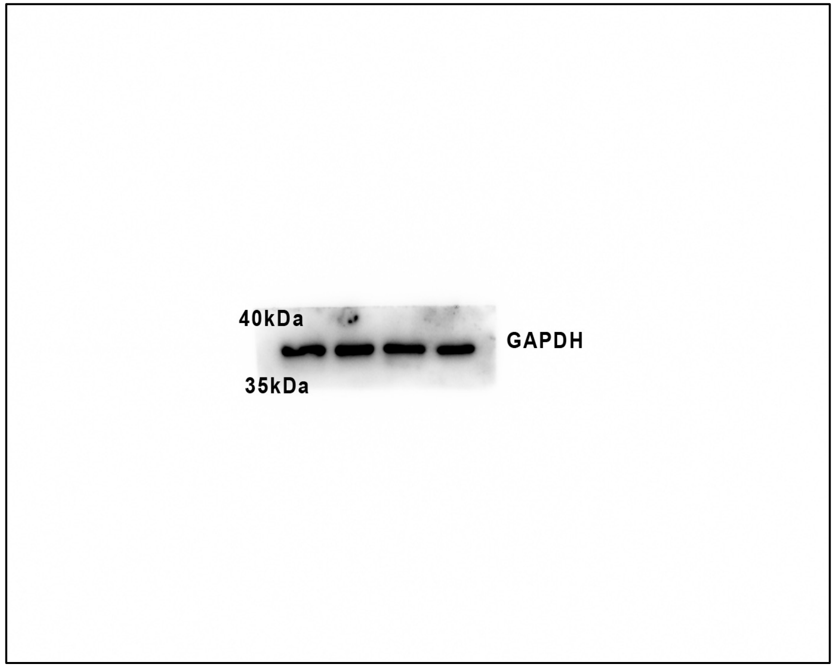

GAPDH

Beclin1

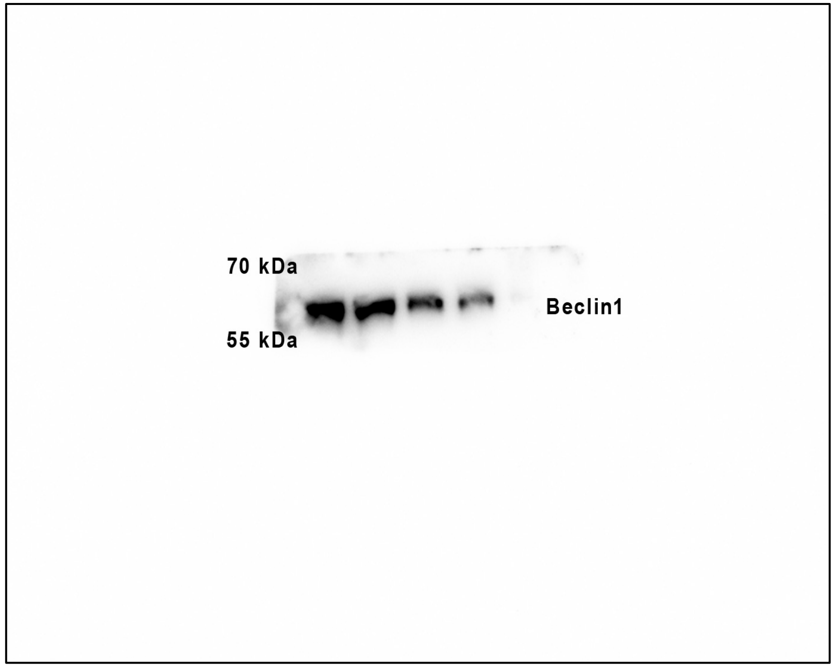

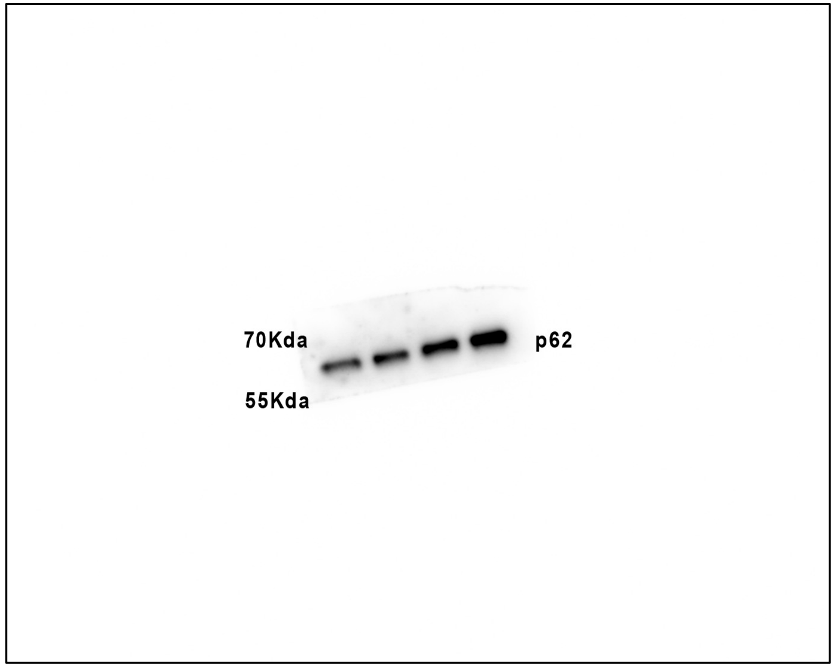

p62

FigureS6H

UB

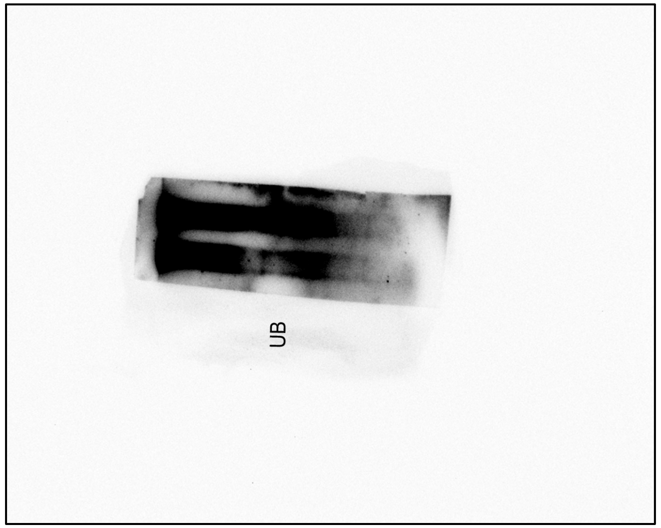

UB

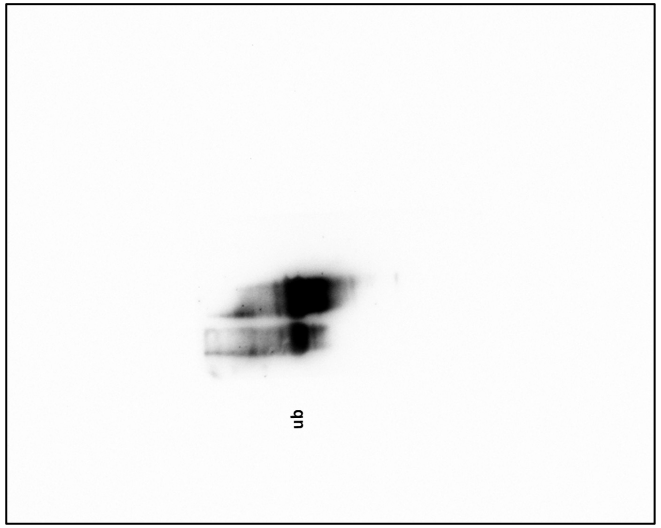

UB

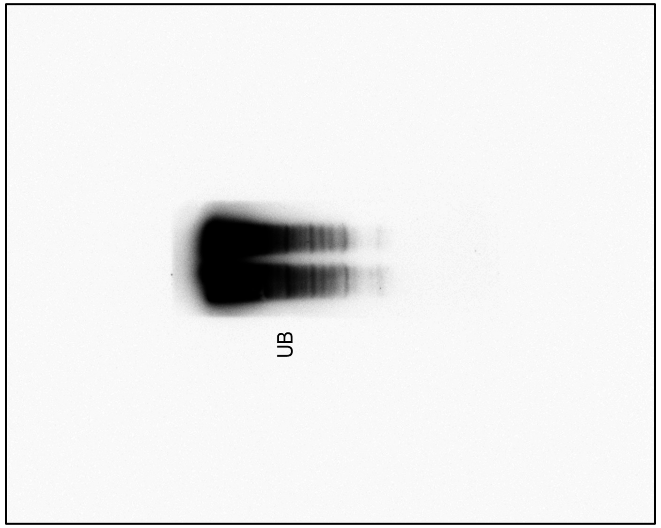

UB

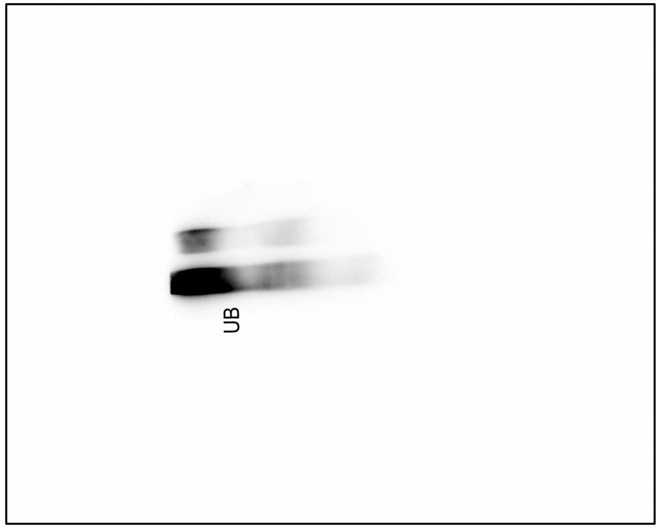

Figure S7 g

786-O

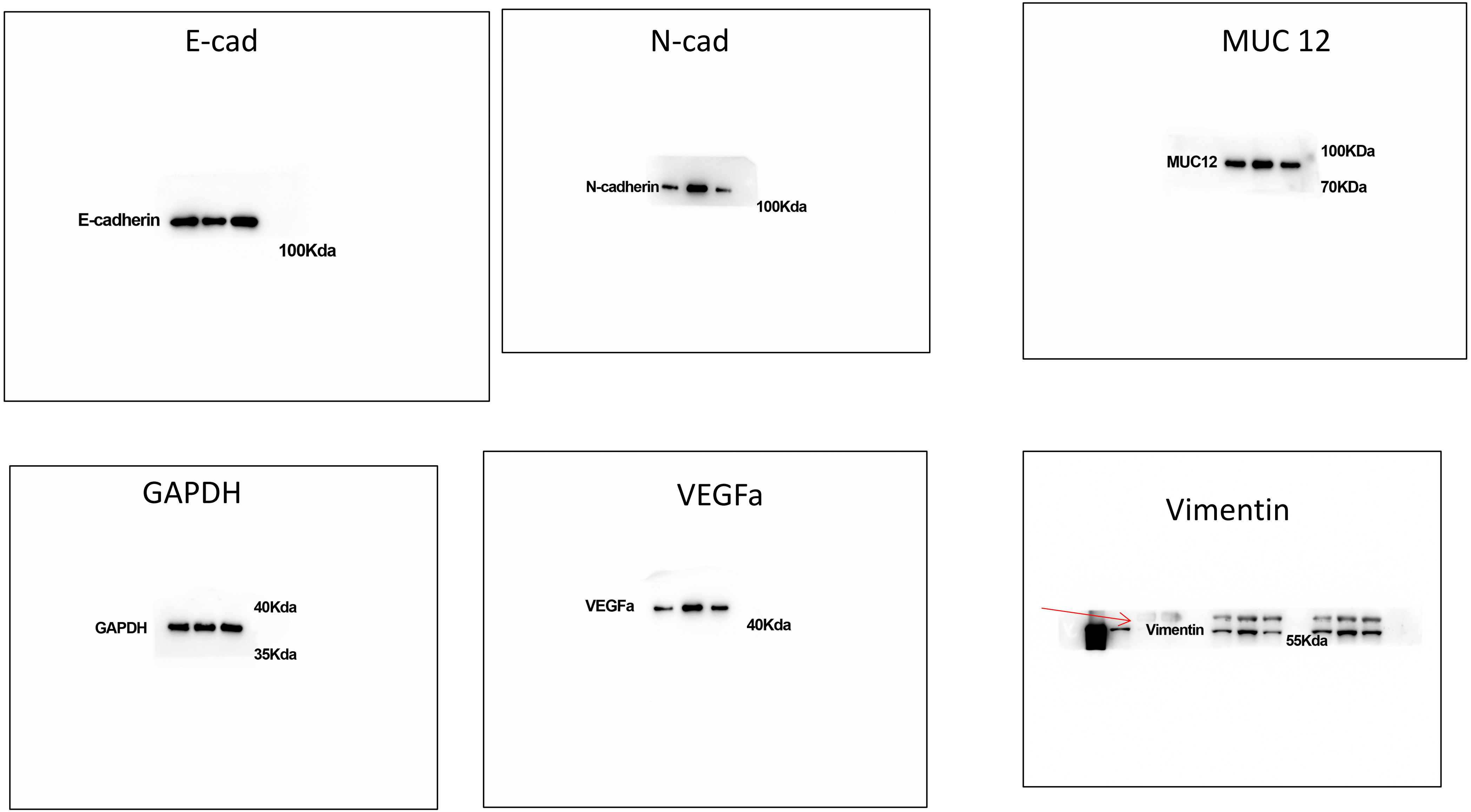

Figure S7 g

A498

E-cad

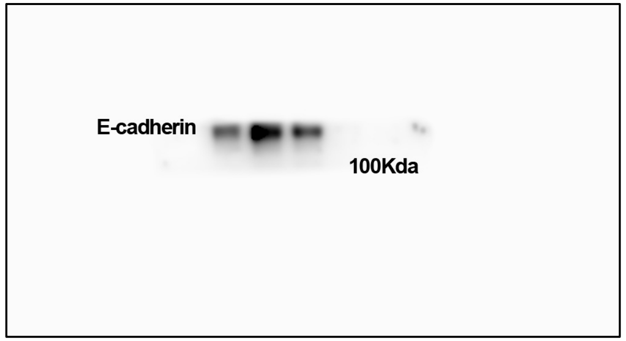

N-cad

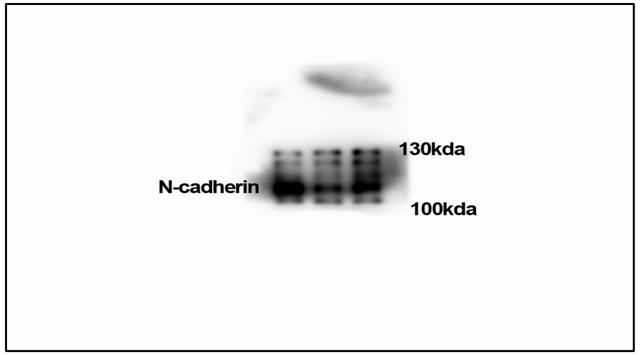

MUC 12

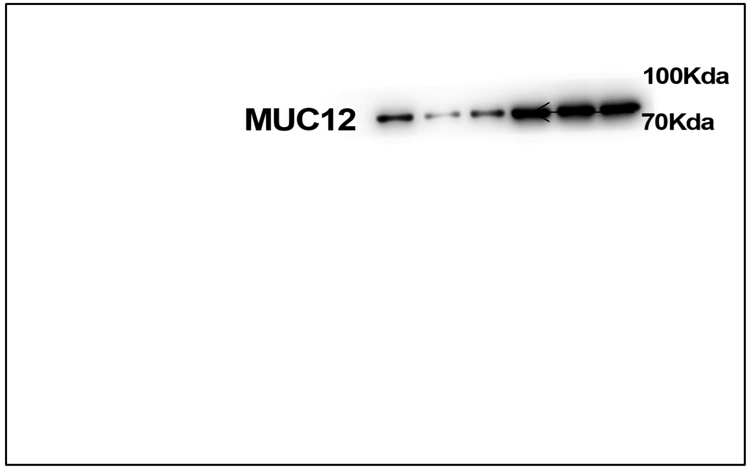

GAPDH

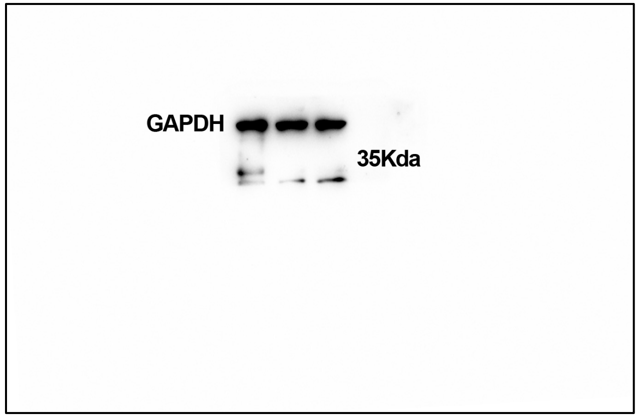

VEGFa

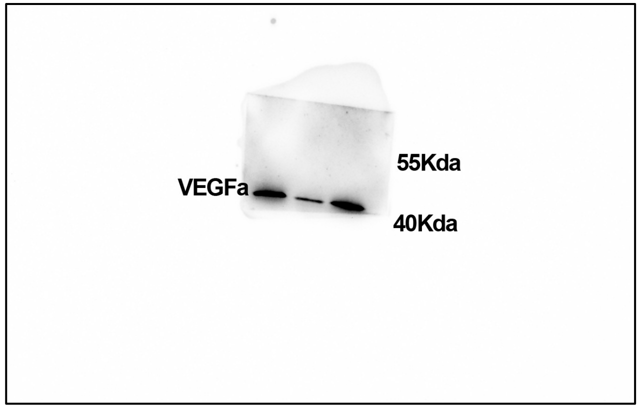

Vimentin

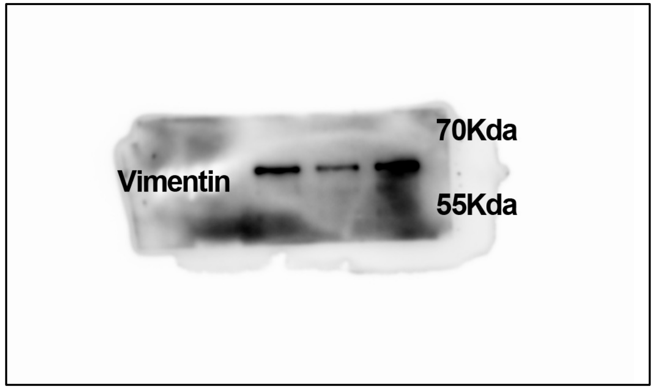

Supplement: Supplementary file 7 — Full Length Uncropped Original Western Blots [file 12276_2023_1010_MOESM7_ESM.pdf]
